# Inflammation-triggered sensing and miRNA immunotherapy with a wireless battery-free hydrogel bioelectronic patch

**DOI:** 10.1093/nsr/nwag409

**Published:** 2026-07-02

**Authors:** Zhenghan Shi, Yi Xu, Li-ang Zhou, Ye Liu, Feiyue Fang, Zijian An, Xin Li, Yanli Lu, Hao Wen, Lingkai Su, Qingjun Liu

**Affiliations:** Zhejiang Key Laboratory of Intelligent Sensing Technology and Advanced Medical Instrument, Key Laboratory for Biomedical Engineering of Ministry of Education, College of Biomedical Engineering & Instrument Science, Zhejiang University, Hangzhou 310027, China; Taizhou Key Laboratory of Medical Devices and Advanced Materials, Zhejiang Key Laboratory of intelligent Medical Decision Support, Taizhou Institute of Zhejiang University, Taizhou 318000, China; Innovation Center for Smart Medical Technologies & Devices, Binjiang Institute of Zhejiang University, Hangzhou 310053, China; Stomatology Hospital, School of Stomatology, Zhejiang University School of Medicine, Zhejiang Provincial Clinical Research Center for Oral Diseases, Key Laboratory of Oral Biomedical Research of Zhejiang Province, Engineering Research Center of Oral Biomaterials and Devices of Zhejiang Province, Hangzhou 310016, China; Zhejiang Key Laboratory of Intelligent Sensing Technology and Advanced Medical Instrument, Key Laboratory for Biomedical Engineering of Ministry of Education, College of Biomedical Engineering & Instrument Science, Zhejiang University, Hangzhou 310027, China; Zhejiang Key Laboratory of Intelligent Sensing Technology and Advanced Medical Instrument, Key Laboratory for Biomedical Engineering of Ministry of Education, College of Biomedical Engineering & Instrument Science, Zhejiang University, Hangzhou 310027, China; Zhejiang Key Laboratory of Intelligent Sensing Technology and Advanced Medical Instrument, Key Laboratory for Biomedical Engineering of Ministry of Education, College of Biomedical Engineering & Instrument Science, Zhejiang University, Hangzhou 310027, China; Zhejiang Key Laboratory of Intelligent Sensing Technology and Advanced Medical Instrument, Key Laboratory for Biomedical Engineering of Ministry of Education, College of Biomedical Engineering & Instrument Science, Zhejiang University, Hangzhou 310027, China; Zhejiang Key Laboratory of Intelligent Sensing Technology and Advanced Medical Instrument, Key Laboratory for Biomedical Engineering of Ministry of Education, College of Biomedical Engineering & Instrument Science, Zhejiang University, Hangzhou 310027, China; Zhejiang Key Laboratory of Intelligent Sensing Technology and Advanced Medical Instrument, Key Laboratory for Biomedical Engineering of Ministry of Education, College of Biomedical Engineering & Instrument Science, Zhejiang University, Hangzhou 310027, China; Zhejiang Key Laboratory of Intelligent Sensing Technology and Advanced Medical Instrument, Key Laboratory for Biomedical Engineering of Ministry of Education, College of Biomedical Engineering & Instrument Science, Zhejiang University, Hangzhou 310027, China; Stomatology Hospital, School of Stomatology, Zhejiang University School of Medicine, Zhejiang Provincial Clinical Research Center for Oral Diseases, Key Laboratory of Oral Biomedical Research of Zhejiang Province, Engineering Research Center of Oral Biomaterials and Devices of Zhejiang Province, Hangzhou 310016, China; Zhejiang Key Laboratory of Intelligent Sensing Technology and Advanced Medical Instrument, Key Laboratory for Biomedical Engineering of Ministry of Education, College of Biomedical Engineering & Instrument Science, Zhejiang University, Hangzhou 310027, China; Taizhou Key Laboratory of Medical Devices and Advanced Materials, Zhejiang Key Laboratory of intelligent Medical Decision Support, Taizhou Institute of Zhejiang University, Taizhou 318000, China; Innovation Center for Smart Medical Technologies & Devices, Binjiang Institute of Zhejiang University, Hangzhou 310053, China

**Keywords:** wireless bioelectronics, inflammation sensing, hydrogel interface, miRNA delivery, periodontal immunotherapy

## Abstract

Early diagnosis and timely intervention are crucial for the management of periodontal diseases driven by immune dysregulation. However, *in situ* sensing and drug delivery in the oral cavity remain challenging due to biological disparities between electronic devices and oral tissues. Here, we report a flexible, battery-free and hydrogel-based bioelectronics that integrates biological functions and wireless signal transmission for periodontal inflammation monitoring and localized microRNA (miRNA) immunomodulation. The hydrogel, synthesized from gelatin with bioactive peptide sequences cleavable by matrix metalloproteinases, responds specifically to protease activity associated with periodontal disease progression. Its enzymatic degradation is wirelessly transduced into quantitative radio-frequency signals of local immune activity, while simultaneously triggering the release of embedded miRNAs as critical immune regulators. *In vitro* and animal validations demonstrated diagnostic accuracy and therapeutic efficacy, leading to suppression of the inflammatory response. The combination of biosensing, wireless feedback and miRNA-based immunotherapy provides a closed-loop, adaptable platform for personalized management of periodontal and other inflammation-related disorders.

## Teaser

A bioresponsive hydrogel bioelectronic patch bridges wireless inflammation sensing and miRNA immunomodulation, enabling integrated monitoring and intervention for periodontal disease management.

## INTRODUCTION

Periodontitis is a common yet often underdiagnosed chronic inflammatory disease that affects the supporting structures of the teeth [1–3]. Chronic inflammation in periodontitis results from the immune system’s prolonged response to bacterial biofilms, leading to tissue destruction and eventual tooth loss [4,5]. Early diagnosis and timely intervention are crucial for controlling inflammation and restoring periodontal health. Currently, conventional diagnostic methods rely on clinical visual inspection and dental radiography, while treatment primarily involves periodontal scaling and systemic antibiotics [6,7]. However, these treatments often fail to prevent recurrence due to incomplete plaque removal, unhealthy lifestyles and poor patient compliance with oral hygiene [8]. Moreover, prolonged immune dysregulation can lead to continuous tissue destruction, necessitating long-term oral care and regular examinations [9,10]. Therefore, there is an urgent need for an electronic device capable of providing molecular-level insights and delivering immunotherapeutic interventions to achieve effective periodontitis management.

Traditional therapies for periodontitis based on oral medications or mouthwash often fail to maintain sustained drug concentrations at the infection site, limiting their therapeutic efficacy [11,12]. Consequently, biomaterial-based *in situ* delivery systems have emerged as a promising strategy for localized periodontal therapy by enabling sustained or on-demand therapeutic release [13–15]. Antibiotics such as metronidazole and minocycline have been incorporated into commercially available hydrogel systems for topical or injectable use in periodontal pockets [16,17]. In particular, since immune responses play a key role in periodontitis pathogenesis, approaches that directly modulate the local immune microenvironment have attracted increasing attention [18]. Immune-modulatory agents such as cytokines and exosomes have been explored for immune modulation [19–21], but their complex preparation, limited stability and cost might hinder widespread applications. In contrast, microRNAs (miRNAs), small RNA molecules that regulate gene expression, represent an emerging class of biomolecular therapeutics [22–24]. MiRNAs can modulate multiple genes involved in immune responses and suppress pro-inflammatory mediators [25–27]. They can also be synthetically produced at scale, but challenges remain in selecting effective immune-regulatory miRNA, and achieving efficient delivery to target cells.

Effective periodontal management also requires dynamic and precise monitoring of disease biomarkers. Real-time sensing and integration of biochemical information into digital health systems are highly desirable for personalized management of chronic periodontal disease. However, due to the mechanical and physicochemical disparities between living tissues and synthetic devices, developing biocompatible and stable bioelectronic interfaces remains challenging. Recent advances in flexible electronics integrated with biochemical sensors have shown promise in wearable health monitoring [28]. Hydrogels, with their high-water contents and microporous structures, provide a physiologically compatible interface between bioelectronic devices and biological tissues [29–31]. Their programmable responsiveness to biochemical stimuli makes them attractive for both sensing and therapeutic applications [32,33]. While many hydrogel-based sensors rely on optical assays such as colorimetric or fluorescence analysis [34–36], radio-frequency techniques provide a wireless and battery-free detection approach [37–39]. By translating biochemical processes into quantifiable wireless signals, radio-frequency resonators enable non-invasive and dynamic monitoring of local biological microenvironments. Meanwhile, closed-loop bioelectronic platforms that integrate sensing with therapeutic actuation have advanced adaptive treatment strategies [40,41], but the direct coupling of molecular-level inflammatory sensing with localized immunotherapeutic delivery remains largely unexplored.

Herein, we report a hydrogel-based wireless inflammation-responsive electronic system (termed WIRES) that directly interfaces with the immune microenvironment to enable both biosensing and immunomodulatory therapy. The system integrates a matrix metalloproteinase (MMP)-cleavable hydrogel with a soft radio-frequency circuit to convert protease activity into wireless electromagnetic signals. The hydrogel matrix also acts as a carrier for inflammation-triggered release of therapeutic miRNAs that reprogram macrophages toward anti-inflammatory phenotypes. This hydrogel-based bioelectronic platform establishes a closed-loop paradigm that couples immune monitoring with bioresponsive therapy, offering a promising strategy for personalized and minimally invasive management of periodontal inflammation.

## RESULTS

### Overall design of the hydrogel-based WIRES

**Figure 1.**
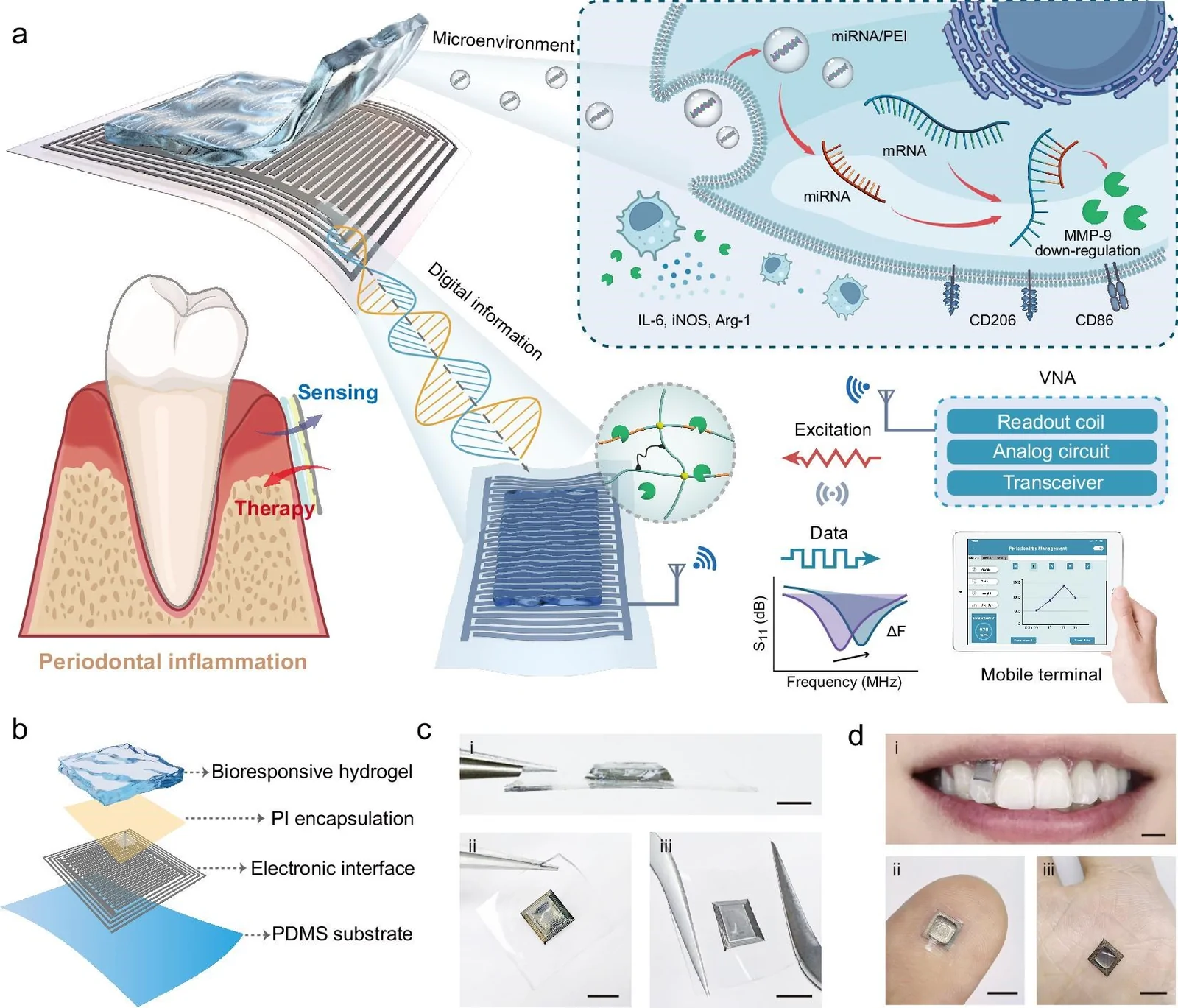
Design of the hydrogel-based WIRES. (a) Schematic of WIRES for periodontal sensing and immunotherapy. (b) Exploded view of WIRES. (c) Optical image of various WIRES device configurations. Scale bars: (i) 2 mm; (ii) 4 mm; (iii) 4 mm. (d) WIRES attached on different tissues. Scale bars: 5 mm.

Periodontitis is a chronic inflammatory disease driven by microbial infection and the recruitment of immune cells that promote tissue destruction. While periodontal inflammation is commonly managed through mechanical cleaning and systemic antibiotics, long-term monitoring and targeted modulation remain challenging due to the dynamic nature of the periodontal microenvironment. To address this, we developed a battery-free, hydrogel-based WIRES platform, designed to bridge biological information with digital signals (Fig. 1a). The system comprised a bioresponsive hydrogel that interfaced with the immune microenvironment, and a soft, all-printed electronic circuit enabling wireless signal transduction and digital communication.

The hydrogel served as the core bioactive matrix of the platform, incorporating peptide sequences selectively cleavable by MMP-9, a clinically validated biomarker of periodontal inflammation [42–44]. When inflammation occurred in the periodontal microenvironment, immune cells were activated and MMP-9 was released, which triggered progressive degradation of the hydrogel through cleavage of the peptide. This process led to the responsive release of the miRNA nanoparticles loaded in the hydrogel, which entered cells and modulated cell polarization. Simultaneously, structural degradation of the hydrogel altered the local dielectric environment, inducing a capacitance change in the interdigitated electrode. This change was converted into a frequency shift through the integrated resonant circuit and wirelessly transmitted to a mobile terminal for remote monitoring through inductive coupling.

The wireless interface adopted a multilayer soft bioelectronic architecture (Fig. 1b; Fig. S1 provided the fabrication details). Aerosol jet printing was utilized to obtain high-precision miniaturized circuits on a flexible polydimethylsiloxane (PDMS) substrate. The inductive antenna and capacitive interdigitated electrode were printed with silver nanoparticles (AgNPs), while polyimide (PI) was used for the encapsulation and dielectric layer. The hydrogel composite, including miRNA drug loading, was synthesized through *in situ* polymerization on the electrodes. As displayed in Fig. 1c, the size, entire thickness and total mass of WIRES were 5 mm × 5 mm, 1.5 mm and 35 mg, respectively. It was miniaturized, lightweight and capable of mechanical bending and stretching, which allowed it to be senselessly attached to the periodontal tissues inside the oral cavity (Fig. 1d).

The multifunctionality of WIRES arose from the interactions between the electronic interface, bioactive hydrogel and the periodontal microenvironment. The electronic interface employed the wireless and battery-free resonant circuit design, which eliminated the need for additional peripheral circuits or external power sources as with conventional electronic systems. The hydrogel biointerface not only acted as a biorecognition element for MMP-9 sensing, but also provided a porous matrix for controlled release of immunomodulatory miRNAs, which improved their stability. Additionally, its excellent biocompatibility and viscoelasticity enabled robust coupling with wet tissues, making it a versatile platform for both wearable and implantable intraoral applications.

### All-printed flexible electronic interface

To achieve reliable wireless signal transduction and accurate biosignal detection, precise fabrication of the flexible electronic interface was essential. Here, the flexible interface was fabricated using aerosol jet printing, which provided high reliability and micron-level resolution (Fig. S2). Aerosol jet printing was selected because it combined high-resolution patterning, non-contact fabrication on flexible substrates and compatibility with multiple functional inks [45,46], which were important for constructing the miniaturized electronic interface. Compared with commonly used inkjet printing or screen printing, it was better suited for fabricating fine interdigitated electrodes and antenna structures while enabling sequential deposition of conductive silver ink and dielectric PI ink within the same fabrication framework.

**Figure 2.**
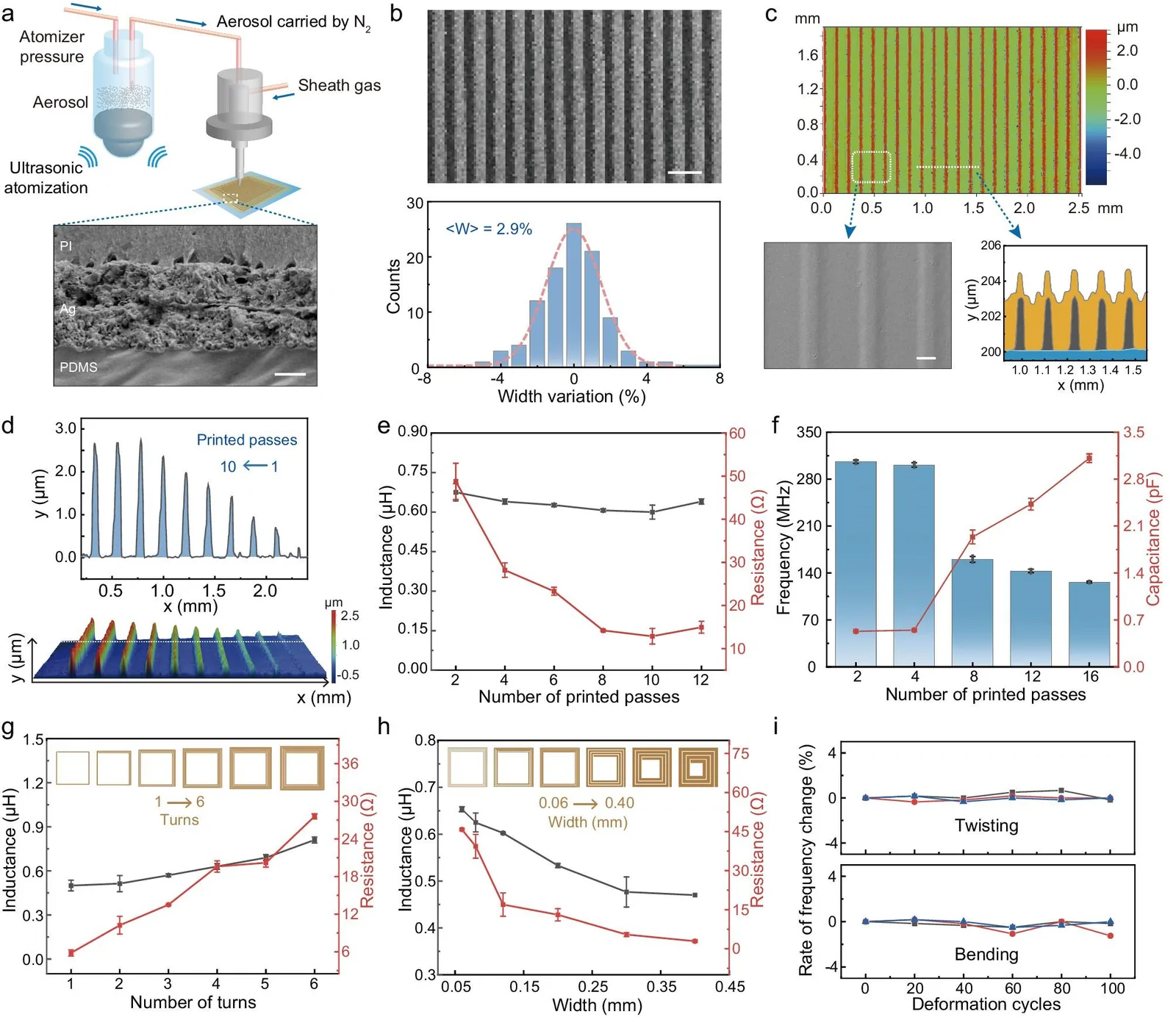
Aerosol jet printed flexible electronic interface. (a) Illustration of the fabrication process of the flexible electronic interface using aerosol jet printing, including a cross-sectional SEM image of the printed interface. Scale bar: 1 μm. (b) SEM image of the printed interdigital electrode and the variation in width of the printed digits. Scale bar: 200 μm. (c) Optical profilometry and SEM characterization of aerosol jet-printed PI on silver electrode. Scale bar: 40 μm. (d) Optical profilometry of different passes printed silver line. (e) Effect of printing passes on inductance and resistance of the printed antenna. (f) Effect of printing passes on the resonant frequency and capacitance of the printed capacitive interdigitated electrodes connected to an inductor. (g) Effect of antenna turns on inductance and resistance. (h) Effect of antenna line width on inductance and resistance. (i) Mechanical stability test of printed antennas under bending and twisting. Error bars represented standard deviation.

As illustrated in Fig. 2a, the ink was atomized by ultrasound, carried by nitrogen gas and sprayed onto a flexible PDMS substrate. AgNPs ink was printed as the circuit, while PI ink was printed as the encapsulation. The printed AgNPs were sintered at 250°C, causing the nanoparticles to fuse and the nanopores to progressively close, forming a robust, conductive metallic network (Fig. S3). Scanning electron microscope (SEM) images (Fig. 2b) showed a spatial uniformity of 2.9% for the printed silver lines. Excellent consistency in both height and width was further demonstrated (Fig. S4), confirming the high uniformity and stability of aerosol jet printing. After encapsulation, the micron-sized PI layer could be observed by optical profilometer and SEM, which uniformly covered the silver lines and maintained the microstructural spacing between the lines (Fig. 2c).

To optimize the electrical properties of the printed circuit, we investigated the effect of printing parameters. As shown in Fig. 2d, increasing the number of printing passes resulted in thicker deposition. However, beyond eight passes, the thickness could not increase further, likely due to the viscosity of the ink. Accordingly, the sheet resistance of the printed silver film decreased with the number of passes and stabilized after eight passes (Fig. S5). Besides, the sheet resistance continued to decrease with sintering time during the first three minutes, which was chosen as the optimal sintering time for subsequent experiments.

The effect of the number of printing passes on electrical properties of the printed inductive antenna and capacitive interdigitated electrode was further assessed. The resistance of the printed antenna decreased with the printing passes, while the inductance remained almost unchanged (Fig. 2e), as the thickness of the planar inductor did not significantly affect inductance with a defined shape. The quality factor, determined by both resistance and inductance, peaked after eight passes (Fig. S6). The parasitic capacitance increased, shifting the resonant frequency to a lower value (Fig. S7). For the printed capacitive interdigitated electrodes, the geometry was shown in Fig. S8. The capacitance was measured by determining the resonance frequency after connecting them to a 0.51 μH inductor. Obvious changes in both resonant frequency and capacitance were observed between four and eight printing passes (Fig. 2f). The slight changes after eight passes may be related to subtle variations in the lateral profile of the interdigitated electrodes, which could affect their fringing-field coupling [47,48]. Overall, eight passes were chosen as the optimal printing parameter for both the antenna and interdigitated electrode.

Additionally, the shape parameters of the antenna were investigated to obtain a miniaturized and efficient inductor. As shown in Fig. 2g, inductance increased from 0.50 to 0.81 μH with the addition of antenna turns, while resistance increased from 5.81 to 27.60 Ω. The trade-off between inductance and resistance indicated that a four-turn configuration would offer an optimal balance of performance and size. In terms of line width, a wider line resulted in a decrease in resistance but simultaneously reduced inductance (Fig. 2h). Specifically, when the line width increased from 0.06 to 0.4 mm, the resistance dropped from 45.80 to 2.91 Ω, while the inductance decreased from 0.65 to 0.47 μH. Consequently, a line width of 0.12 mm was determined to be the most suitable for balancing performance and design constraints. Finally, the fully printed electronic interface demonstrated excellent flexibility and tensile properties, maintaining a stable resonant frequency after 100 cycles of twisting and bending (Fig. 2i). This mechanical stability could support conformal tissue attachment and enable reliable signal readout.

### Design and fabrication of bioresponsive hydrogel

**Figure 3.**
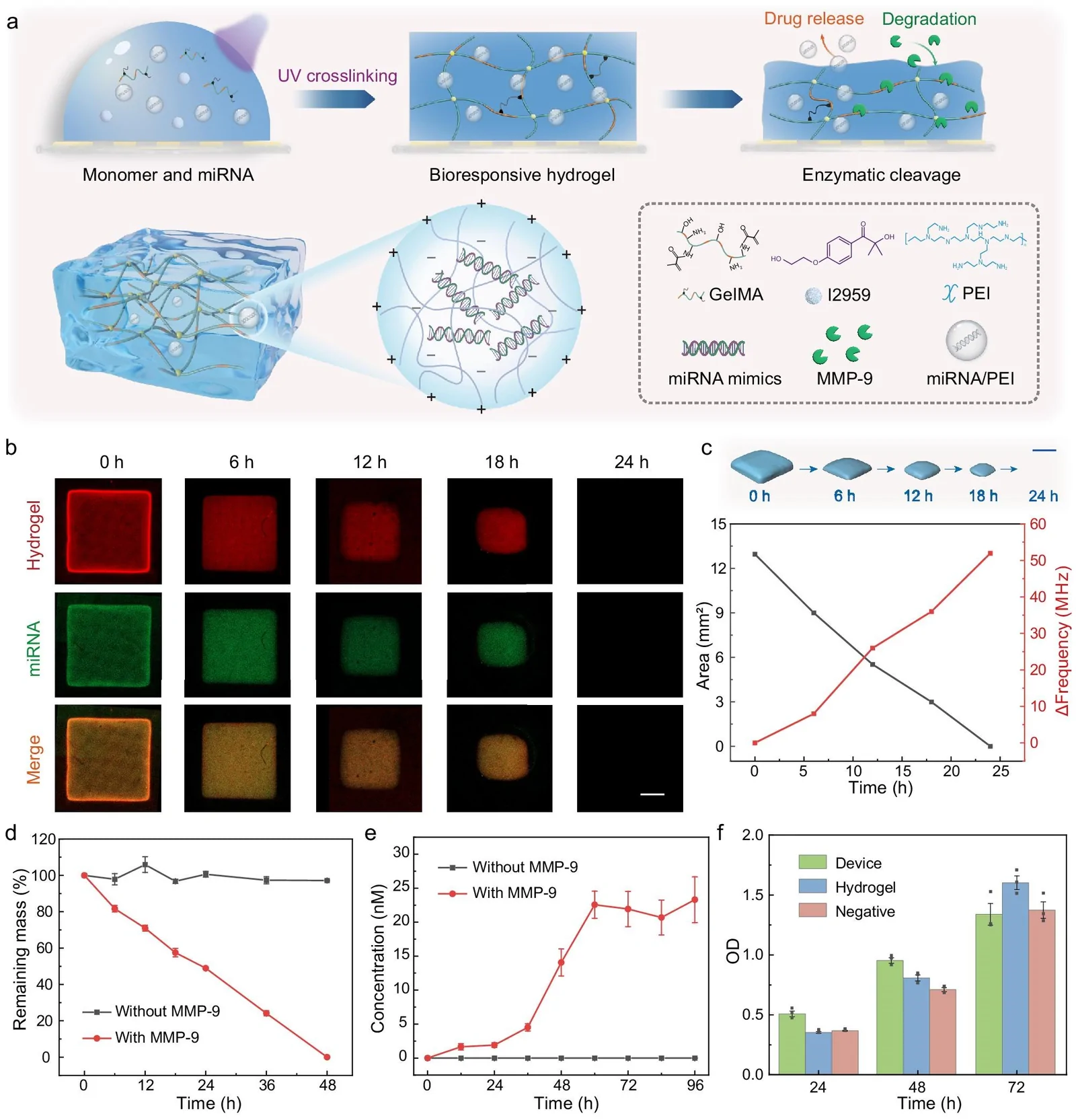
Bioresponsive hydrogel for sensing and drug delivery. (a) Schematic of the design and fabrication of the hydrogel-based WIRES. (b) CLSM images of the hydrogel incubated with MMP-9. Scale bar: 1 mm. (c) Area changes corresponding to the fluorescence images and numerical simulation of resonant frequency changes. The inset was three-dimensional topographic reconstruction of the hydrogel degradation process with different times. Scale bar: 2 mm. (d) Degradation profiles of the hydrogel incubated with MMP-9. (e) Amount of miRNAs released from the hydrogel incubated with MMP-9. (f) Quantitative analysis of cell metabolic activity after culture with hydrogel and WIRES device. Error bars represented standard error of the mean.

A bioresponsive hydrogel was designed as the biointerface for the WIRES device due to its biomechanical and structural similarity to biological tissues (Fig. 3a; Fig. S9 provided the synthesis details). To specifically respond to the periodontal inflammation marker MMP-9, modified gelatin molecules with bioactive peptide sequences were adopted as the backbone of the hydrogel, which was designed to degrade through the cleavage of glycine-containing sensitive motifs [49,50]. The gelatin molecules were functionalized with methacryloyl groups, enabling photopolymerization in the presence of a photoinitiator (Irgacure 2959, I2959). Ultraviolet light could induce free radical formation, initiating a cross-linking reaction to form a stable three-dimensional network, effectively enhancing the thermal stability and mechanical properties of the hydrogel compared to unmodified gelatin hydrogels [51]. Notably, miRNA was loaded in the hydrogel as the therapeutic drug for periodontal immunotherapy. To facilitate efficient delivery, the cationic polymer polyethyleneimine (PEI) was utilized to encapsulate the negatively charged miRNA molecules, forming miRNA nanoparticles through self-assembly due to the high density of amine groups in PEI. The miRNA nanoparticles exhibited a sphere-like morphology, with an average size of 312 nm (Fig. S10).

SEM images of the hydrogel showed a uniform and interconnected three-dimensional porous structure (Fig. S11), which facilitated substance exchange and signal transduction between the WIRES device and the periodontal microenvironment. To visualize hydrogel degradation and miRNA distribution in the presence of MMP-9, red fluorescein-labeled gelatin and green fluorescein-labeled miRNA were synthesized for hydrogel fabrication (Fig. 3b). Confocal laser scanning microscopy (CLSM) images showed that miRNAs were uniformly distributed in the hydrogel. When the hydrogel was degraded by MMP-9, miRNAs were released over time, while the remaining miRNAs were still well loaded in the hydrogel until complete degradation.

To demonstrate the role of the hydrogel in sensing signal transduction and drug delivery, volume and weight changes during the enzymatic degradation by MMP-9 were quantitatively evaluated. As shown in Fig. 3c, the cross-sectional area of the hydrogel was calculated from the CLSM image, which decreased along with its overall volume. The resonance responses of the WIRES with different hydrogels were verified through radio-frequency simulations. The frequency shift increased with the decreasing area, which enabled further quantitative analysis of the enzymatic cleavage of MMP-9. Similarly, the weight of the hydrogel also decreased with MMP-9 incubation, indicating the dissolution of substances (Fig. 3d). The cumulative release of miRNAs was quantified by reverse-transcription polymerase chain reaction (RT-PCR). As shown in Fig. 3e, miRNAs were gradually released from the hydrogel during MMP-9 incubation, while in the absence of MMP-9, miRNA release was minimal, which indicated that the hydrogel provided a good carrier for the controlled release of miRNAs.

Furthermore, the biocompatibility of the hydrogel and the WIRES device was evaluated due to their direct contact with intraoral tissues. The metabolic activity of human keratinocytes (HaCaT) cells co-cultured with the hydrogel and WIRES was analyzed by the Cell Counting Kit-8 assay. As shown in Fig. 3f, significant proliferation of HaCaT cells was observed across all groups over 72 h with no significant difference between groups. High cell viabilities were also observed in live/dead staining images of HaCaT cells (Fig. S12). Thus, the hydrogel and the WIRES device had excellent biocompatibility for safe and effective intraoral applications.

### Wireless, battery-free MMP-9 detection by radio-frequency biosensor

**Figure 4.**
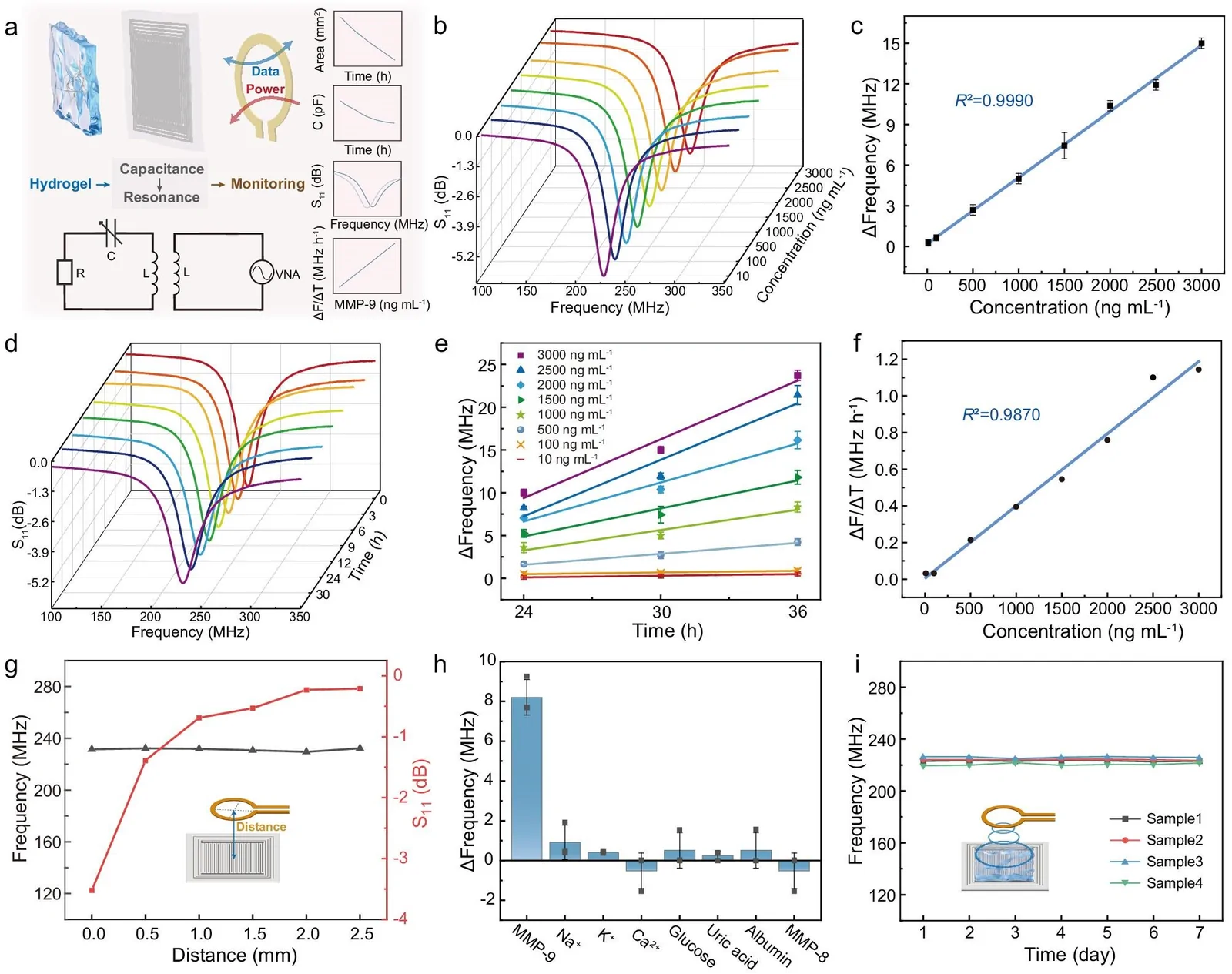
MMP-9 detection by radio-frequency biosensor. (a) Schematic of wireless MMP-9 detection by radio-frequency biosensor. (b) Resonant frequency response of WIRES incubated with different MMP-9 concentrations at 30 h. (c) Calibration plot of frequency shifts versus MMP-9 concentration at 30 h. (d) Resonant frequency response of WIRES incubated with 1000 ng/mL MMP-9 concentrations at different time points. (e) Frequency shift of WIRES incubated with different MMP-9 concentrations at different times. (f) Calibration plot of frequency shift rates from 24 to 36 h versus MMP-9 concentration. (g) Influence of reading distance between WIRES and the readout coil on the resonant frequency. (h) Selectivity test of the MMP-9 biosensor. (i) Stability test of the MMP-9 biosensor over time. Error bars represented standard deviation.

To achieve wireless and battery-free detection of periodontal inflammation, a radio-frequency biosensor consisting of the MMP-9-sensitive hydrogel and the flexible electronic interface was fabricated for transduction and transmission of biosignals. As shown in Fig. 4a, the simplified lumped element model of the sensor was represented by an RLC equivalent circuit, where L, C and R denoted coupled inductance, capacitance and resistance, respectively. The RLC circuit was inductively coupled to a readout coil (Fig. S13), and the resonant frequency could be read out remotely by portable or customized vector network analyzer. When MMP-9 entered the hydrogel network, the specific enzymatic cleavage of peptide sequences in the hydrogel backbone altered the capacitance, which resulted in a shift of the resonant frequency. The change in capacitance was primarily influenced by the covering area of the hydrogel, while its height had little effect on the frequency (Fig. S14).

The sensing performance of WIRES was investigated by analyzing its resonant frequency response to MMP-9 incubation. With the concentrations of MMP-9 increasing from 10 to 3000 ng/mL, the resonant frequency of WIRES increased accordingly (Fig. 4b). The frequency shifts after 30 h were fitted to a linear relationship with concentrations, which demonstrated excellent linearity of 0.9990 (Fig. 4c). Reported oral MMP-9 levels were relatively low in healthy individuals but increased with the severity and progression of periodontal inflammation, ranging from ∼100 ng/mL in early-stage periodontitis to much higher levels, even reaching the μg/mL range in severe cases [42,52,53]. The linear range of the sensor well covered the physiological MMP-9 concentrations in oral fluid, with a detection limit of 7.9 ng/mL calculated by 3σ/slope (where σ denoted the sensor noise). Linear fitting of concentrations and frequency shifts could be obtained at different time points (Fig. S15). Since the enzymatic cleavage process was time-dependent, MMP-9 could also be analyzed at various intervals (Fig. 4d). Specifically, larger frequency shifts were observed with time, and higher concentrations of MMP-9 could lead to faster shifts in frequency (Fig. 4e). During 24–36 h, the frequency shift rate was linearly proportional to the MMP-9 concentration (Fig. 4f). By performing repeated wireless readouts from the same patch at different time points, the accumulated frequency changes could be tracked to reflect the temporal profile of MMP-9 activity and indicate the inflammatory status. Accordingly, reliable concentration analysis relied on accumulation of the frequency response, and the required measurement time was currently on the order of hours depending on the MMP-9 concentration. Further improvement of hydrogel response kinetics and readout sensitivity may help shorten the measurement time.

To ensure wireless detection, the effective reading distance between WIRES and the readout coil was evaluated. As shown in Fig. 4g, the resonant frequency remained unaffected within a 2.5 mm reading distance, with only a decrease in the amplitude of the signals. The lower-band resonant frequencies were also less absorbed by biological tissues and exhibited better tissue penetration capabilities, enabling wireless signal transmission when the device was attached to the oral cavity or implanted in superficial tissues (Fig. S16). Additionally, the rotation angles of WIRES relative to the readout coil had negligible impact on the signal (Fig. S17), indicating that the radio-frequency detection was largely insensitive to relative orientation. The selectivity of the biosensor was evaluated with the potential interfering substances in saliva. As shown in Fig. 4h, ions (Na^+^, K^+^ and Ca^2+^), metabolites (glucose and uric acid) and proteins (albumin and MMP-8) all had little effect on MMP-9 detection, which was attributed to the specific recognition of peptide sequences in the hydrogel backbone by MMP-9. The sensor was also unaffected by varying pH values or buffer solutions (Fig. S18), and showed negligible changes at different temperatures (Fig. S19). Finally, the stability of the biosensor was investigated by continuous measurements in artificial saliva without MMP-9 at 37°C (Fig. 4i), indicating that the obtained signal remained stable for 7 days. The repeatability of the biosensor was tested by successive measurements over 12 cycles to ensure its reliability for long-term analysis (Fig. S20).

### Immunomodulation with bioresponsive delivered miRNA

**Figure 5.**
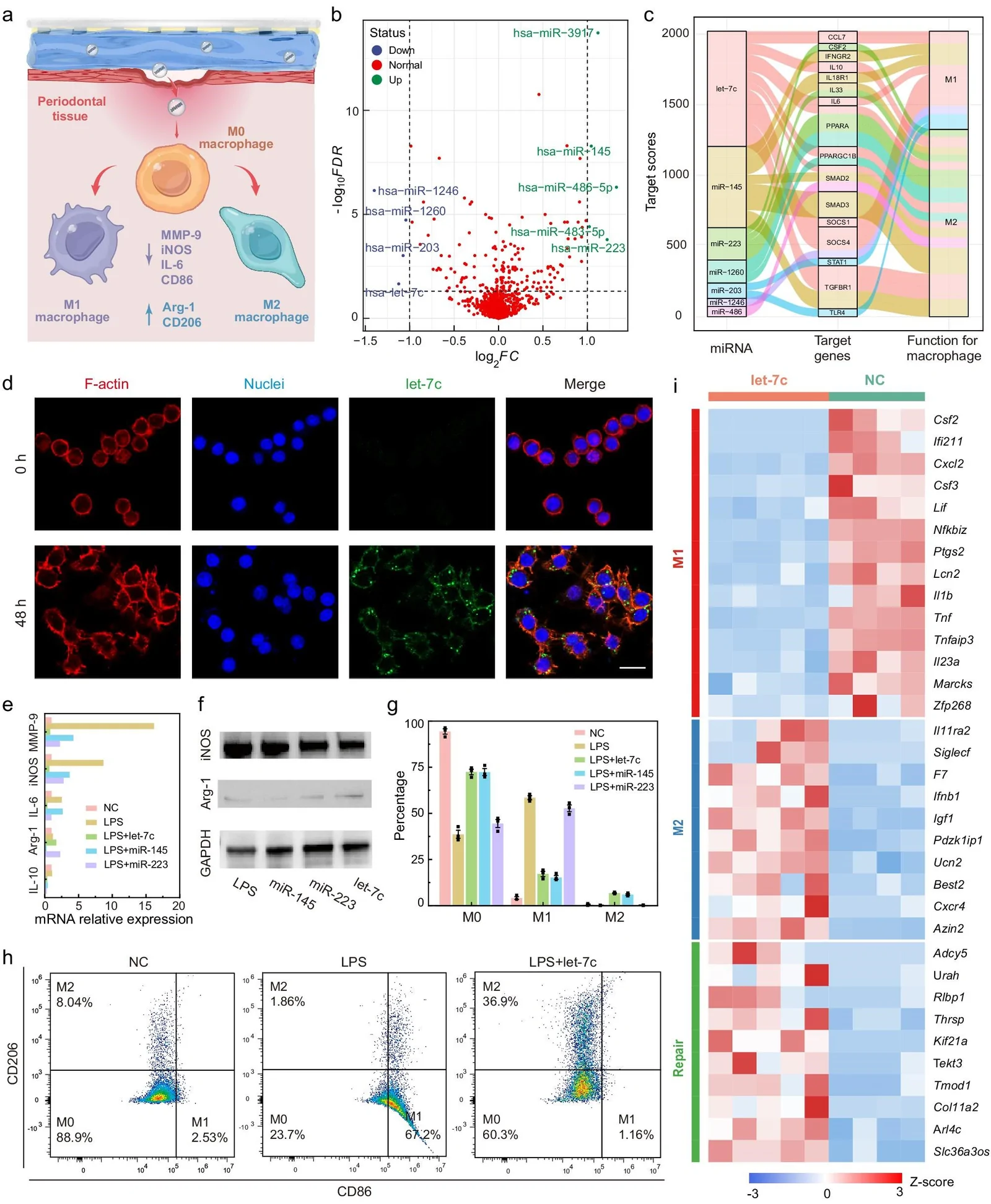
Bioresponsive delivery of immunomodulatory miRNA for macrophage reprogramming. (a) Schematic of controlled release of miRNA from the hydrogel-based WIRES. (b and c) Differential expression analysis of miRNAs in gingival tissues from patients with periodontitis and healthy individuals in the GEO database, including (b) volcano plot (log_2_FC >1 or <−1 and adjusted *P* < 0.05), and (c) alluvial diagram. (d) CLSM images showing cellular uptake of let-7c released from WIRES. The let-7c was labeled with 5-FAM. Cells were stained with rhodamine and 4',6-diamidino-2-phenylindole (DAPI). Scale bar: 20 μm. (e) Relative expression levels of representative polarization mRNAs in Pg-LPS-stimulated macrophages (RAW 264.7) treated with different miRNAs. (f) Expression levels of representative protein markers in Pg-LPS-stimulated RAW 264.7 macrophages treated with different miRNAs. (g) Flow cytometry analysis showing the impact of different miRNAs on the polarization of Pg-LPS-stimulated RAW 264.7 macrophages. (h) Flow cytometry plots illustrating the impact of let-7c on polarization of Pg-LPS-stimulated macrophages from mouse-derived macrophages. The *x*-axis represented fluorescence intensity of CD86 (M1), and the *y*-axis represented CD206 (M2). (i) Heatmap of DEGs associated with macrophage polarization in let-7c-treated RAW 264.7 macrophages compared with NCs. Expression values were shown as row *Z*-scores of FPKM (Fragments Per Kilobase of transcript per Million mapped reads). Error bars represented standard error of the mean.

Periodontitis involved an excessive inflammatory immune response that disrupted the periodontal microenvironment, where macrophages polarized into M1 pro-inflammatory and M2 anti-inflammatory phenotypes. As shown in Fig. 5a, during hydrogel degradation by MMP-9, miRNA nanoparticles were controllably released into the microenvironment, which were taken up by cells and participated in regulating gene expression and macrophage polarization. Specific miRNAs regulated macrophage polarization through various pathways, influencing the overall inflammatory response within the periodontal microenvironment.

To identify effective miRNAs for immunotherapy, differential expression analysis was performed on miRNAs from gingival tissues of periodontitis patients and healthy controls, using data from the Gene Expression Omnibus (GEO) database. As shown in Fig. 5b, a volcano plot was generated, where miRNAs with log_2_ fold change (log_2_FC) >1 or <−1 and an adjusted *P* < 0.05 were identified as differentially expressed. Nine miRNAs were selected based on these criteria. Further screening of biologically significant miRNAs in the MicroRNA Database predicted their potential for macrophage polarization. Twenty genes related to macrophage polarization were selected as target genes (Table S1). The heatmap revealed that let-7c-5p (hereafter referred to as let-7c) and six other miRNAs could regulate these target genes (Fig. S21). An alluvial diagram further showed that let-7c-5p, miR-145-5p and miR-223-3p had the highest matching scores with the target genes (Fig. 5c).

To evaluate the immunomodulatory effects of these miRNAs, the murine macrophage cell line RAW 264.7 was stimulated with *Porphyromonas gingivalis* lipopolysaccharides (Pg-LPS) and treated with different miRNAs. To verify the cellular uptake of the miRNA released from the WIRES, miRNAs were labeled with 5-carboxyfluorescein (5-FAM). As shown in Fig. 5d, since the miRNA was fully loaded in the hydrogel, there was no green fluorescence around the cells at 0 h. After 48 h, green fluorescence appeared both around and within the cells, which indicated that miRNAs released from the hydrogel had entered the cells. The relative expression of representative polarization genes was evaluated by RT-PCR. As shown in Fig. 5e, the mRNA expression levels of M1 pro-inflammatory markers (including MMP-9, inducible nitric oxide synthase (iNOS) and interleukin-6 (IL-6)) were downregulated in the miRNA-treated groups compared to the control and LPS-stimulated groups, with the group treated with let-7c showing the most pronounced effect. Protein expression levels were evaluated by western blot (Fig. 5f). The expression of the M1 polarization marker iNOS was significantly decreased by miR-223 and let-7c compared to the LPS-stimulated group, while the M2 polarization marker arginase-1 (Arg-1) was upregulated by let-7c.

Furthermore, flow cytometry was utilized to quantitatively assess the immunomodulatory effects of different miRNAs on RAW 264.7 macrophages (Fig. S22). As shown in Fig. 5g, compared to the LPS-stimulated group, the let-7c and miR-145 groups showed a significant decrease in the M1 macrophage population and an increase in the M2 macrophage population. Therefore, considering both the bioinformatic matching score and the comparative effects on gene expression, protein expression and cell polarization, let-7c was finally selected as the miRNA to be loaded into the WIRES for periodontal immunotherapy. Morphological differences between M0, polarized M1 and M2 macrophages after treatment with let-7c could also be clearly observed under bright-field microscopy (Fig. S23). The immunomodulatory effect of let-7c was then validated with the mouse bone marrow-derived macrophages. As shown in Fig. 5h, treatment with let-7c substantially decreased the M1 macrophage population and increased the M2 macrophage population, which demonstrated that it could effectively reprogram the macrophages into anti-inflammatory phenotypes. Similar immunomodulatory effects of let-7c were also observed in macrophages derived from human THP-1 monocytes using phorbol 12-myristate 13-acetate differentiation (Fig. S24).

To validate the regulatory role of let-7c in driving macrophage polarization and cellular remodeling, we conducted *in vitro* studies using murine macrophages (RAW 264.7). Briefly, cells stimulated by Pg-LPS were transfected with let-7c mimics or negative controls (NCs) to induce miRNA overexpression, followed by high-throughput RNA-sequencing (RNA-seq) to profile the transcriptomic landscape. High-throughput RNA-seq yielded an average of 58 million clean reads per sample with a Q30 quality score exceeding 95% and a genome mapping rate of over 91%, ensuring high data quality for subsequent analyses. Principal component analysis demonstrated distinct clustering between the let-7c mimic-treated group (*n* = 5) and the NC group (*n* = 4), indicating good biological reproducibility (Fig. S25). Differential expression analysis identified a total of 311 significant differentially expressed genes (DEGs) based on the criteria of |log_2_FC| > 1 and *P* < 0.05. Among these, 170 genes were significantly upregulated, and 141 genes were significantly downregulated by let-7c overexpression. As illustrated in Fig. 5i, functional grouping analysis of the DEGs unveiled a comprehensive transcriptional reprogramming induced by let-7c. Compared with the NC, let-7c significantly inhibited the M1 pro-inflammatory program (e.g. *Il1b, Tnf* and *Csf2*) while robustly activating the M2 anti-inflammatory and tissue repair program (e.g. *Igf1, Il11ra2* and *Siglecf*). Crucially, beyond immune modulation, we observed a prominent upregulation of genes involved in metabolic and structural remodeling (e.g. *Adcy5* and *Kif21a*), confirming that let-7c orchestrated a holistic phenotypic shift from M1 to M2, underpinned by essential metabolic and cytoskeletal adaptations. To define the functional impact of let-7c on macrophage polarization, we mapped all significant DEGs to the Kyoto Encyclopedia of Genes and Genomes database (Fig. S26). Functional profiling revealed a robust enrichment in pathways governing innate immune activation and cytokine signaling, which further supported the role of let-7c in promoting macrophage transition toward an anti-inflammatory and reparative phenotype.

### Validation of hydrogel-based WIRES for periodontitis sensing and therapy

**Figure 6.**
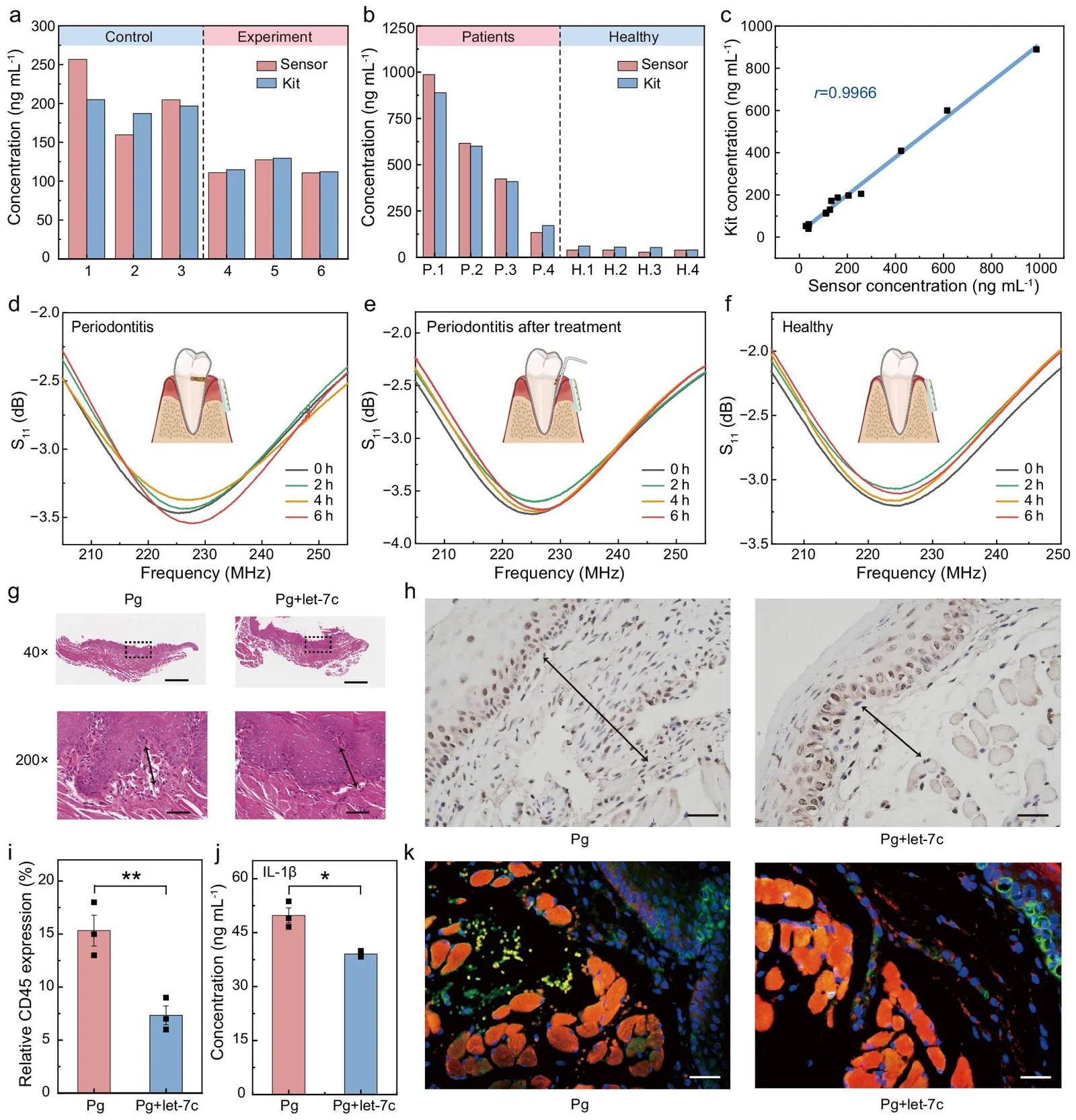
Functional validation of hydrogel-based WIRES. (a) Sensor performance in Pg-LPS stimulated macrophage samples with let-7c treatment, in comparison to the Active MMP-9 Fluorescent Assay. (b) Sensor performance in clinical samples from patients with periodontal diseases and healthy controls, in comparison to the Active MMP-9 Fluorescent Assay. (c) Correlation between sensor performance and the Active MMP-9 Fluorescent Assay. (d–f) Wireless monitoring of MMP-9 concentration in the oral cavity of a periodontitis patient before treatment (d), the patient after treatment (e) and a healthy volunteer (f). (g–k) *In vivo* therapeutic effects were evaluated in a murine periodontitis model. (g) H&E staining of gingival tissues from the Pg (*Porphyromonas gingivalis*-induced periodontitis) group and the Pg + let-7c (treated with let-7c) group. The arrows indicated the lamina propria layer to highlight the specific area for comparing inflammatory cell infiltration between the two groups. Scale bars: (40×) 250 μm; (200×) 50 μm. (h) CD45 IHC staining of gingival tissues. The arrows indicated the lamina propria layer to highlight the specific area for comparing CD45-positive immune cells. Scale bar: 50 μm. (i) Quantitative IHC analysis of relative CD45 expression in the gingiva. (j) ELISA analysis of IL-1β in the tissue. (k) mIF staining of gingival tissues, with DAPI in blue, CD206 in red and CD86 in green. Scale bar: 50 μm. Error bars represented standard error of the mean. Statistical significance is indicated by asterisks: *P < 0.05, **P < 0.01.

To evaluate the feasibility of the hydrogel-based WIRES for periodontal inflammation sensing, MMP-9 levels were analyzed in macrophage cultures and human saliva samples, and compared to a commercially available Active MMP-9 Fluorescent Assay. As shown in Fig. 6a, MMP-9 levels in macrophage culture samples after Pg-LPS stimulation and let-7c treatment were measured using WIRES. The MMP-9 levels in the treatment groups were significantly lower than those in the control groups (Fig. S27), indicating the immunomodulatory effect of let-7c on macrophages.

Saliva samples were collected from eight volunteers for MMP-9 detection, four of whom were patients with varying degrees of periodontal disease, and four were healthy controls (Table S2). The frequency shifts of the sensors were measured at different times, and the frequency shift rates were used to determine MMP-9 concentrations. As shown in Fig. 6b, WIRES could well discriminate MMP-9 levels in the saliva of healthy and diseased subjects, reflecting different oral inflammatory conditions. A good correlation was obtained between the sensor response and the commercially available fluorescent assay (Fig. 6c). The limit of agreement was also determined by a Bland–Altman plot. The mean detection difference was 3.6 ng/mL, and the difference was within 69.0 ng/mL with 95% confidence (Fig. S28).

To evaluate the applicability of the wireless sensing system in monitoring periodontal inflammation, the measurements were performed in a patient with periodontitis before and after treatment, as well as in a healthy volunteer (Fig. 6d–f). The resonant frequency increased over time in the patient with periodontitis (Fig. 6d), indicating the high activity of MMP-9. Following two weeks of supragingival and subgingival scaling, a reduced but still detectable signal shift was observed (Fig. 6e), suggesting partial suppression of the inflammatory state. In contrast, negligible variation in the resonant frequency signal was observed in the healthy subject (Fig. 6f), indicating low baseline levels of MMP-9. Based on the established calibration curve, the estimated MMP-9 concentrations in the oral cavity of the patient with periodontitis were 1132.0 ng/mL before treatment and 456.4 ng/mL after treatment, whereas the level in the healthy subject remained below the detection threshold. To further validate the readout, saliva samples collected during the same clinical testing session were analyzed using a commercial MMP-9 assay, showing results consistent with the WIRES-based estimation (Table S3). These results demonstrated that WIRES provided accurate detection of MMP-9, enabling *in situ* monitoring of periodontal inflammation.

In addition to *in situ* detection and wireless signal transmission, WIRES also facilitated periodontitis treatment through the modulation of the immune microenvironment. The therapeutic efficacy was evaluated in mouse models of periodontitis. After periodontal disease was induced by *Porphyromonas gingivalis*, let-7c (chemically modified agomir mimics) released from WIRES was injected into the gingiva of mice to assess its immunomodulatory effects at the tissue level. Periodontal tissues from the defect site were extracted for immune cell analysis. Fig. 6g showed the Hematoxylin and Eosin (H&E) staining images of the control and treatment groups, revealing a lower number of immune cells in the lamina propria of the treatment group.

To further demonstrate the immunomodulatory effects, the population and status of local immune cells were assessed by analysis of specific markers. CD45 was a pan-leukocyte marker that was used for identifying and quantifying immune cells. As shown in Fig. 6h, CD45-positive cells were stained brown using immunohistochemistry (IHC). A significantly higher proportion of CD45-positive cells was observed in the lamina propria of the control group compared to the treatment group (Fig. 6i). The level of pro-inflammatory cytokine interleukin-1β (IL-1β) in the tissue was quantified by enzyme-linked immunosorbent assay (ELISA). As shown in Fig. 6j, IL-1β was significantly reduced in the treatment group. Additionally, multi-immunofluorescence (mIF) staining was performed to demonstrate the polarization of gingival macrophages. DAPI was used to stain nuclei (blue), CD206 (an M2 polarization marker) was stained red and CD86 (an M1 polarization marker) was stained green (Fig. 6k). In the lamina propria between the epithelium and muscle tissue, more macrophages were observed in the control group, predominantly expressing CD86. In the treatment group, the number of macrophages labeled with both CD86 and CD206 was reduced, and the proportion of CD86-positive macrophages was also lower.

## DISCUSSION

The hydrogel-based WIRES developed in this study introduced a biointegrated platform that connected biological functions and digital information at the immune interface. By integrating radio-frequency circuits with enzymatically responsive hydrogels, WIRES enabled monitoring of inflammation activity and localized immunomodulation for periodontal disease. Unlike conventional rigid sensors or passive drug carriers, this platform coupled disease-specific biochemical recognition with wireless feedback and therapeutic intervention, providing a closed-loop strategy for precision care. With its tissue-mimicking, biocompatible and adhesive properties, the hydrogel layer served as a soft biointerface that allowed the flexible patch to conform to the moist gingival surface, providing an accessible option for patients with mild-to-moderate periodontal disease. For individuals with severe periodontitis, the miniaturized, flexible device could be painlessly implanted into the periodontal pocket following conventional subgingival scaling, allowing for seamless integration into routine dental care. WIRES was designed for temporary use and can be removed or replaced after treatment. For future development, transient and biodegradable materials could be incorporated to enable fully degradable bioelectronic devices [54,55].

A key advantage of WIRES lies in its ability to transduce inflammation-associated biochemical activity into measurable wireless signals, facilitating non-invasive detection of the local immune microenvironment. Here, MMP-9 was employed as a representative biomarker of periodontal inflammation. While conventional clinical assessments, including visual inspection and radiography, were limited by delayed detection and limited sensitivity to early-stage inflammation, the WIRES provided dynamic tracking of the disease activity. Compared with previously reported MMP-9 sensing methods (Table S4), the hydrogel-based sensor in this study was designed for *in situ* wireless monitoring rather than conventional laboratory-based biomarker detection. The peptide-functionalized hydrogel directly responded to MMP-9 enzymatic activity through peptide cleavage, which was closely associated with active inflammatory processes. In addition, the wireless readout supported *in situ* monitoring at the oral tissue interface, which was difficult to achieve with solution-based or benchtop detection methods. This modular sensing strategy could also be extended to detect other proteases or molecular targets by substituting recognition motifs (such as other peptide or aptamer sequences) within the hydrogel [33,56,57], making it a scalable platform for inflammatory disease diagnostics.

Beyond sensing, WIRES also offered inflammation-triggered delivery of immune-regulatory miRNAs, linking molecular diagnostics with on-demand, localized therapy. Compared with reported local drug delivery systems for periodontal therapy (Table S5), WIRES was distinguished by the integration of miRNA immunomodulation, MMP-responsive local release and wireless sensing of inflammatory activity. We identified let-7c as a crucial downregulated miRNA in periodontitis and loaded it into the hydrogel for bioresponsive release, enabling multifaceted, gene-level modulation of the local inflammatory microenvironment. The MMP-responsive hydrogel coupled drug release to disease-related enzymatic activity, rather than relying only on passive release. Importantly, hydrogel degradation not only delivered the drug but also generated a wireless signal, allowing feedback to guide timely drug replenishment or prompt further clinical intervention if necessary.

Besides, the hydrogel matrix was compatible with a variety of bioactive agents, such as nucleic acids, nanoparticles or even engineered bacteria [24,41,58], supporting future expansion toward regenerative and comprehensive interventions. From a translational perspective, localized miRNA delivery with hydrogel may reduce systemic exposure and potential off-target effects compared with systemic administration [59,60]. The non-viral, peptide-functionalized gelatin matrix also provided a biocompatible and biodegradable carrier for local periodontal delivery. While the current animal study validated the immunomodulatory effect of let-7c released from the hydrogel, future studies are needed to evaluate the patch for local therapeutic delivery in large-animal periodontal models.

In summary, we developed a bioresponsive hydrogel-based bioelectronic platform capable of wireless biosensing and on-demand immunomodulation. By integrating functionalized biomaterials with soft electronics, the system established a dynamic interface between the immune microenvironment and digital feedback networks, offering opportunities for adaptive management of chronic inflammation. This bioelectronic system held potential for broader applications to other inflammation-related diseases, such as wound healing, osteoarthritis or autoimmune disorders, providing a platform to explore dynamic interactions between electronic interfaces, bioactive hydrogels and the living biosystems.

## METHODS

Detailed methods are given in the Supplementary data.

## Supplementary Material

nwag409_Supplemental_File

## Data Availability

The RNA-seq datasets generated and analyzed during the current study are available from the corresponding author upon reasonable request. Raw sequencing data will be deposited in the Gene Expression Omnibus (GEO) database prior to publication.

## References

[1] Kinane DF, Stathopoulou PG, Papapanou PN. Periodontal diseases. Nat Rev Dis Primers 2017; 3: 17038. 10.1038/nrdp.2017.38 28805207

[2] Peres MA, Macpherson LMD, Weyant RJ et al. Oral diseases: a global public health challenge. Lancet 2019; 394: 249–60. 10.1016/S0140-6736(19)31146-8 31327369

[3] Chen MX, Zhong YJ, Dong QQ et al. Global, regional, and national burden of severe periodontitis, 1990–2019: an analysis of the global burden of disease study 2019. J Clin Periodontol 2021; 48: 1165–88. 10.1111/jcpe.13506 34101223

[4] Cekici A, Kantarci A, Hasturk H et al. Inflammatory and immune pathways in the pathogenesis of periodontal disease. Periodontol 2000 2014; 64: 57–80. 10.1111/prd.12002 24320956 PMC4500791

[5] Hajishengallis G . Periodontitis: from microbial immune subversion to systemic inflammation. Nat Rev Immunol 2015; 15: 30–44. 10.1038/nri3785 25534621 PMC4276050

[6] Slots J . Periodontitis: facts, fallacies and the future. Periodontol 2000 2017; 75: 7–23. 10.1111/prd.12221 28758294

[7] Kwon T, Lamster IB, Levin L. Current concepts in the management of periodontitis. Int Dent J 2021; 71: 462–76. 10.1111/idj.12630 34839889 PMC9275292

[8] Armitage GC, Xenoudi P. Post-treatment supportive care for the natural dentition and dental implants. Periodontol 2000 2016; 71: 164–84. 10.1111/prd.12122 27045436

[9] Hajishengallis G . Immunomicrobial pathogenesis of periodontitis: keystones, pathobionts, and host response. Trends Immunol 2014; 35: 3–11. 10.1016/j.it.2013.09.001 24269668 PMC3947349

[10] Loos BG, Van Dyke TE. The role of inflammation and genetics in periodontal disease. Periodontol 2000 2020; 83: 26–39. 10.1111/prd.12297 32385877 PMC7319430

[11] James P, Worthington HV, Parnell C et al. Chlorhexidine mouthrinse as an adjunctive treatment for gingival health. Cochrane Database Syst Rev 2017; 3: CD008676. 10.1002/14651858.CD008676.pub2 28362061 PMC6464488

[12] Feres M, Figueiredo LC, Soares GM et al. Systemic antibiotics in the treatment of periodontitis. Periodontol 2000 2015; 67: 131–86. 10.1111/prd.12075 25494600

[13] Yin Y, Yang S, Ai D et al. Rational design of bioactive hydrogels toward periodontal delivery: from pathophysiology to therapeutic applications. Adv Funct Mater 2023; 33: 2301062. 10.1002/adfm.202301062

[14] Wei X, Buse JB, Chen H et al. Towards intelligent and miniaturized drug delivery devices. Nature 2026; 651: 897–908. 10.1038/s41586-026-10221-3 41882132

[15] Wang Y, Du L, Wu H et al. An organ-conformal, kirigami-structured bioelectronic patch for precise intracellular delivery. Cell 2026; 189: 1086–107. 10.1016/j.cell.2025.12.021 41605209

[16] Timmerman MF, van der Weijden GA, van Steenbergen TJ et al. Evaluation of the long-term efficacy and safety of locally-applied minocycline in adult periodontitis patients. J Clin Periodontol 1996; 23: 707–16. 10.1111/j.1600-051X.1996.tb00599.x 8877655

[17] Rudhart A, Purucker P, Kage A et al. Local metronidazole application in maintenance patients. Clinical and microbiological evaluation. J Periodontol 1998; 69: 1148–54. 10.1902/jop.1998.69.10.1148 9802715

[18] Peng S, Fu H, Li R et al. A new direction in periodontitis treatment: biomaterial-mediated macrophage immunotherapy. J Nanobiotechnol 2024; 22: 359. 10.1186/s12951-024-02592-4 PMC11193307 38907216

[19] He XT, Li X, Xia Y et al. Building capacity for macrophage modulation and stem cell recruitment in high-stiffness hydrogels for complex periodontal regeneration: experimental studies in vitro and in rats. Acta Biomater 2019; 88: 162–80. 10.1016/j.actbio.2019.02.004 30735811

[20] Zhang X, Hasani-Sadrabadi MM, Zarubova J et al. Immunomodulatory microneedle patch for periodontal tissue regeneration. Matter 2022; 5: 666–82. 10.1016/j.matt.2021.11.017 35340559 PMC8942382

[21] Zarubova J, Hasani-Sadrabadi MM, Dashtimoghadam E et al. Engineered delivery of dental stem-cell-derived extracellular vesicles for periodontal tissue regeneration. Adv Healthc Mater 2022; 11: e2102593. 10.1002/adhm.202102593 35191610 PMC9233004

[22] Rupaimoole R, Slack FJ. MicroRNA therapeutics: towards a new era for the management of cancer and other diseases. Nat Rev Drug Discov 2017; 16: 203–22. 10.1038/nrd.2016.246 28209991

[23] Ma Y, Li S, Lin X et al. Bioinspired spatiotemporal management toward RNA therapies. ACS Nano 2023; 17: 24539–63. 10.1021/acsnano.3c08219 38091941

[24] Zhong R, Talebian S, Mendes BB et al. Hydrogels for RNA delivery. Nat Mater 2023; 22: 818–31. 10.1038/s41563-023-01472-w 36941391 PMC10330049

[25] Winkle M, El-Daly SM, Fabbri M et al. Noncoding RNA therapeutics—challenges and potential solutions. Nat Rev Drug Discov 2021; 20: 629–51. 10.1038/s41573-021-00219-z 34145432 PMC8212082

[26] Zhang J, Chen C, Fu H et al. MicroRNA-125a-loaded polymeric nanoparticles alleviate systemic lupus erythematosus by restoring effector/regulatory T cells balance. ACS Nano 2020; 14: 4414–29. 10.1021/acsnano.9b09998 32203665

[27] Ding N, Luo G, Li H et al. A cyclodextrin-based pH-responsive microRNA delivery platform targeting polarization of M1 to M2 macrophages for sepsis therapy. Adv Healthc Mater 2023; 12: e2301243. 10.1002/adhm.202301243 37463303

[28] Brasier N, Wang J, Gao W et al. Applied body-fluid analysis by wearable devices. Nature 2024; 636: 57–68. 10.1038/s41586-024-08249-4 39633192 PMC12007731

[29] Yuk H, Lu B, Zhao X. Hydrogel bioelectronics. Chem Soc Rev 2019; 48: 1642–67. 10.1039/C8CS00595H 30474663

[30] Hu L, Chee PL, Sugiarto S et al. Hydrogel-based flexible electronics. Adv Mater 2023; 35: e2205326. 10.1002/adma.202205326 36037508

[31] Liu N, Ma H, Li M et al. Electroconductive hydrogels for bioelectronics: challenges and opportunities. FlexMat 2024; 1: 269–301. 10.1002/flm2.31

[32] Lu Y, Aimetti AA, Langer R et al. Bioresponsive materials. Nat Rev Mater 2016; 2: 16075. 10.1038/natrevmats.2016.75

[33] Zhao F, Liu M, Guo H et al. Stimuli-responsive hydrogels based on protein/peptide and their sensing applications. Prog Mater Sci 2025; 148: 101355. 10.1016/j.pmatsci.2024.101355

[34] Wu M, Zhang Y, Liu Q et al. A smart hydrogel system for visual detection of glucose. Biosens Bioelectron 2019; 142: 111547. 10.1016/j.bios.2019.111547 31387025

[35] Han F, Xie X, Wang T et al. Wearable hydrogel-based epidermal sensor with thermal compatibility and long term stability for smart colorimetric multi-signals monitoring. Adv Healthc Mater 2023; 12: e2201730. 10.1002/adhm.202201730 36259562

[36] Tai YT, Wei CY, Ko FH. Hydrogel-based colorimetric power-saving sensors for on-site detection of chloride ions and glucose in sweat. Biosens Bioelectron 2024; 271: 117041. 10.1016/j.bios.2024.117041 39675232

[37] Mannoor MS, Tao H, Clayton JD et al. Graphene-based wireless bacteria detection on tooth enamel. Nat Commun 2012; 3: 763. 10.1038/ncomms1767 22453836

[38] Boutry CM, Beker L, Kaizawa Y et al. Biodegradable and flexible arterial-pulse sensor for the wireless monitoring of blood flow. Nat Biomed Eng 2019; 3: 47–57. 10.1038/s41551-018-0336-5 30932072

[39] Li S, Lu D, Li S et al. Bioresorbable, wireless, passive sensors for continuous pH measurements and early detection of gastric leakage. Sci Adv 2024; 10: eadj0268. 10.1126/sciadv.adj0268 38640247 PMC11029800

[40] Jiang Y, Trotsyuk AA, Niu S et al. Wireless, closed-loop, smart bandage with integrated sensors and stimulators for advanced wound care and accelerated healing. Nat Biotechnol 2023; 41: 652–62. 10.1038/s41587-022-01528-3 36424488

[41] Shi J, Kim S, Li P et al. Active biointegrated living electronics for managing inflammation. Science 2024; 384: 1023–30. 10.1126/science.adl1102 38815037

[42] Gursoy UK, Kononen E, Huumonen S et al. Salivary type I collagen degradation end-products and related matrix metalloproteinases in periodontitis. J Clin Periodontol 2013; 40: 18–25. 10.1111/jcpe.12020 23078613

[43] Sioustis IA, Martu MA, Aminov L et al. Salivary metalloproteinase-8 and metalloproteinase-9 evaluation in patients undergoing fixed orthodontic treatment before and after periodontal therapy. Int J Environ Res Public Health 2021; 18: 1583. 10.3390/ijerph18041583 33567492 PMC7915089

[44] Luchian I, Goriuc A, Sandu D et al. The role of matrix metalloproteinases (MMP-8, MMP-9, MMP-13) in periodontal and peri-implant pathological processes. Int J Mol Sci 2022; 23: 1806. 10.3390/ijms23031806 35163727 PMC8837018

[45] Khan Y, Thielens A, Muin S et al. A new frontier of printed electronics: flexible hybrid electronics. Adv Mater 2020; 32: e1905279. 10.1002/adma.201905279 31742812

[46] Yi H, Liu Y, Cao H et al. Material and process integrated innovations in aerosol jet printing: a review. Mater Today 2025; 91: 431–58. 10.1016/j.mattod.2025.11.001

[47] Igreja R, Dias CJ. Analytical evaluation of the interdigital electrodes capacitance for a multi-layered structure. Sens Actuators A 2004; 112: 291–301. 10.1016/j.sna.2004.01.040

[48] Esfandiari R, Maki DW, Siracusa M. Design of interdigitated capacitors and their application to gallium arsenide monolithic filters. IEEE Trans Microw Theory Tech 1983; 31: 57–64. 10.1109/TMTT.1983.1131429

[49] Nagase H, Fields GB. Human matrix metalloproteinase specificity studies using collagen sequence-based synthetic peptides. Biopolymers 1996; 40: 399–416. 10.1002/(SICI)1097-0282(1996)40:4<399::AID-BIP5>3.0.CO;2-R 8765610

[50] Van den Steen PE, Dubois B, Nelissen I et al. Biochemistry and molecular biology of gelatinase B or matrix metalloproteinase-9 (MMP-9). Crit Rev Biochem Mol Biol 2002; 37: 375–536. 10.1080/10409230290771546 12540195

[51] Kurian AG, Singh RK, Patel KD et al. Multifunctional GelMA platforms with nanomaterials for advanced tissue therapeutics. Bioact Mater 2022; 8: 267–95. 10.1016/j.bioactmat.2021.06.027 34541401 PMC8424393

[52] Isaza-Guzman DM, Arias-Osorio C, Martinez-Pabon MC et al. Salivary levels of matrix metalloproteinase (MMP)-9 and tissue inhibitor of matrix metalloproteinase (TIMP)-1: a pilot study about the relationship with periodontal status and MMP-9(-1562C/T) gene promoter polymorphism. Arch Oral Biol 2011; 56: 401–11. 10.1016/j.archoralbio.2010.10.021 21094936

[53] Koksal C, Yilmaz HE, Narin F et al. Salivary galectin-7, galectin-10, and MMP-9 levels in periodontally healthy, gingivitis, and periodontitis patients. J Periodontol 2026; 97: 907–18. 10.1002/jper.70015 41553873

[54] Lan Y, Li S, Guo H et al. Soft biodegradable implants for long-distance and wide-angle sensing. Nature 2026; 649: 366–74. 10.1038/s41586-025-09874-3 41501198

[55] Wu H, Wang Y, Li H et al. Accelerated intestinal wound healing via dual electrostimulation from a soft and biodegradable electronic bandage. Nat Electron 2024; 7: 299–312. 10.1038/s41928-024-01138-8

[56] Park CH, Thompson IAP, Newman SS et al. Real-time spatiotemporal measurement of extracellular signaling molecules using an aptamer switch-conjugated hydrogel matrix. Adv Mater 2024; 36: e2306704. 10.1002/adma.202306704 37947789

[57] Yu W, Gong E, Wang C et al. *In situ* implantable DNA hydrogel for diagnosis and therapy of postoperative rehemorrhage following intracerebral hemorrhage surgery. Sci Adv 2024; 10: eado3919. 10.1126/sciadv.ado3919 39141742 PMC11323940

[58] Nie C, Wang P, Wang J et al. Nanodrug engineered bacteria for tumor-targeted and synergistic photothermal immunotherapy. Sci China Mater 2026; 69: 2250–62. 10.1007/s40843-025-3640-3

[59] Hu S, Liang Y, Chen J et al. Mechanisms of hydrogel-based microRNA delivery systems and its application strategies in targeting inflammatory diseases. J Tissue Eng 2024; 15: 20417314241265897. 10.1177/20417314241265897 39092451 PMC11292707

[60] Mitchell MJ, Billingsley MM, Haley RM et al. Engineering precision nanoparticles for drug delivery. Nat Rev Drug Discov 2021; 20: 101–24. 10.1038/s41573-020-0090-8 33277608 PMC7717100

