# Supplementary material for: Inflammation-triggered sensing and miRNA immunotherapy with a wireless battery-free hydrogel bioelectronic patch
Source: Natl Sci Rev. 2026 Jul 2;13(16):nwag409. doi: 10.1093/nsr/nwag409 (PMC13499223; doi:10.1093/nsr/nwag409)
Supplement: Supplementary file 1 [file nwag409_supplemental_file.pdf]

Supplementary information

for

Inflammation-triggered sensing and miRNA immunotherapy with a wireless battery-free hydrogel bioelectronic patch

Zhenghan Shi [1,2,3,#], Yi Xu [4,#], Li-ang Zhou [1], Ye Liu [1], Feiyue Fang [1], Zijian An [1], Xin Li[1], Yanli Lu [1], Hao Wen [1], Lingkai Su [4,], Qingjun Liu [1,2,3,*]*

[1]Zhejiang Key Laboratory of Intelligent Sensing Technology and Advanced Medical Instrument, Key Laboratory for Biomedical Engineering of Ministry of Education, College of Biomedical Engineering & Instrument Science, Zhejiang University, Hangzhou 310027, China

[2]Taizhou Key Laboratory of Medical Devices and Advanced Materials, Zhejiang Key Laboratory of intelligent Medical Decision Support, Taizhou Institute of Zhejiang University, 318000, China

[3]Innovation Center for Smart Medical Technologies & Devices, Binjiang Institute of Zhejiang University, Hangzhou, 310053, China

[4]Stomatology Hospital, School of Stomatology, Zhejiang University School of Medicine, Zhejiang Provincial Clinical Research Center for Oral Diseases, Key Laboratory of Oral Biomedical Research of Zhejiang Province, Engineering Research Center of Oral Biomaterials and Devices of Zhejiang Province, Hangzhou, 310016, China

[#]These authors contributed equally: Zhenghan Shi, Yi Xu

* Corresponding Authors: Qingjun Liu, Lingkai Su

E-mail address: qjliu@zju.edu.cn (Q. Liu), lingkaisu@zju.edu.cn (L. Su)

Contents

Supplementary Note Methods
Supplementary Fig. 1 Fabrication of the hydrogel-based WIRES.
Supplementary Fig. 2 Optical image of the aerosol jet nozzle for printing the circuit.
Supplementary Fig. 3 Characterization of aerosol jet printed silver nanoparticle inks with different sintering time.
Supplementary Fig. 4 Partial optical profilometry of aerosol jet printed interdigital electrode.
Supplementary Fig. 5 Sheet resistance of aerosol jet printed silver nanoparticle inks with different printed passes and sintering time.
Supplementary Fig. 6 Quality factor of the printed inductor with different printed passes.
Supplementary Fig. 7 Return loss of the printed inductor with different printed passes.
Supplementary Fig. 8 Design parameters for the printed interdigitated electrode.
Supplementary Fig. 9 Schematic of synthesis and fabrication of the hydrogel.
Supplementary Fig. 10 Size distribution of miRNA nanoparticles.
Supplementary Fig. 11 SEM image of the bioresponsive hydrogel.
Supplementary Fig. 12 Representative live/dead fluorescent images of human keratinocytes (HaCaT) cells after 24 h and 72 h coincubation with the hydrogel and the WIRES.
Supplementary Fig. 13 Geometry and design parameters of the readout coil.
Supplementary Fig. 14 Influence of different heights of hydrogels with the same area.
Supplementary Fig. 15 Calibration plot of frequency shifts versus MMP-9 concentration at 24 h and 36 h.
Supplementary Fig. 16 Influence of the tissue between the WIRES and the readout coil.
Supplementary Fig. 17 Influence of rotation angles of the WIRES relative to the readout coil on the signal.
Supplementary Fig. 18 Resonant responses of MMP-9 biosensors to different pH and different buffers.
Supplementary Fig. 19 Resonant responses of MMP-9 biosensors in different temperatures.
Supplementary Fig. 20 Repeatability tests of the MMP-9 biosensor.
Supplementary Fig. 21 Heatmap for differential expression analysis of miRNAs in gingival tissues of patients with periodontitis and healthy individuals in the Gene Expression Omnibus (GEO) database.
Supplementary Fig. 22 Flow cytometry plots of the impact of different miRNAs on the polarization of *Porphyromonas gingivalis* lipopolysaccharides (Pg-LPS) stimulated macrophages (RAW 264.7), including negative control, LPS stimulated, let-7c, miR-145, and miR-223.
Supplementary Fig. 23 Representative microscopic images of macrophage polarization after different treatments, including M0, M1 and M2.

Supplementary Fig. 24 Flow cytometry analysis illustrating the impact of let-7c on the polarization of human macrophages.

Supplementary Fig. 25 Principal Component Analysis (PCA) of RNA-seq samples.

Supplementary Fig. 26 KEGG pathway enrichment analysis of differentially expressed genes (DEGs) in let-7c-5p overexpressing macrophages.

Supplementary Fig. 27 MMP-9 concentrations in macrophage culture samples after Pg-LPS stimulation and let-7c treatment measured by the WIRES.

Supplementary Fig. 28 Bland-Altman plot showing the agreement between the MMP-9 biosensor and commercially available Active MMP-9 Fluorescent Assay.

Supplementary Table 1 Genes associated with macrophage maturation and differentiation.

Supplementary Table 2 Periodontal status of volunteers diagnosed by the dentist and corresponding salivary MMP-9 concentrations detected by the WIRES.

Supplementary Table 3 Validation of in situ MMP-9 measurements using a commercial MMP-9 assay.

Supplementary Table 4 Comparison of MMP-9 sensing method for periodontal detection and this work.

Supplementary Table 5 Comparison of currently available local drug delivery systems for periodontitis treatment to our work.

Supplementary Table 6 Sequences of miRNA mimics and agomirs.

Supplementary Table 7 Primers used for RT-PCR.

Methods

Fabrication of all-printed flexible electronic interface

The flexible electronic interface was fabricated by the Aerosol Jet System Printer (Aerosol Jet 200, Optomec). The 200 μm PDMS film was adopted as the substrate, which was hydrophilically modified by oxygen plasma activation (TONSON, 80 W, 30 s). The inductive antenna and capacitive interdigitated electrode were printed with silver nanoparticle ink (JS-A426, NovaCentrix). PI ink (PI-AJ, UT Dots) was printed to cover the circuit as the encapsulation and dielectric layer. Both inks were printed using the 150 μm diameter nozzle. The aerosol flow rate was 20 sccm (standard cubic centimeter per minute), the sheath gas (N_2) flow rate was 30 sccm, and the print head moved at a speed of 2 mm/s.

The surface morphology of the electronic interface was characterized by SEM (Gemini 300, ZEISS) and optical profilometer (NT9100, Veeco). The sheet resistance of the printed silver nanoparticle ink was measured by four-point probe meter (HPS2523, HELPASS). The LCR meter (IM 3536, HIOKI) was used to test the capacitance and resistance. The Vector Network Analyzer (VNA300, MTINST) was used to characterize the inductance. The ImageJ software was used to analyze the width and consistency of the printed silver lines.

Fabrication of hydrogel-based bioelectronics

The bioresponsive hydrogels was prepared by photoinduced gelation of the modified gelatin molecules in an aqueous medium. The gelatin with a 30% degree of methacryloyl substitution was customized by Cure Gel Co., Ltd. In brief, 50 mg of lyophilized modified gelatin powder was added to 500 μL of 2% (w/v) Irgacure 2959 photoinitiator solution dissolved in phosphate buffer saline (PBS, pH=7.4), and the mixture was heated in a 37°C water bath until fully dissolved. 200 μL of 20 μg/mL PEI (Mw 25000, branched, Sigma-Aldrich) was slowly added to 200 μL of 1 μM miRNA, and the mixture was agitated for 1 minute and left to stand for 30 minutes to form miRNA nanoparticles. The above solutions were mixed in a 1:1 ratio to achieve a final concentration of 5% (w/v) gelatin and 0.25 μM miRNA.

The interdigitated electrode was then treated with plasma activation (TONSON, 80 W, 30 s), and a silicone mold (4 mm × 4 mm × 1.5 mm) was placed on the electrode. Subsequently, 24 μL of the prepolymer solution was added into the mold, followed by UV irradiation (365 nm, 8 W) for 20 minutes to form the hydrogel on the electrode in situ.

The particle size of miRNA nanoparticles was measured by nanoparticle size analyzer (Zetasizer Nano, Malvern). The hydrogels were freeze-dried and observed by SEM (Gemini 300, ZEISS). The fluorescein-labeled gelatin and miRNA were observed by confocal laser scanning microscopy (LSM980, ZEISS), which was used for 3D reconstruction of the hydrogels. The same laser power, detector gain, pinhole size, and optical settings were used for all confocal images. Imaris software was used to analyze the 3D model of the hydrogel images.

Numerical simulation of the electronic interface

Finite element analysis (FEA) was carried out on CST Studio Suite to verify the resonant frequencies of the electronic interface with hydrogel of different sizes. The core parameter that affects the resonant frequency was the contact area between the hydrogel and the interdigital electrode. High frequency mesh was used in the solution. Discrete ports and boundary conditions were as follows: (i) port type: S-Parameter, (ii) Impedance: 50 Ω, (iii) boundary type: open space. Solver was set as frequency domain, with minimum frequency 1 MHz and maximum frequency 2000 MHz. Relevant materials were consistent with the experimental conditions.

Activation of MMP-9.

Recombinant human MMP-9 protein was purchased from Sino Biological. The pro-MMP-9 (2 μg/mL) stock solution was dissolved in 2x TCNB buffer solution (400 mM NaCl, 100 mM Tris-HCl, 10 mM $CaCl_2$, 2 μM $ZnCl_2$ at pH=7). An APMA (p-amino phenylmercuric acetate, Sigma-Aldrich) solution was prepared for pro-MMP-9 activation by dissolving the 1M stock solution of APMA in DMSO and diluting it to 2 mM with deionized water. Pro-MMP-9 and APMA were mixed in a 1:1 (v/v) ratio, and the solution was incubated for 2 hours at 37°C to realize activation. For the experiments involving cells, pro-MMP-9 was also activated with trypsin. A 2 μg/mL solution of trypsin (Sigma-Aldrich) was freshly prepared in distilled water. Pro-MMP-9 (2 μg/mL) and trypsin solutions were mixed in a 1:1 (v/v) ratio and incubated for 45 minutes at 37°C. Then, 4 mg/mL soybean trypsin inhibitor (Aladdin) was added to the mixture and incubated for an additional 20 minutes at 37°C to inactivate the trypsin.

Characterization of sensing performances

The sensing performance of WIRES, including sensitivity, selectivity, stability, and repeatability, were characterized with a portable vector network analyzer (VNA3000). The circular read coil (copper, 1 oz) was fabricated on PCB (FR-4, 1.6 mm thickness), with a coil width of 5 mm. The return loss was measured from 1 to 1000 MHz in reflection mode. For each measurement, the initial resonance frequency before MMP-9 incubation was used as the baseline to calculate the frequency shift (ΔF). The resonant response is defined as:

$$\Delta \mathrm{F} = F - F_0$$

where F_0 and F were the resonant frequency of WIRES before and after incubation.

The pro-MMP-9 was first activated. The sensor was equilibrated in 1xTCNB buffer solution (200 mM NaCl, 50 mM Tris-HCl, 5 mM $CaCl_2$, 1 μM $ZnCl_2$ at pH=7) for 12 h before use. The MMP-9 solutions with concentrations ranging from 10 ng/mL to 3000 ng/mL were prepared for the sensitivity and linearity testing. The WIRES was incubated with different MMP-9 concentrations at 37 °C, and ΔF was measured continuously at different times. The potential interfering substances in the saliva including 15 mM sodium, 20 mM potassium, 1 mM calcium, 2 mM glucose, 400 μM uric acid, 30 μg/mL albumin, and 1000 ng/mL MMP-8

were prepared for the selectivity testing. The stability of WIRES was tested with TCNB buffer solution for 7 days.

Biocompatibility tests of the hydrogel-based device

The human keratinocytes (HaCaT) cells were seeded into glass bottom dishes (35 mm, Biosharp), and cultured in DMEM medium under 37°C and 5% CO_2. The hydrogel-based WIRES device and hydrogel degradation solution were placed into the culture dishes, and the cells were incubated for 24, 48, and 72 h, respectively. Cell metabolic activity was evaluated using the Cell Counting Kit-8 (CCK-8, Beyotime), and absorbance at 450 nm was measured with a microplate reader (SpectraMax M2e). Cell viability was assessed using the Calcein Acetoxymethyl Ester (AM)/ Propidium Iodide (PI) Live/Dead Cell Double Staining Kit (Solarbio), where live cells fluoresced green and dead cells red. Imaging was obtained with confocal laser scanning microscopy (LSM980, Zeiss).

Validation of the biosensor with patient

Volunteers were enrolled to validate the feasibility of the hydrogel-based WIRES for periodontal inflammation detection. The research protocol was approved by the Ethics Committee of Stomatology Hospital Affiliate at Zhejiang University. One group consisted of individuals with varying degrees of periodontal inflammation (diagnosed with periodontitis or gingivitis), while the other group were healthy volunteers. Unstimulated saliva samples from volunteers were collected and analyzed by WIRES. As a comparison, determination of MMP-9 activity in saliva samples was also quantified using a commercially available Active MMP-9 Fluorescent Assay (Fluorokine E, R&D Systems). In addition, on-body wireless sensing was performed in a patient with periodontitis (before and two weeks after periodontal therapy, including supragingival and subgingival scaling) and in a healthy subject. The signals were recorded every two hours over a 6-hour period using a portable vector network analyzer, and MMP-9 concentrations were estimated from the resonant frequency shift.

Synthesis of miRNA mimics and agomirs

MiR-145-5p mimics, miR-223-3p mimics, let-7c-5p mimics, FAM modified let-7c-5p, and let-7c-5p agomir were all obtained from Tsingke Biotechnology Co., Ltd. The specific sequences were listed in Supplementary Table 6.

RNA-seq

Total RNA of RAW 264.7 cells was extracted using the TRIzol reagent (Invitrogen) according to the manufacturer's protocol. RNA purity and quantification were evaluated using the NanoDrop 2000 spectrophotometer (Thermo Scientific). RNA integrity was assessed using the Agilent 2100 Bioanalyzer (Agilent Technologies). Then the libraries were constructed by Hangzhou Astrocyte Technology Co., Ltd. The libraries were sequenced on the Illumina NovaSeq X Plus and 150 bp paired-end reads were generated. The sequencing-generated FASTQ files were evaluated for read quality and mapping rate with FastQC (version 0.11.9).

Clean reads were aligned to the Mus_musculus_Ensembl_110 reference genome using Hisat2 (version 2.1.0). FPKM of each gene was calculated and the read counts of each gene were obtained by HTSeq-count (version 0.11.2). PCA analysis was performed using R (version 4.3.2) to evaluate the biological duplication of samples. Differential expression analysis was performed using the DESeq2(version 1.42).

Functional enrichment analysis of differentially expressed miRNAs in periodontitis

The clinical data and miRNA expression profiles from periodontitis patients and healthy individuals were obtained from the GEO database (GSE54710). Differential expression analysis was performed using R (4.3.2) with the DESeq2 package (1.42). The sequences of identified differentially expressed miRNAs were retrieved from Ensembl and subsequently used to predict mRNA targets via miRDB and starBase. Predicted target scores were obtained from miRDB database (Version 6.0, Prediction Tool MirTarget V4, miRNA Source miRBase 22). Based on these data, heatmaps and an alluvial diagram were generated to visualize miRNA-gene interactions using the ggalluvial package (0.9.1). Genes not targeted by any of the identified differentially expressed miRNAs were excluded from the diagrams.

To investigate the role of hsa-let-7c-5p in macrophage polarization, RNA-seq data from transfected macrophages were obtained. These datasets were merged, filtered to include only negative control and hsa-let-7c-5p samples, and processed using R (version 4.3.2) with the DESeq2 package (version 1.42) for differential expression analysis. Differentially expressed genes were identified based on an adjusted p-value threshold of <0.05 and absolute log2 fold change >1. Genes associated with macrophage polarization were selected from this list using a predefined set derived from relevant literature and visualizations. Normalized counts were transformed to Z-scores for heatmap generation via the pheatmap package (version 1.0.12). Pathway enrichment analysis of these genes was conducted using the clusterProfiler package.

Macrophage cell culture and transfection

The murine macrophage cell line RAW 264.7 was obtained from the American Type Culture Collection (ATCC) and maintained in Dulbecco's Modified Eagle's Medium (DMEM) supplemented with 10% fetal bovine serum (FBS) in a humidified atmosphere at 37°C with 5% CO_2. Cells were cultured in T-25 flasks and passaged using cold PBS solution upon reaching 70% confluence. The culture medium was changed every 2 days, and cell morphology was regularly monitored under an inverted phase-contrast microscope.

PEI was mixed with miRNA at the rate of 3:1 to transfect miRNA into the cells for 24h. RT-PCR was used for detection of miRNA in the cells. To detect whether let-7c transfected into cells, immunofluorescence staining was conducted in RAW 264.7. Let-7c-FAM was transfected into the cells using the above-mentioned method. Filamentous actin (F-actin) was labeled using Red-Rhodamine (Beyotime, C2207S), and the cell nuclei were stained with DAPI (Beyotime, P0131). Fluorescence images were captured using a confocal microscope (Zeiss LSM980).

Western blotting for detection of macrophage polarization markers

The GAPDH was the internal control protein and marker proteins of macrophage polarization were analyzed via Western blotting. The transfected cells were lysed in radioimmunoprecipitation assay (RIPA) buffer (Sigma-Aldrich) with Halt protease inhibitor (Thermo Fisher). The lysate was centrifuged at 12,000× g for 10 min at 4 °C. The total concentration of cellular proteins was determined using a bovine serum albumin (BSA) protein standard kit (Thermo Fisher). The protein samples were electrophoresed on 10% polyacrylamide gel electrophoresis (PAGE) gel (120 V, 1.5 h), and then transferred to nitrocellulose blotting membranes (Pall Corp) for 1.5 h at 300 mA. Protein blots were detected by SuperSignal West Pico (Thermo Fisher) with antibody GAPDH (Cell Signaling Technology, 2118, 1:5000), iNOS (Cell Signaling Technology, 13120, 1:1500), Arg-1 (Cell Signaling Technology, 93668, 1:1000) and secondary antibody goat anti-mouse IgG antibody (ThermoFisher, 31430) or goat anti-rabbit IgG antibody (ThermoFisher, 31460).

ELISA for quantification of cytokines and secreted proteins

IL-1β (JINHENGNUO, JHN80300) and MMP-9 (Abcam, ab246539 for human, ab253227 for mouse) ELISA kits were used to detect the levels of such cytokines and secreted proteins in the cell culture supernatant of RAW 264.7, 24h after the stimulation. Three biological replicates were analyzed. Absorbance was recorded at 450 nm on a microplate reader.

Flow cytometry for macrophage polarization evaluation

Cells were dissociated by cold PBS and then cells were filtered using a 100 μm cell screen and then a 40 μm cell screen. Cells were suspended in 100 μL of PBS containing 2% FBS at a concentration of 10^6 cells per tube. Nonspecific Fc receptors were first blocked using anti-CD16/32 (ThermoFisher, 14-0161-85), followed by incubation with targeted flow cytometry antibodies. For instance, cells were incubated with antibodies such as F4/80-FITC (ThermoFisher, 11-4801-82), CD86-BV421 (Biolegend, 105031), CD206-PE (BD Biosciences, 568273). Finally, the cells were analyzed using flow cytometry (Beckman; CytoFLEX).

RT-PCR for gene expression analysis

Total RNA was extracted from cell lines using the TRIzol Reagent (Invitrogen) and messenger RNA (mRNA) was reverse-transcribed into single-stranded complementary DNA using the PrimeScriptTM RT Reagent Kit (Takara, RR037B), miRNA using miRNA 1st strand cDNA synthesis kit (Accurate Biology, AG11717). Subsequent quantitative PCR amplification was performed with SYBR Green (ThermoFisher, A57156) on the Thermo Fisher QuantStudio 7 detection system (ThermoFisher). Primers used for RT-PCR were listed in Supplementary Table 7. β-Actin served as an internal standard control for messenger RNA (mRNA) detection and U6 served as an internal standard control for miRNA. Gene expression was quantified by the $2^{-\Delta\Delta Ct}$ method.

Culture of periodontal pathogenic bacteria

Porphyromonas gingivalis ATCC 33277 was cultivated in an anaerobic chamber using a modified brain heart infusion (BHI) medium (Solarbio, B8130) at an OD660 of 0.6-0.8.

Isolation and culture of mouse bone marrow-derived macrophages

Eight-week-old female C57BL/6 mice were sacrificed and immersed in 75% ethanol. Remaining tissue from the pelvic and femoral bones was carefully removed, and the bones were separated at the knee joint. The ends of the bones were then cut off, and a needle with bone marrow growth medium was used to expel the bone marrow from both ends of the bone. The marrow was collected by directing a jet of medium into a 10 cm cell culture dish. The following day, after centrifugation of the cell supernatant, the cell pellet was resuspended in complete culture medium containing 50 ng/mL macrophage colony-stimulating factor (M-CSF) for continued culture. The medium was changed every 3 days. During culture, some cells adhered to the dish, while others remained in suspension. The suspended cells were centrifuged and subsequently re-cultured in new dishes.

Therapeutic study in mouse periodontitis models

Ten-week-old female C57BL/6 mice were injected with *Porphyromonas gingivalis* (40 μL solution: 10^9 CFU/mL) every two days in gingiva and feed with 10^7 CFU/mL water for two weeks. For the experimental group, let-7c agomir released from WIRES was injected in the day 7 to 14 (twice a week, 200 nmol, 20 uL) in the gingiva, while control group injected the same volume physiological saline. The mice in all the groups were euthanized on day 14 according to the guidelines of Animal Care and Use Committee, Zhejiang University.

Histological analysis and immunostaining of gingiva

The gingiva samples were fixed in 4% paraformaldehyde fix solution (Servicebio) for 24 h at room temperature, dehydrated in graded ethanol and then embedded in wax. The tissue was cut into 5 μm sections using a microtome (Leica) and stained with HE. The sample section slides confirmed by HE were deparaffinized in xylene for 30 min and rehydrated using graded ethanol concentrations. Epitope retrieval was performed by a pressure cooker for 2.5 min. After being treated with hydrogen peroxidase blocker (Beyotime), sections were incubated at 4°C overnight with a CD45 antibody (Proteintech, 20103-1-AP, 1:500), CD86 antibody (Cell Signaling Technology, 91882, 1:150), CD206 (Cell Signaling Technology, 24595, 1:400) and followed with goat anti-mouse IgG antibody (ThermoFisher, 31430, 1:1500 for HE) or Goat anti-Rabbit secondary antibody Alexa Fluor 488 (ThermoFisher, A-11034) and Goat anti-Rabbit secondary antibody Alexa Fluor Plus 594 (ThermoFisher, A32740) for 30 min at room temperature after the washing step. After immunostaining, the IHC slides were counterstained with hematoxylin for 20 seconds to visualize the nuclei. Excess hematoxylin was removed by differentiating the slides in acid alcohol or tap water. DAB kits (Beyotime, P0203) were used according to the manufacturer's instructions. Immunofluorescence slides were stained with DAPI (Beyotime, P0131) before observation.

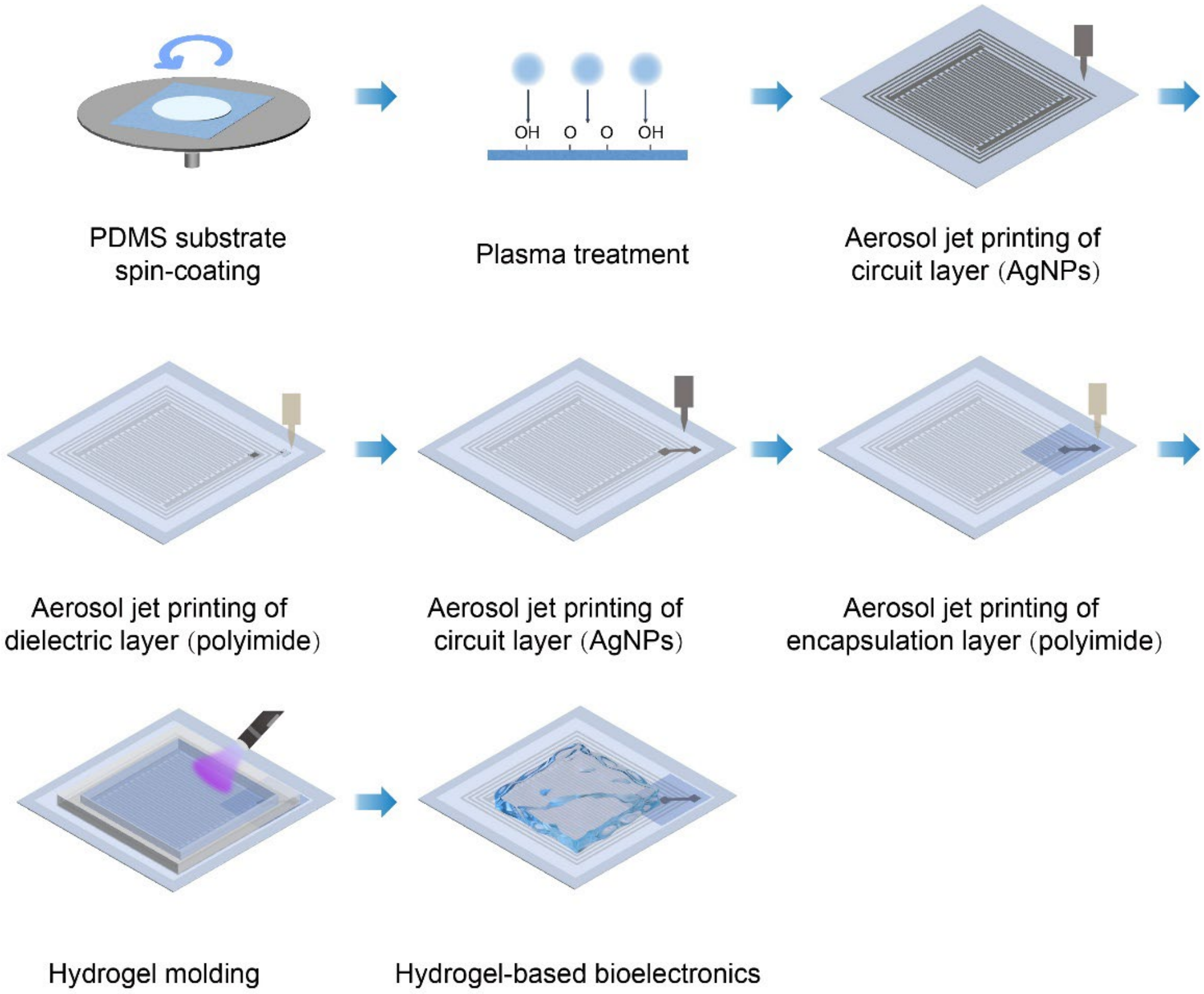

Supplementary Fig. 1 Fabrication of the hydrogel-based WIRES. Details of the fabrication process is illustrated in Methods.

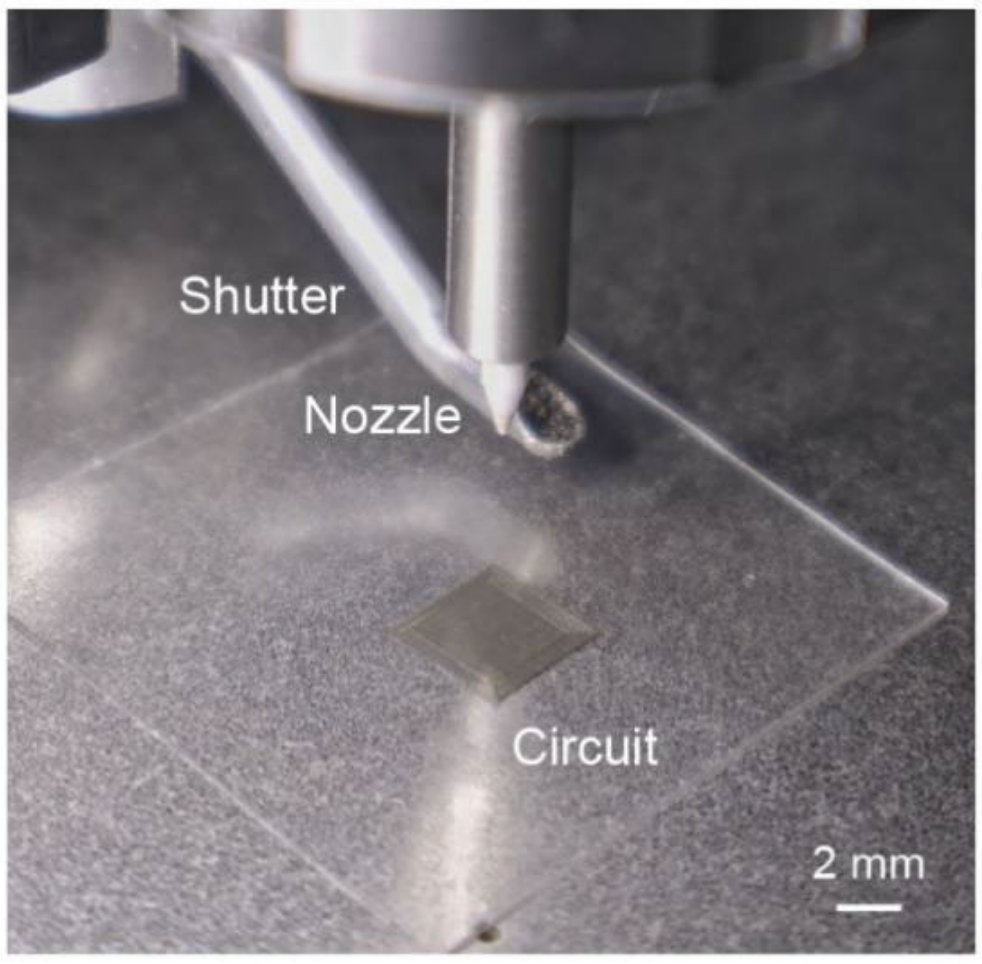

Supplementary Fig. 2 Optical image of the aerosol jet nozzle for printing the circuit.

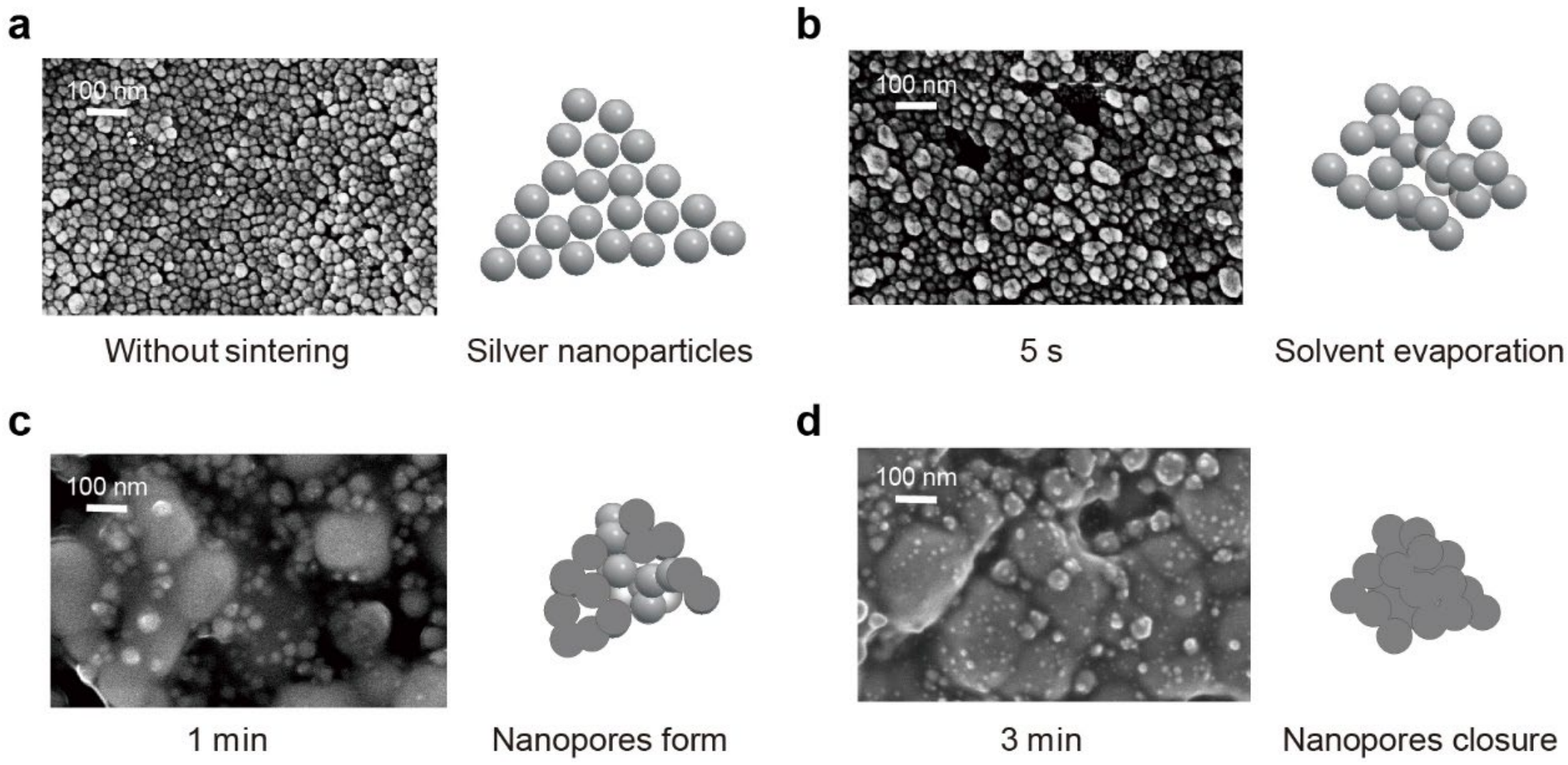

Supplementary Fig. 3 Characterization of aerosol jet printed silver nanoparticle inks with different sintering time, including without sintering (a), 5 s sintering (b), 1 min sintering (c), and 3 min sintering (d).

(a) Scanning electron microscopy (SEM) image of aerosol jet printed silver nanoparticle inks without sintering. The silver ink existed in the form of nanoparticles with a diameter of ~30 nm, at which time the electrical conductivity was poor. (b) SEM image of aerosol jet printed silver nanoparticle inks with 5 s sintering. After heating, the solvent evaporated quickly, and the gap between the silver nanoparticles decreased, but still did not form a continuous phase. (c) SEM image of aerosol jet printed silver nanoparticle inks with 1 min sintering. After 1 min sintering, the nanoparticles fused into solid phase and nanopores were formed. (d) SEM image of aerosol jet printed silver nanoparticle inks with 3 min sintering. After 3 min sintering, the nanopores were gradually closed which could greatly improve conductivity.

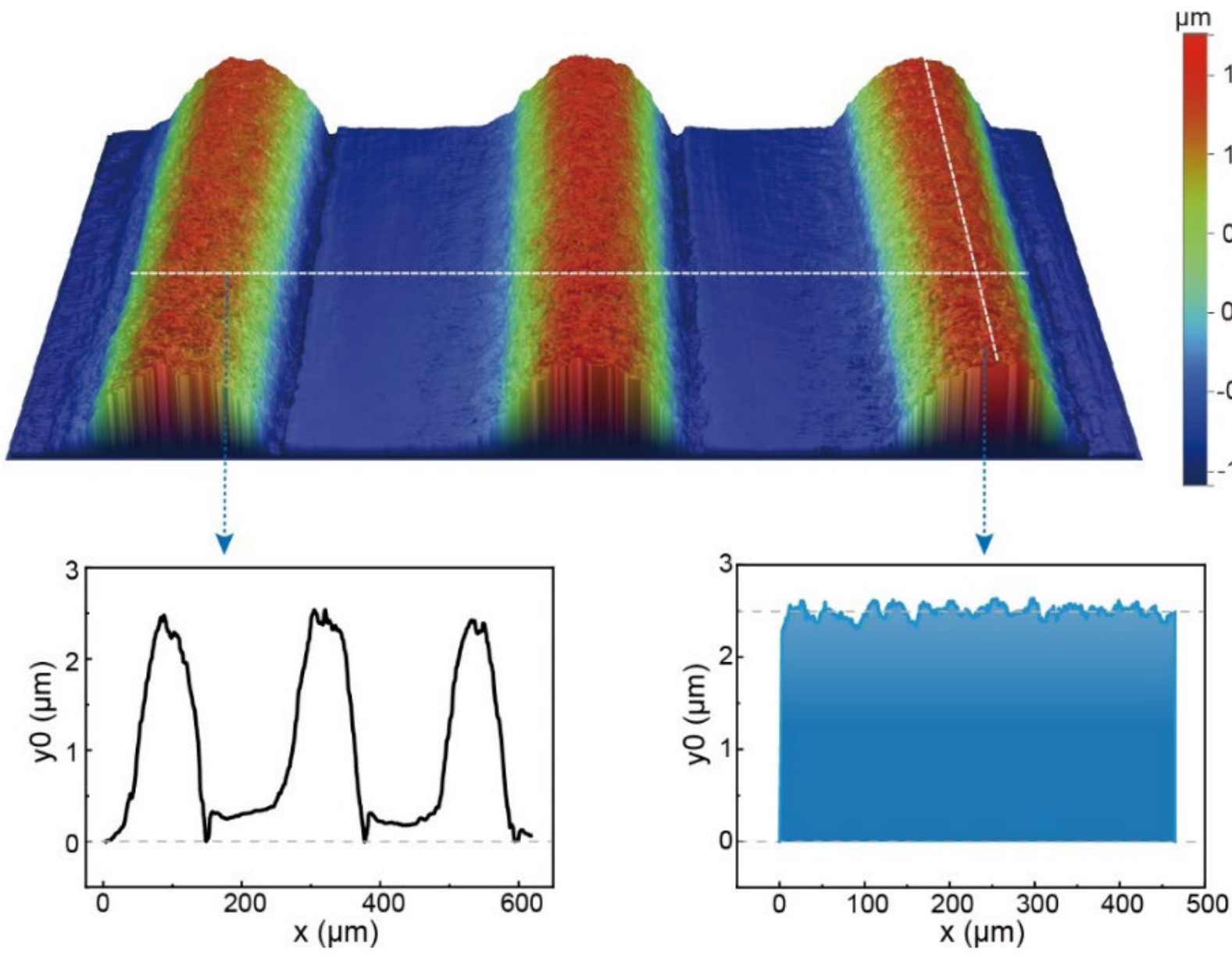

Supplementary Fig. 4 Partial optical profilometry of aerosol jet printed interdigital electrode.

The excellent consistency of the lines in both height and width was observed.

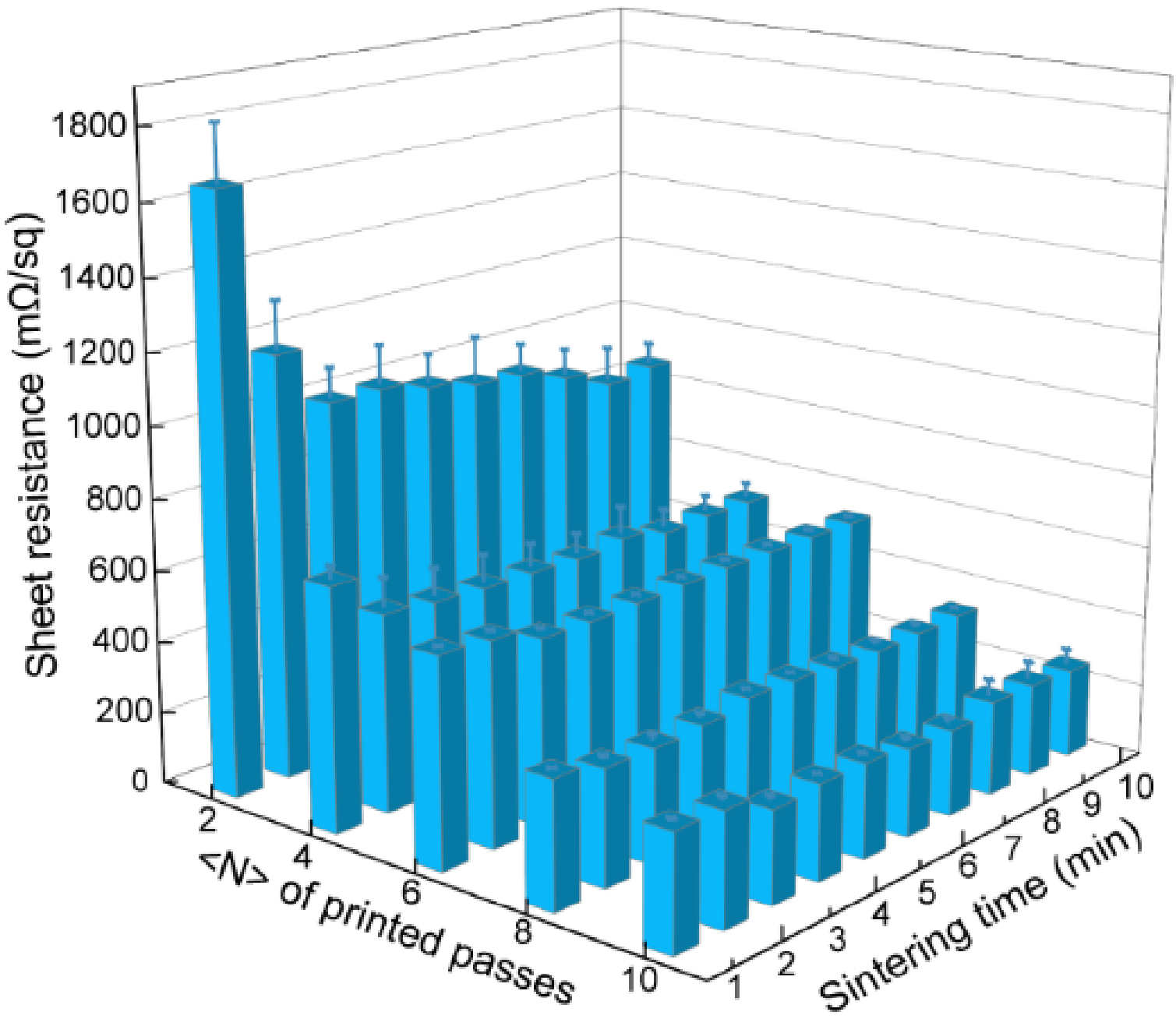

Supplementary Fig. 5 Sheet resistance of aerosol jet printed silver nanoparticle inks with different printed passes and sintering time.

The sheet resistance of inks decreased with the increase of printed passes, which stabilized after 8 passes. It also deceased with prolong sintering time in the beginning three minutes and then became stable.

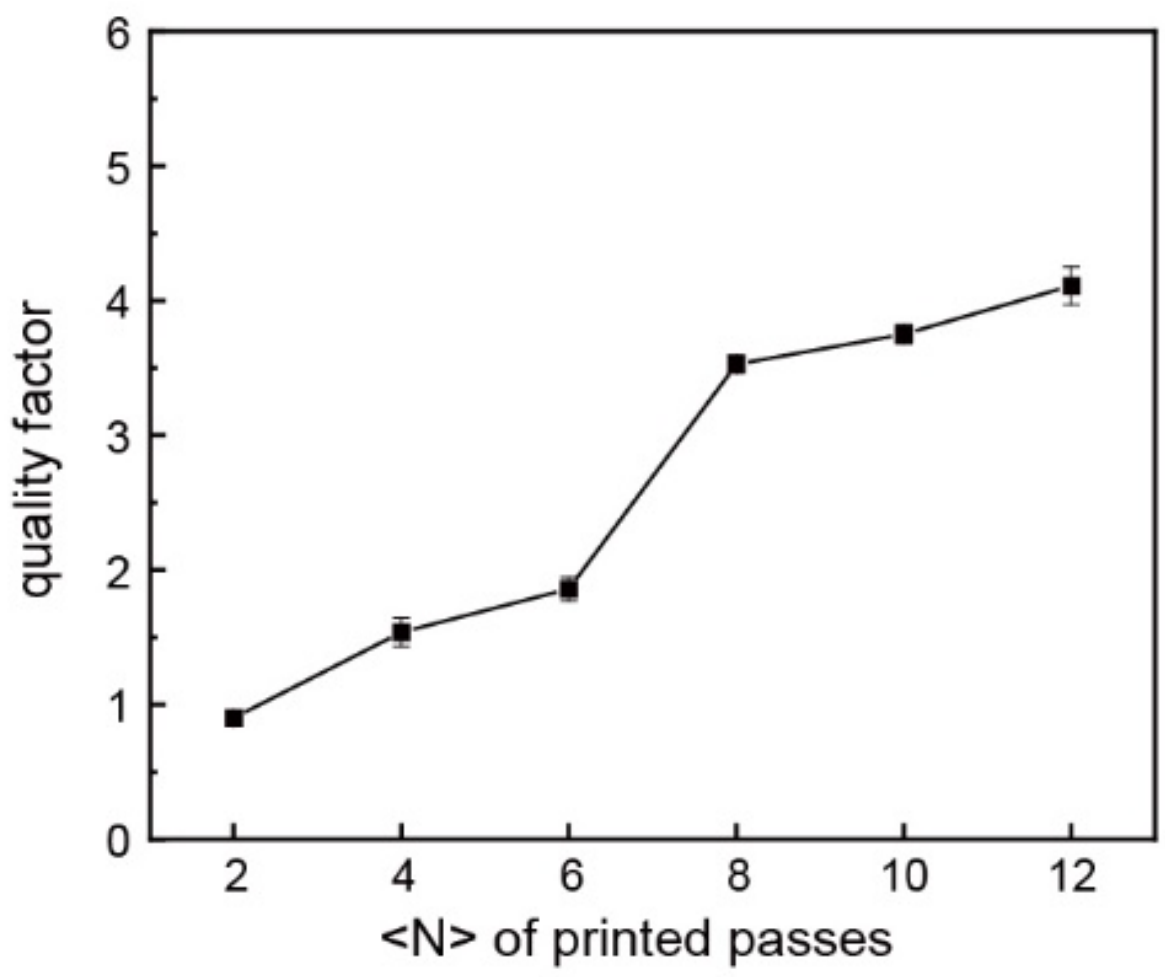

Supplementary Fig. 6 Quality factor of the printed inductor with different printed passes.

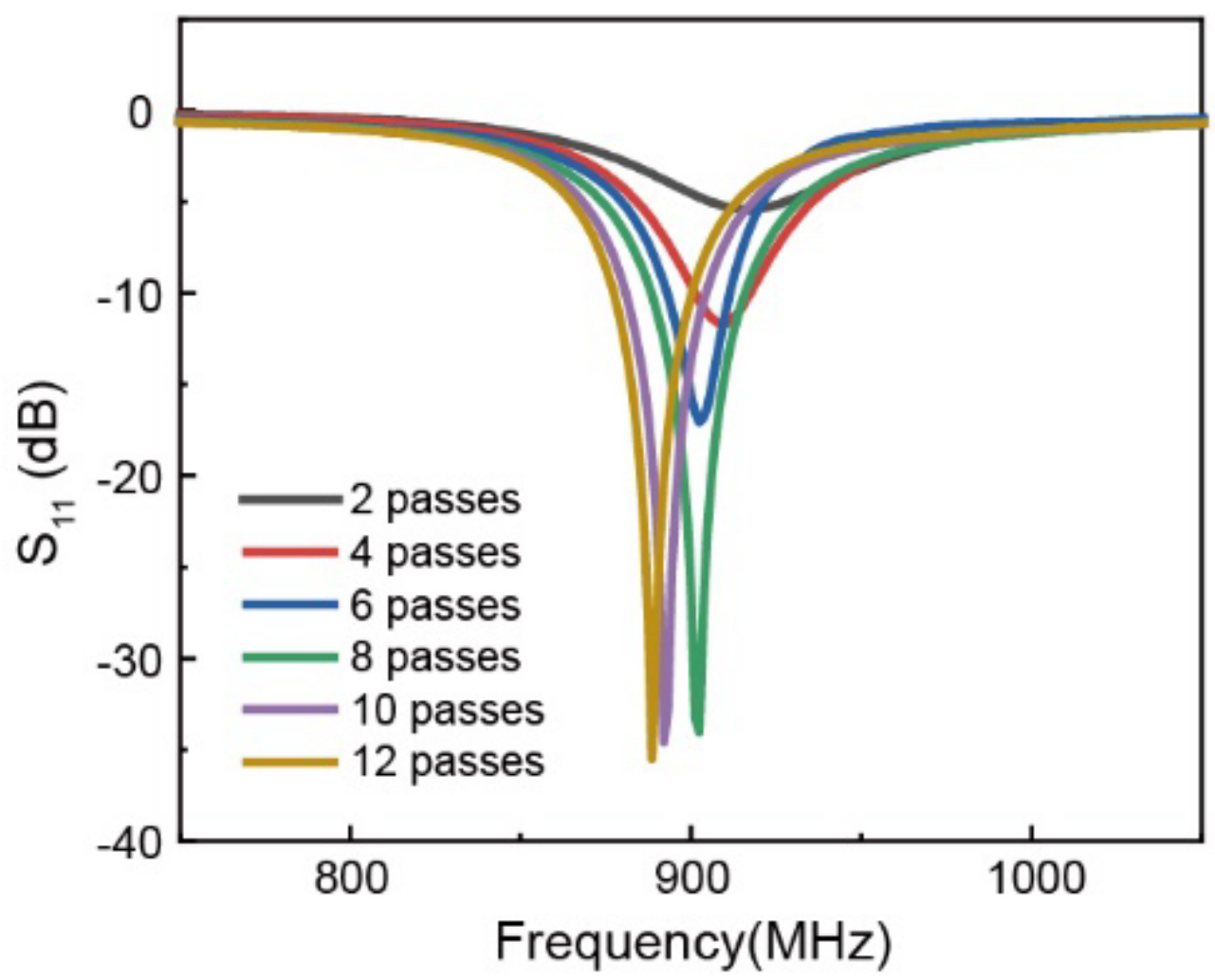

Supplementary Fig. 7 Return loss of the printed inductor with different printed passes.

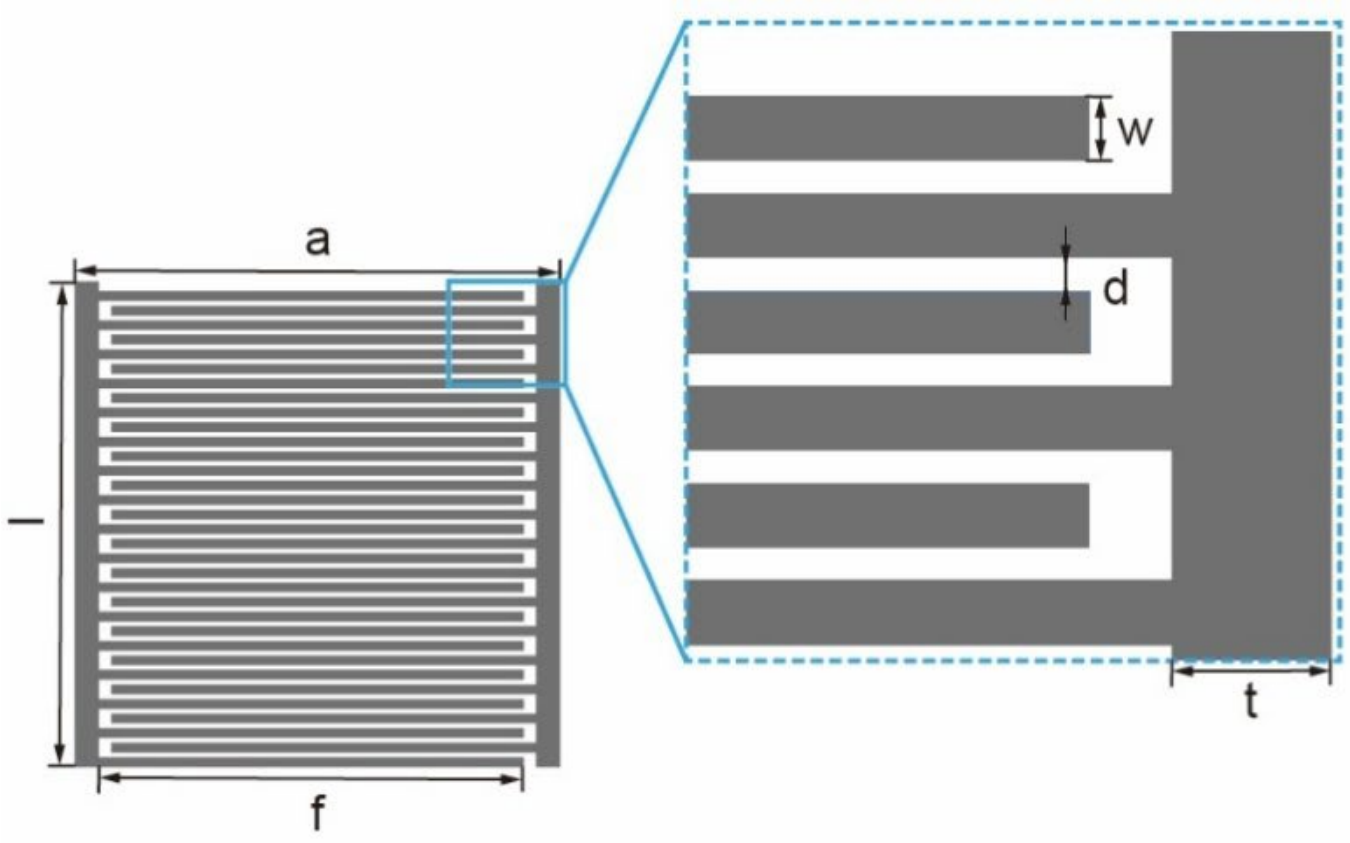

Symbol	Value
w	80 μm
d	40 μm
t	200 μm
f	3.5 mm
l	4 mm
a	4 mm

Supplementary Fig. 8 Design parameters for the printed interdigitated electrode.

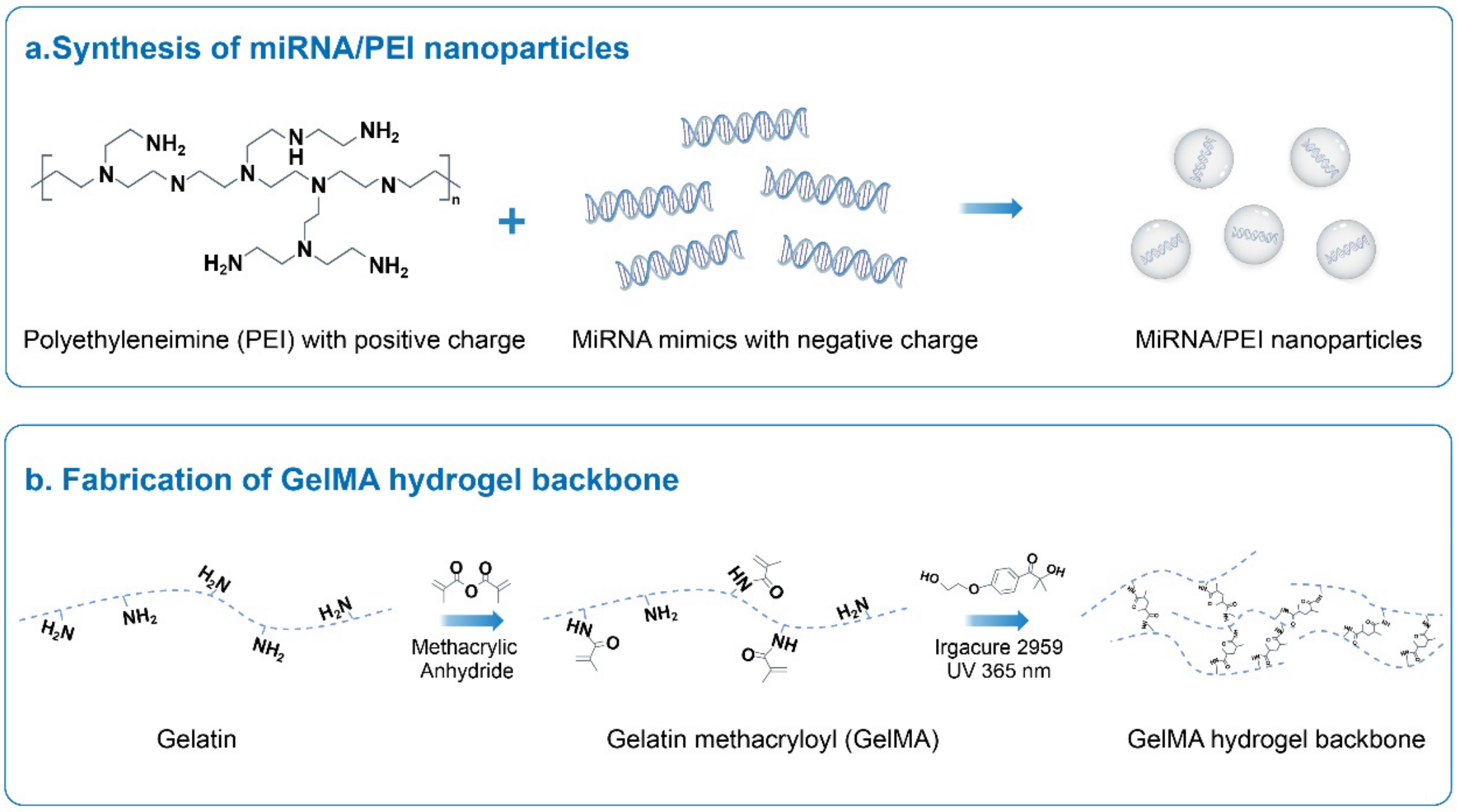

Supplementary Fig. 9 Schematic of synthesis and fabrication of the hydrogel. (a) Synthesis and fabrication of miRNA/PEI nanoparticles. (b) Fabrication of GelMA hydrogel backbone.

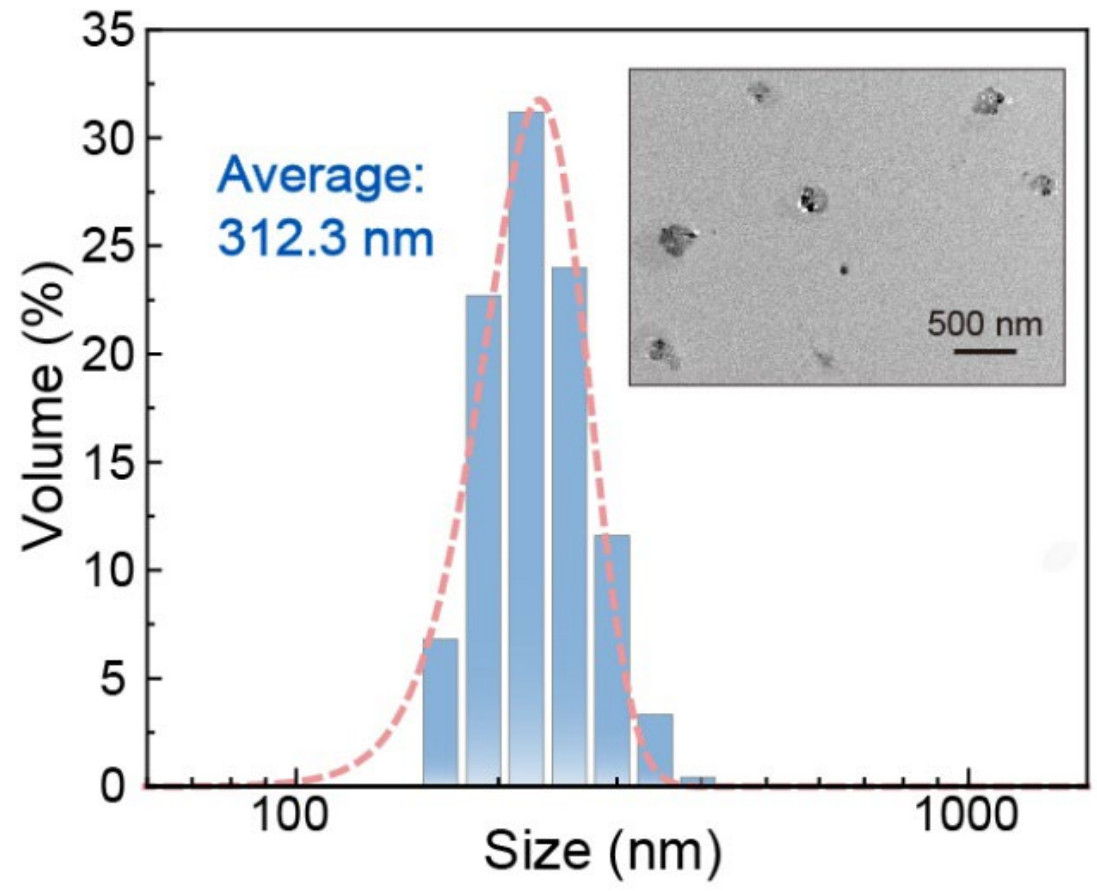

Supplementary Fig. 10 Size distribution of miRNA nanoparticles. The inset was the transmission electron microscope (TEM) image of miRNA nanoparticles.

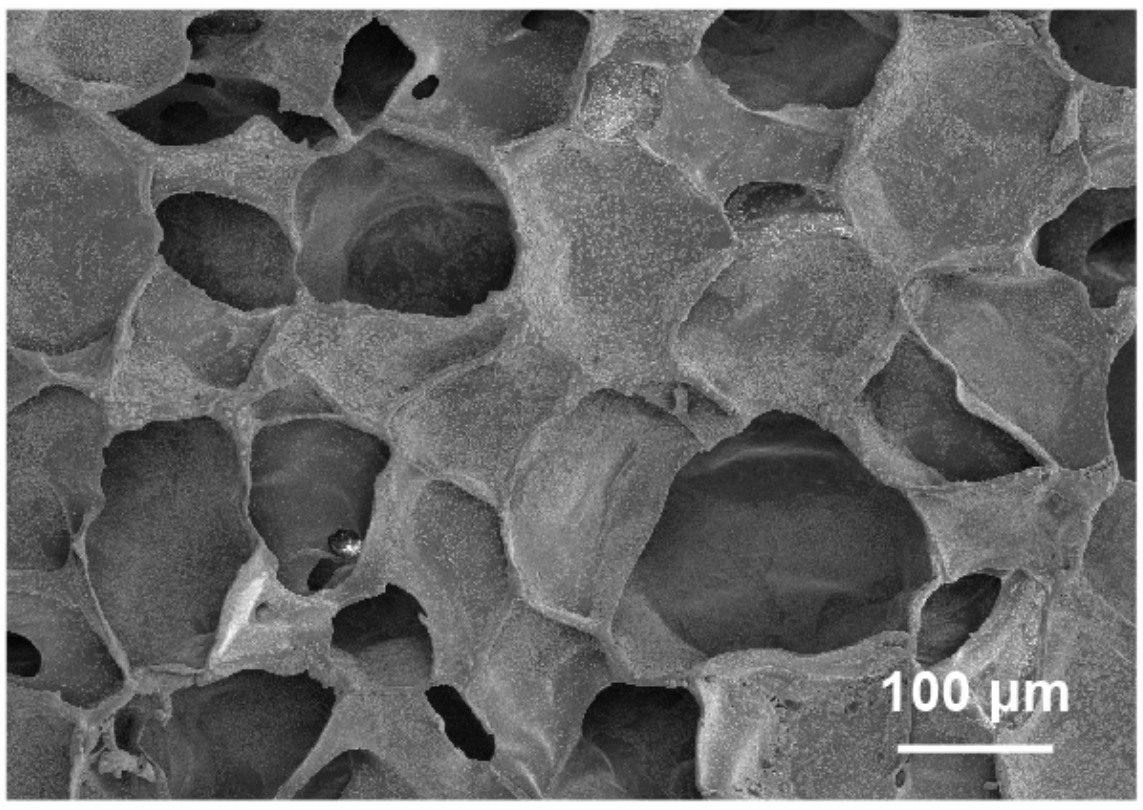

Supplementary Fig. 11 SEM image of the bioresponsive hydrogel.

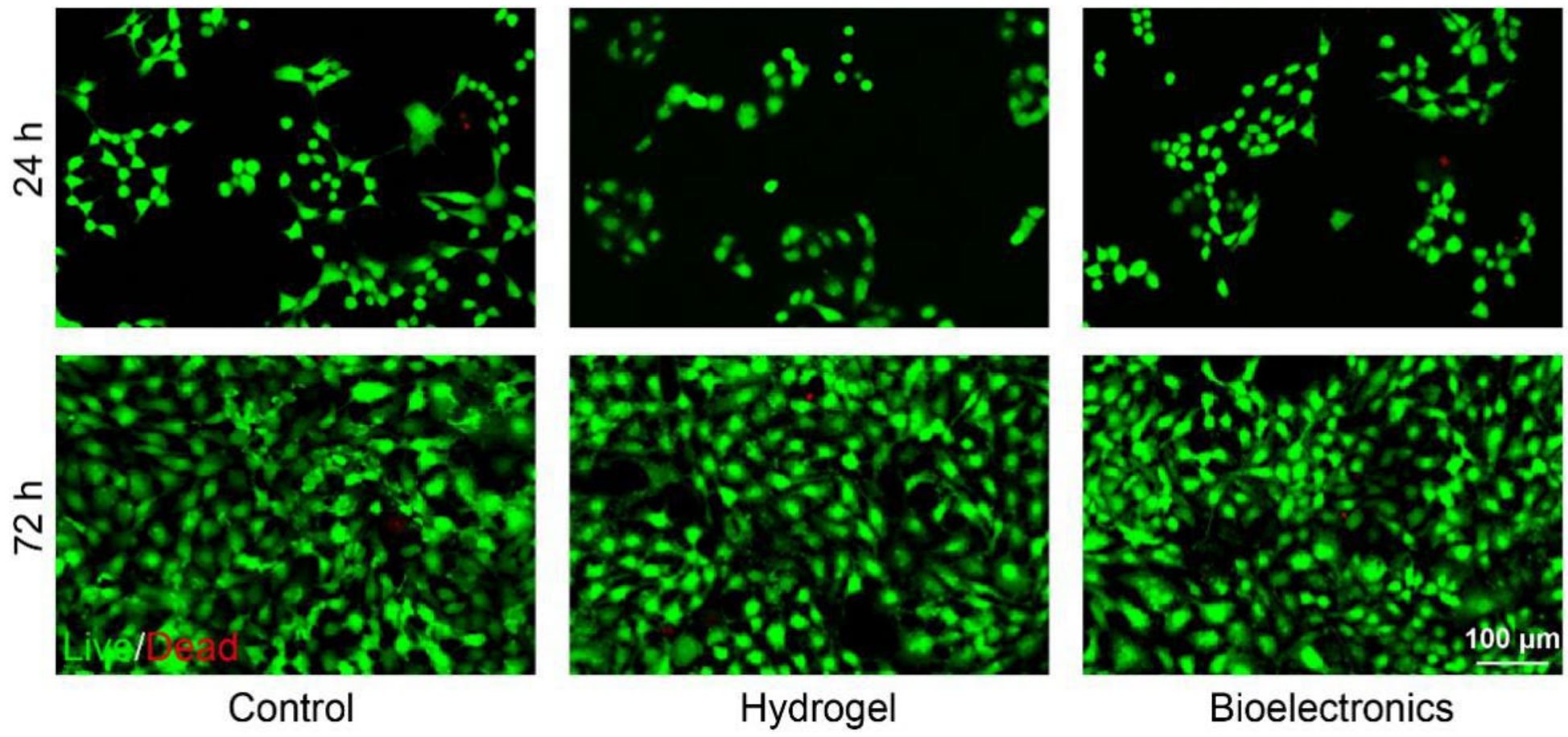

Supplementary Fig. 12 Representative live/dead fluorescent images of human keratinocytes (HaCaT) cells after 24 h and 72 h coincubation with the hydrogel and the WIRES. The live cells were stained green, and the dead cells were stained red.

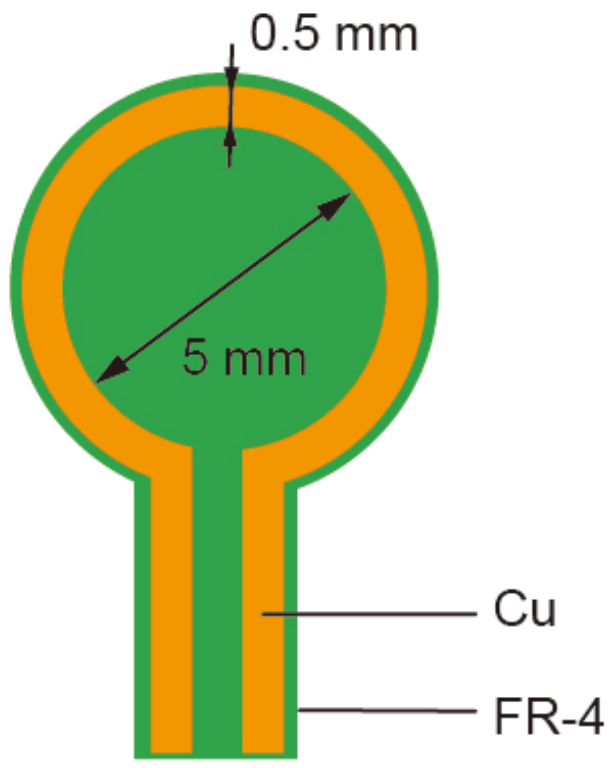

Supplementary Fig. 13 Geometry and design parameters of the readout coil.

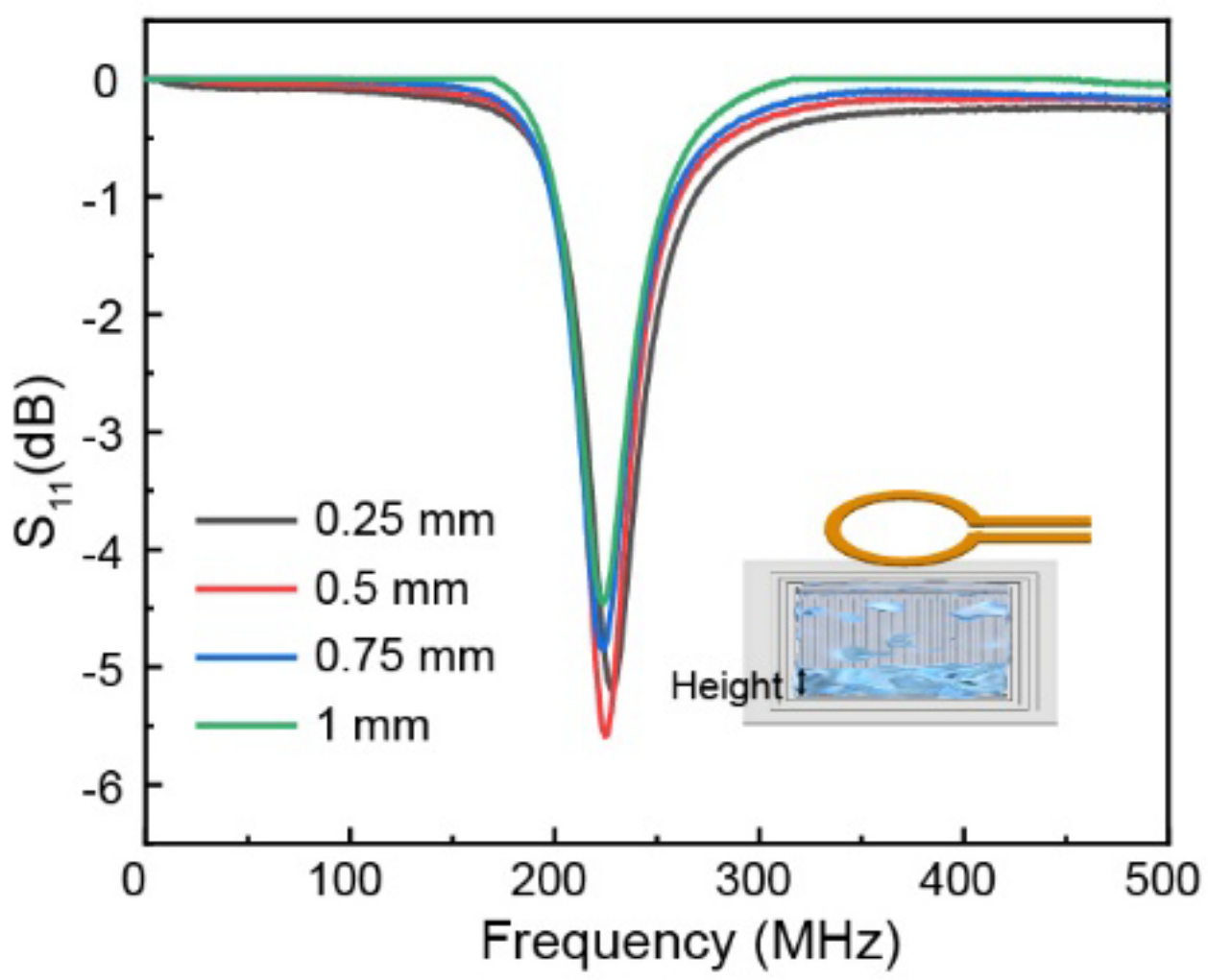

Supplementary Fig. 14 Influence of different heights of hydrogels with the same area.

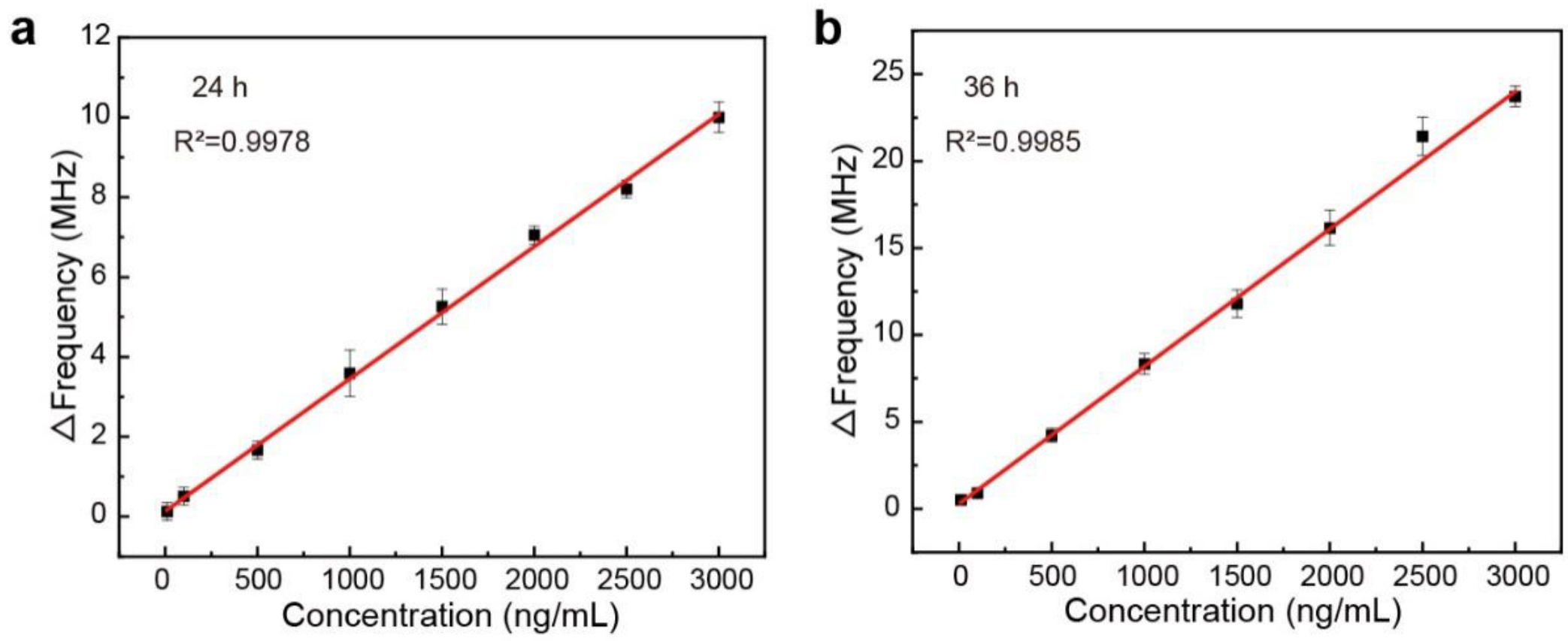

Supplementary Fig. 15 Calibration plot of frequency shifts versus MMP-9 concentration at 24 h (a) and 36 h (b).

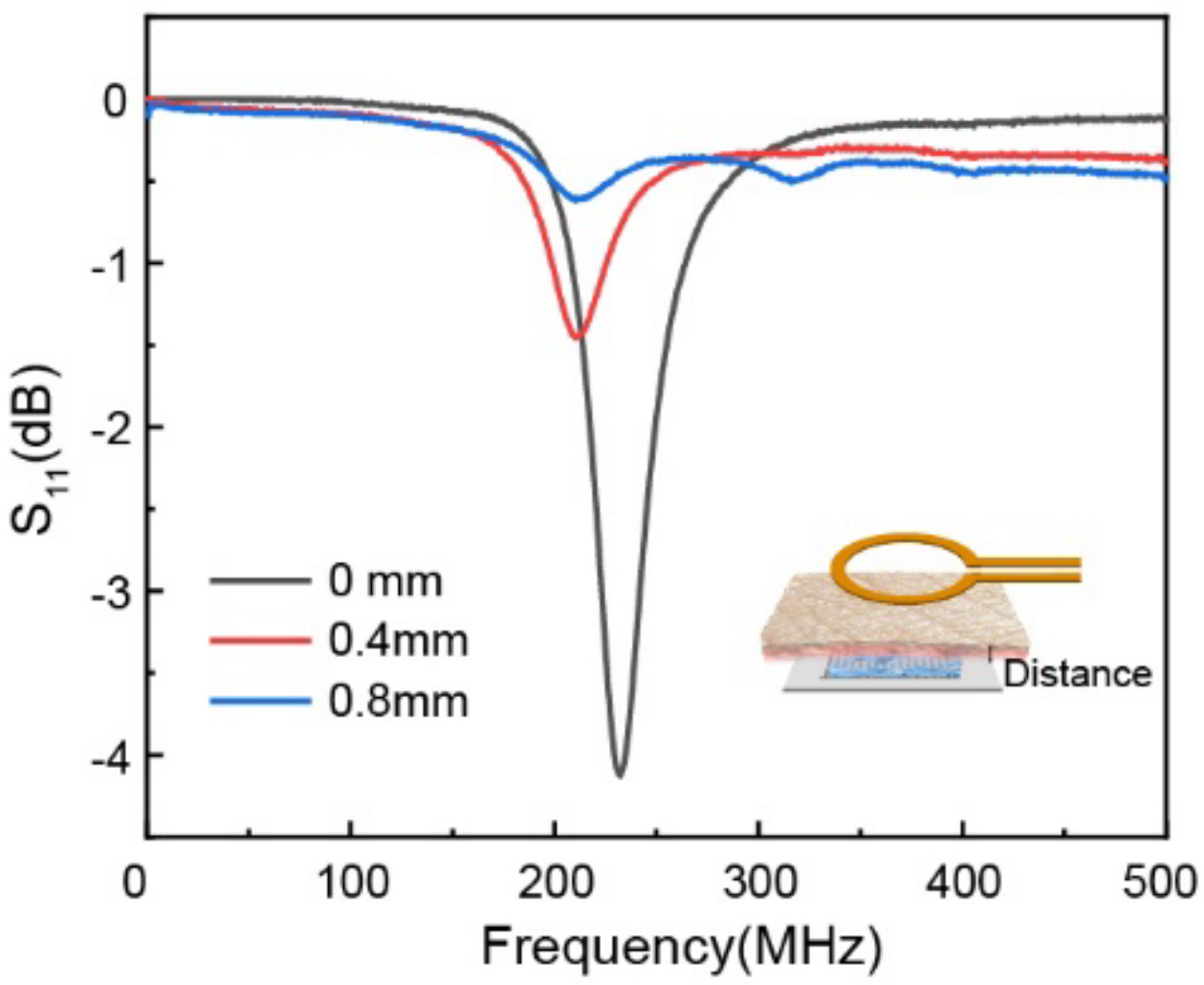

Supplementary Fig. 16 Influence of the tissue between the WIRES and the readout coil.

The resonant signals could be wirelessly transmitted through the tissue (in this case meat) via inductive coupling. The resonant frequency shifted in the presence of the tissue, but remained stable with different thicknesses of tissue.

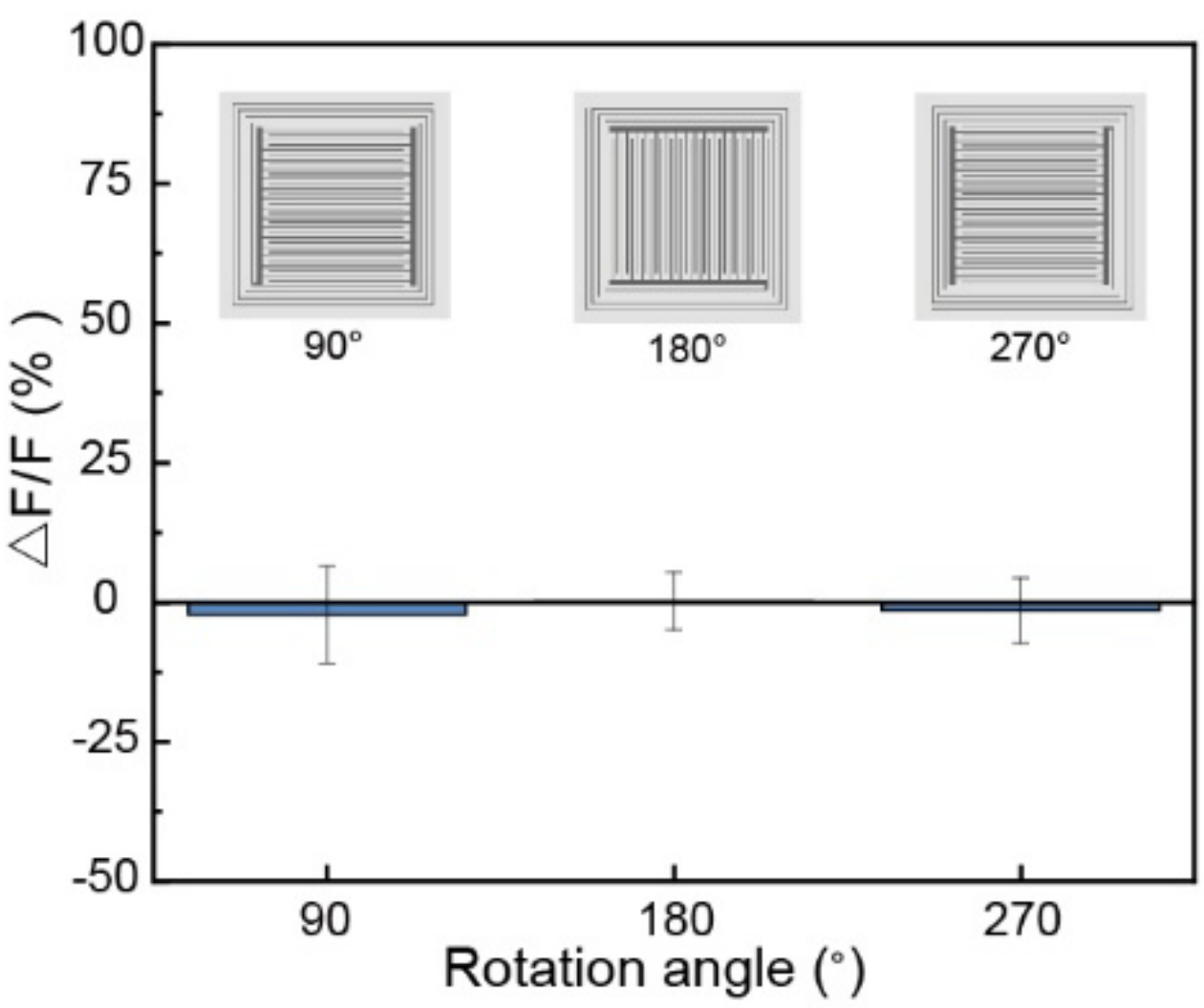

Supplementary Fig. 17 Influence of rotation angles of the WIRES relative to the readout coil on the signal.

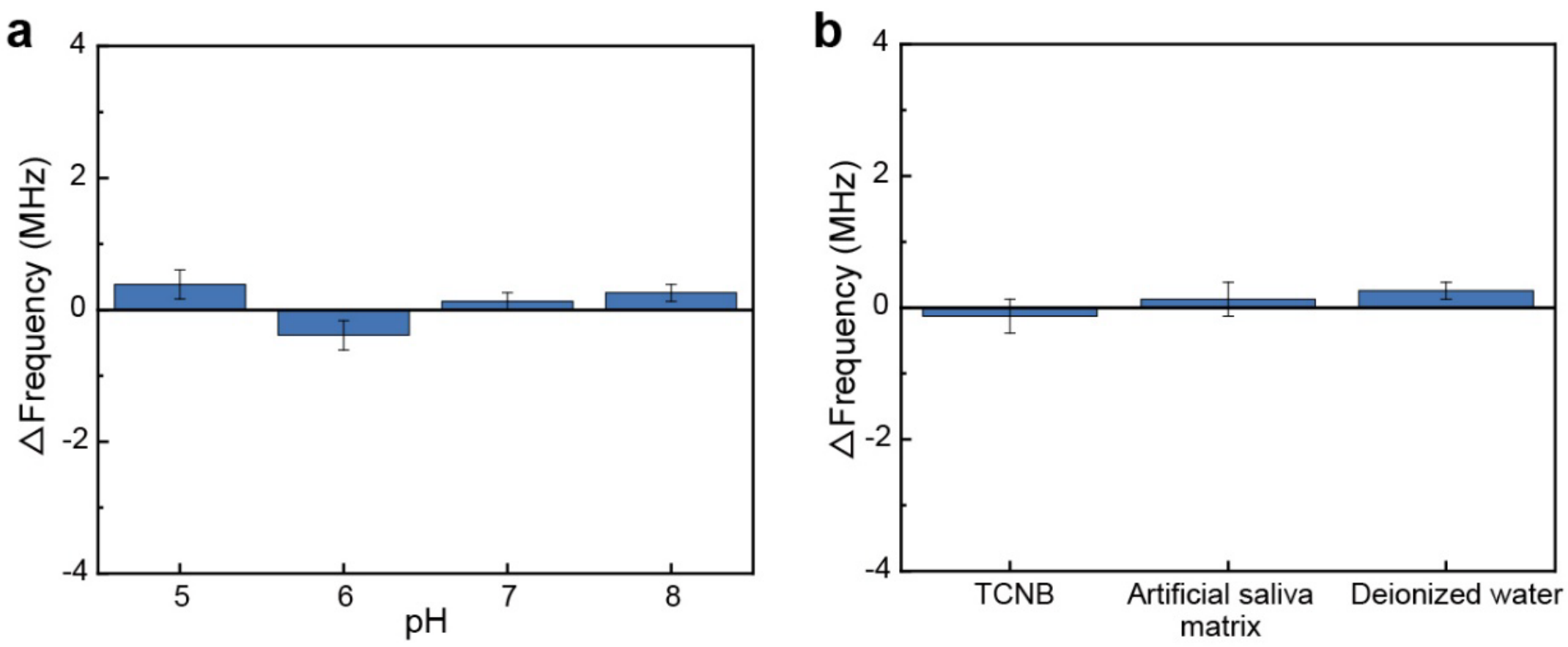

Supplementary Fig. 18 Resonant responses of MMP-9 biosensors to different pH (a) and different buffers (b).

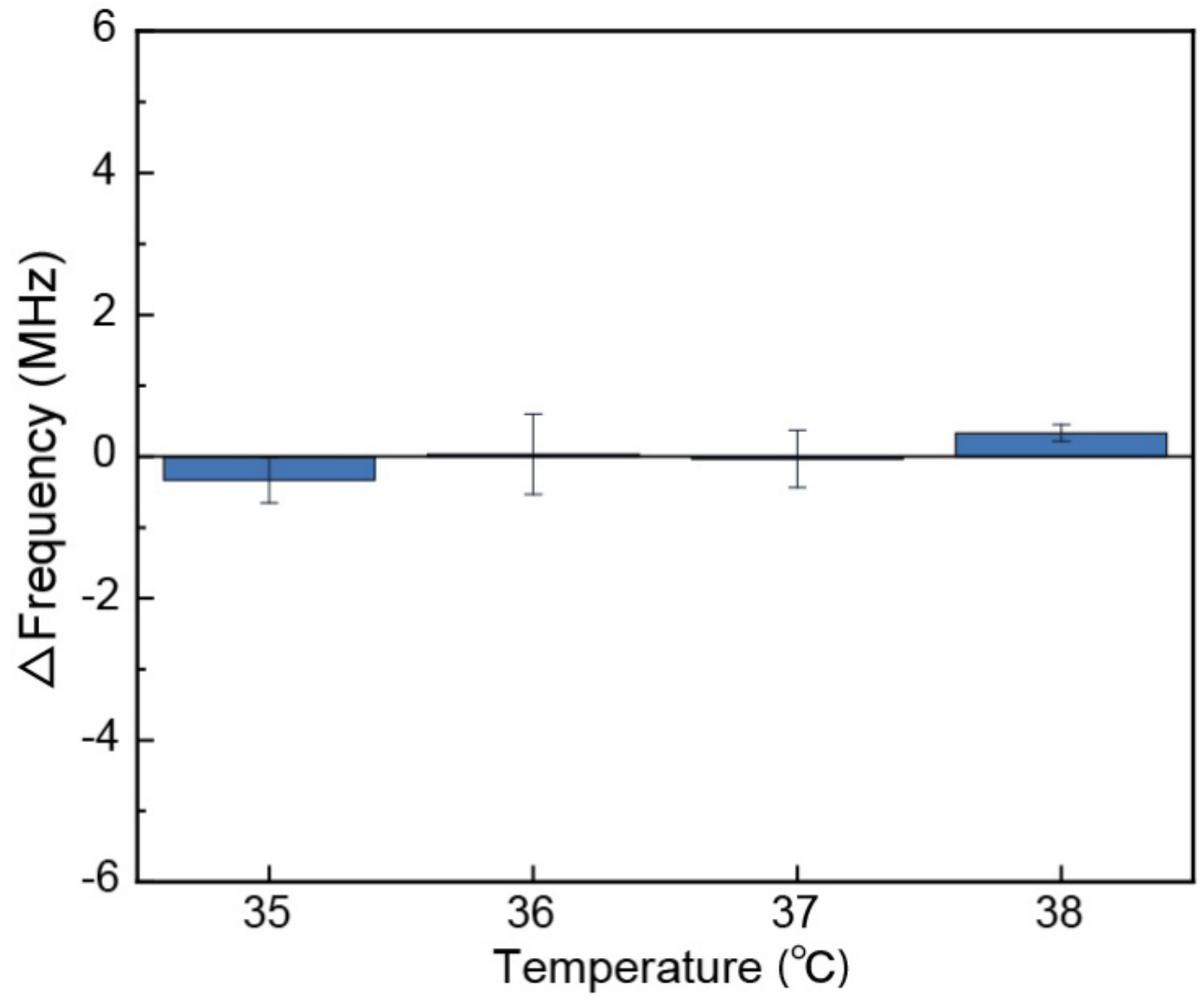

Supplementary Fig. 19 Resonant responses of MMP-9 biosensors in different temperatures.

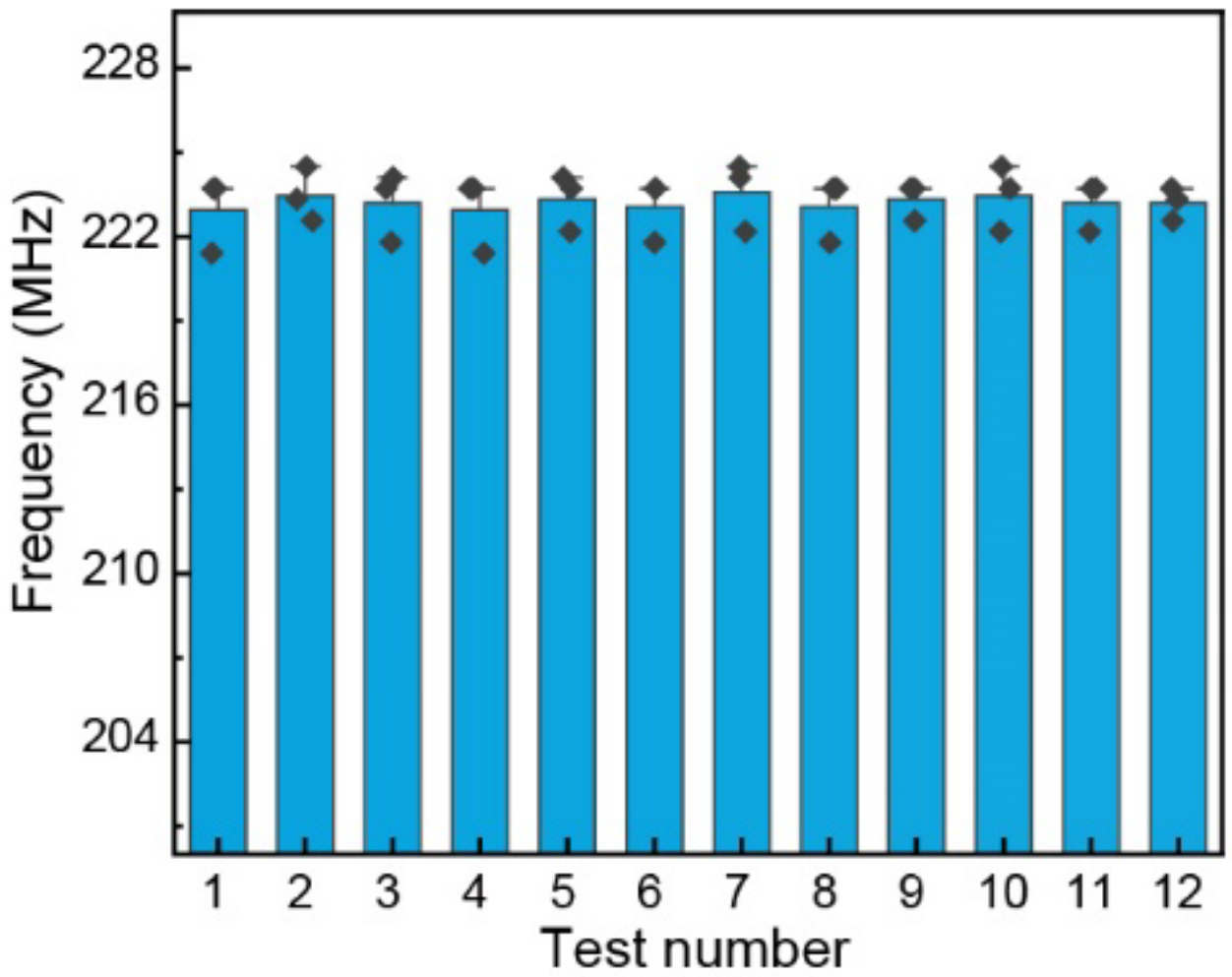

Supplementary Fig. 20 Repeatability tests of the MMP-9 biosensor.

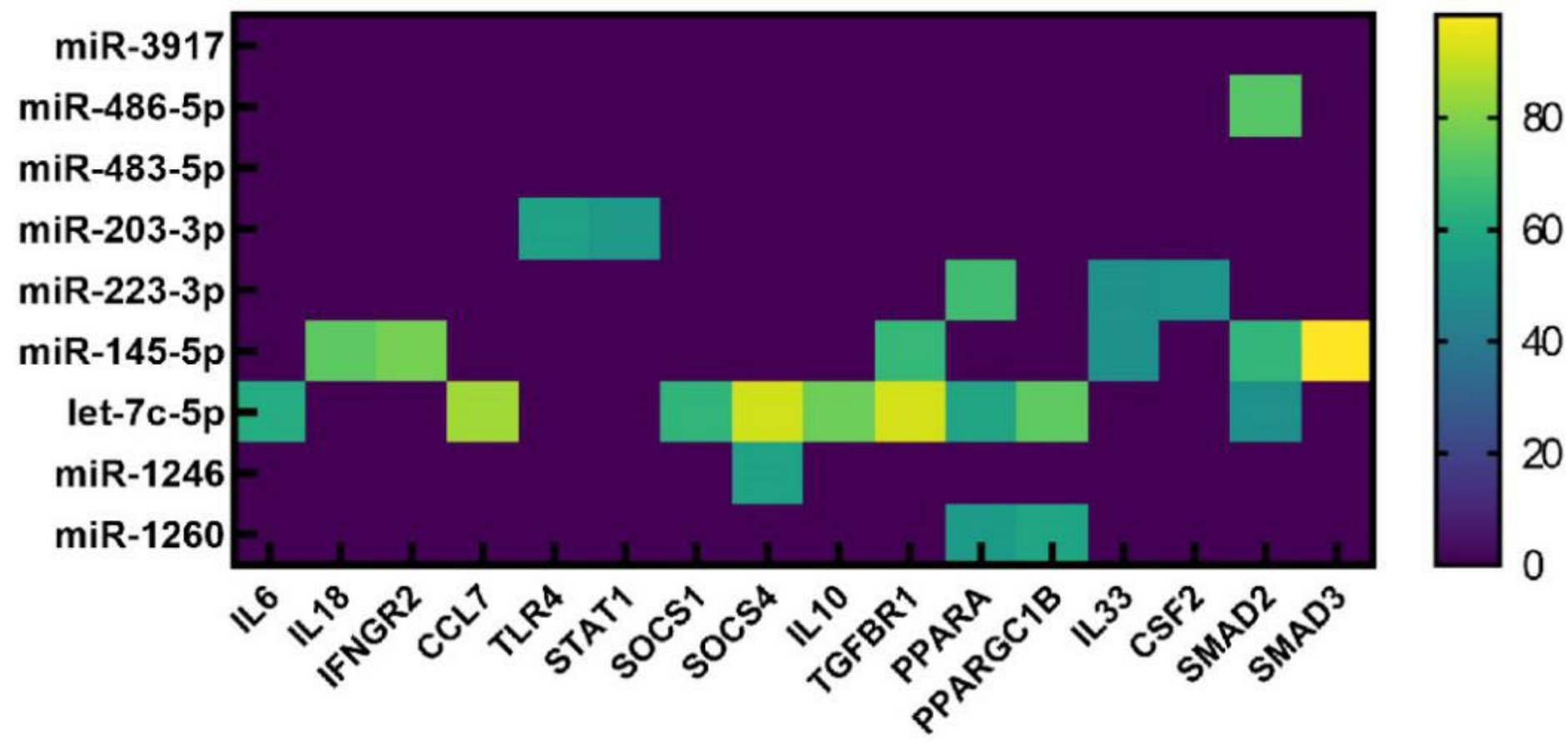

Supplementary Fig. 21 Heatmap for differential expression analysis of miRNAs in gingival tissues of patients with periodontitis and healthy individuals in the Gene Expression Omnibus (GEO) database.

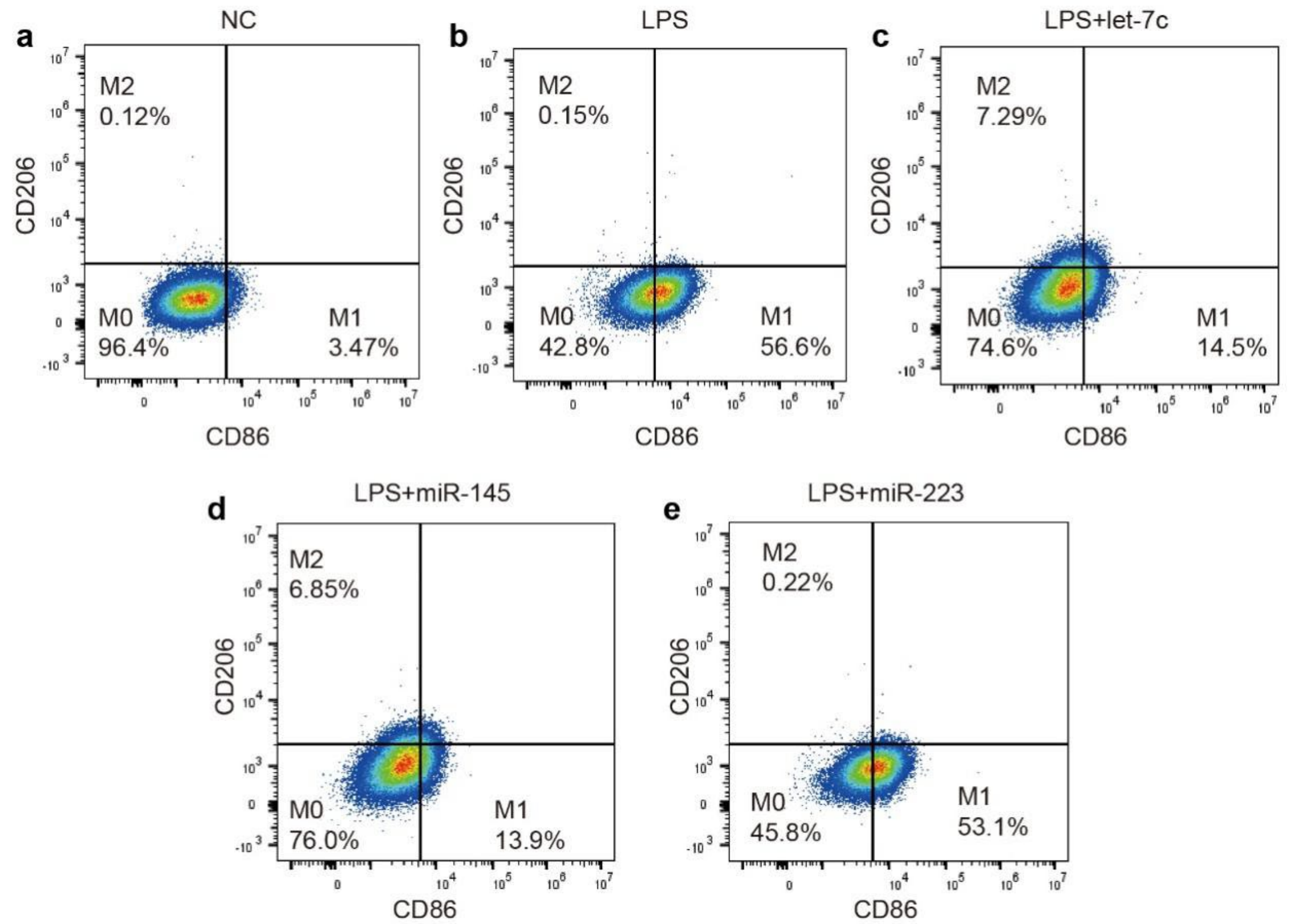

Supplementary Fig. 22 Flow cytometry plots of the impact of different miRNAs on the polarization of *Porphyromonas gingivalis* lipopolysaccharides (Pg-LPS) stimulated macrophages (RAW 264.7), including negative control (a), LPS stimulated (b), let-7c (c), miR-145 (d), and miR-223 (e).

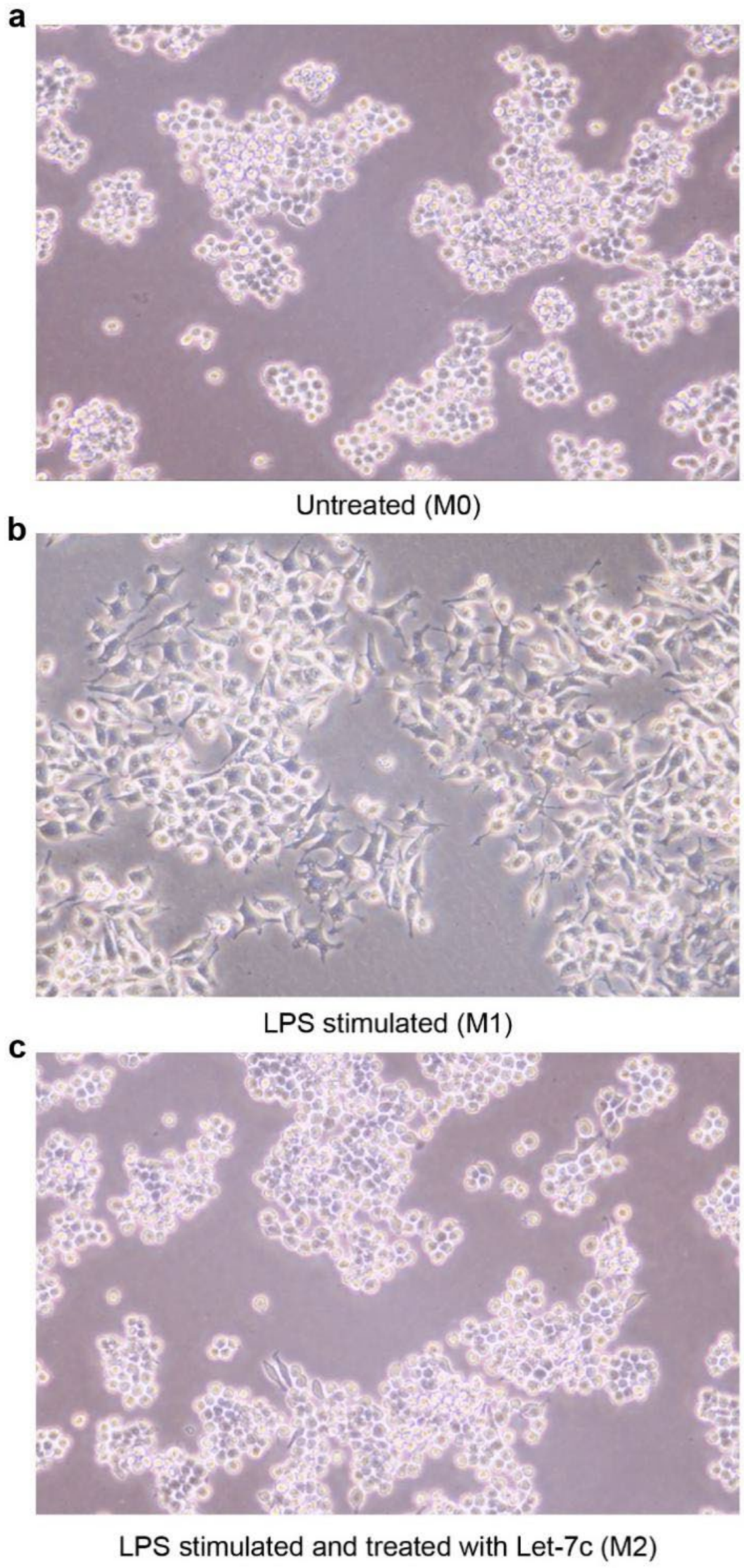

Supplementary Fig. 23 Representative microscopic images of macrophage polarization after different treatments, including M0 (a), M1 (b) and M2 (c).

Unteated macrophages (M0) were round and slightly oval. After stimulated by lipopolysaccharides (LPS), macrophages polarized into M1 pro-inflammatory, which exhibited irregular and dendritic appearance. When treated with Let-7c, macropahges were regulated to M2 anti-inflammatory phenotype, and showed a more flattened and less protrusive morphology.

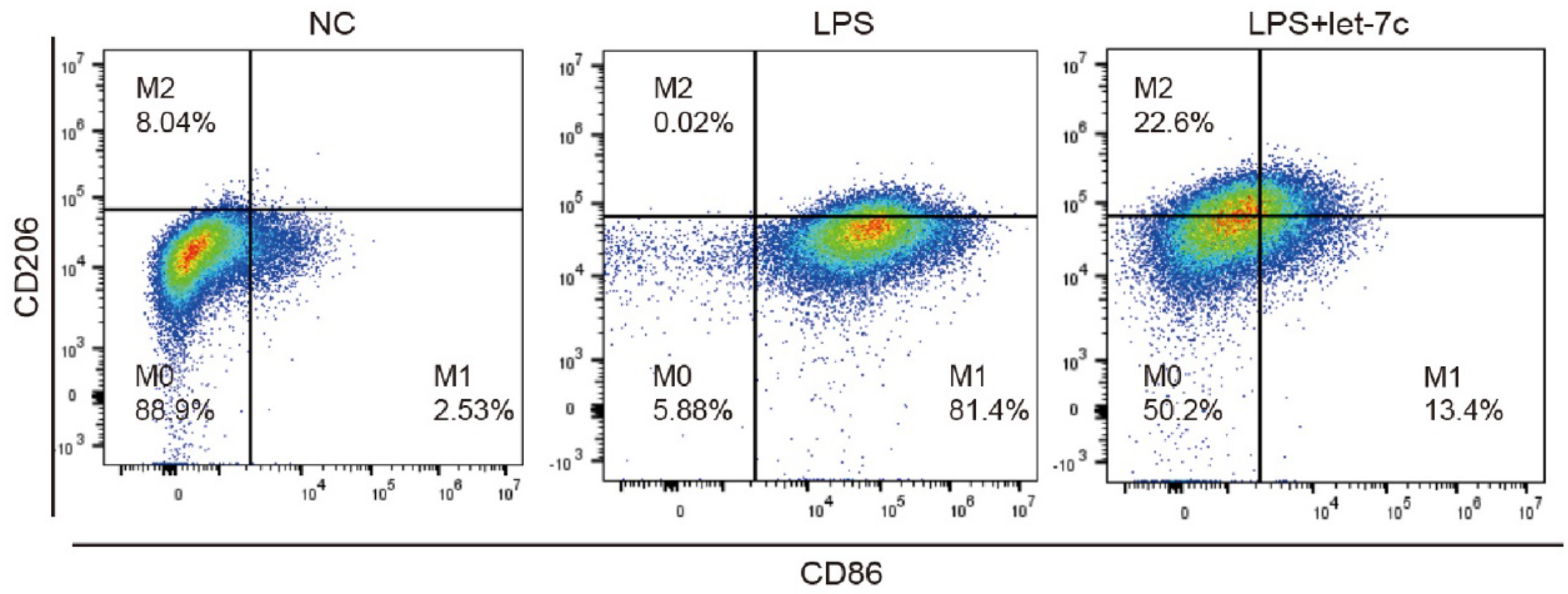

Supplementary Fig. 24 Flow cytometry analysis illustrating the impact of let-7c on the polarization of human macrophages.

THP-1 cell-derived macrophages were stimulated with LPS and treated with let-7c. The x-axis represented the fluorescence intensity of CD86 (an M1 polarization marker), and the y-axis represented CD206 (an M2 polarization marker). The results demonstrated that let-7c effectively suppresses LPS-induced M1 polarization and promotes M2 macrophage reprogramming in human cells.

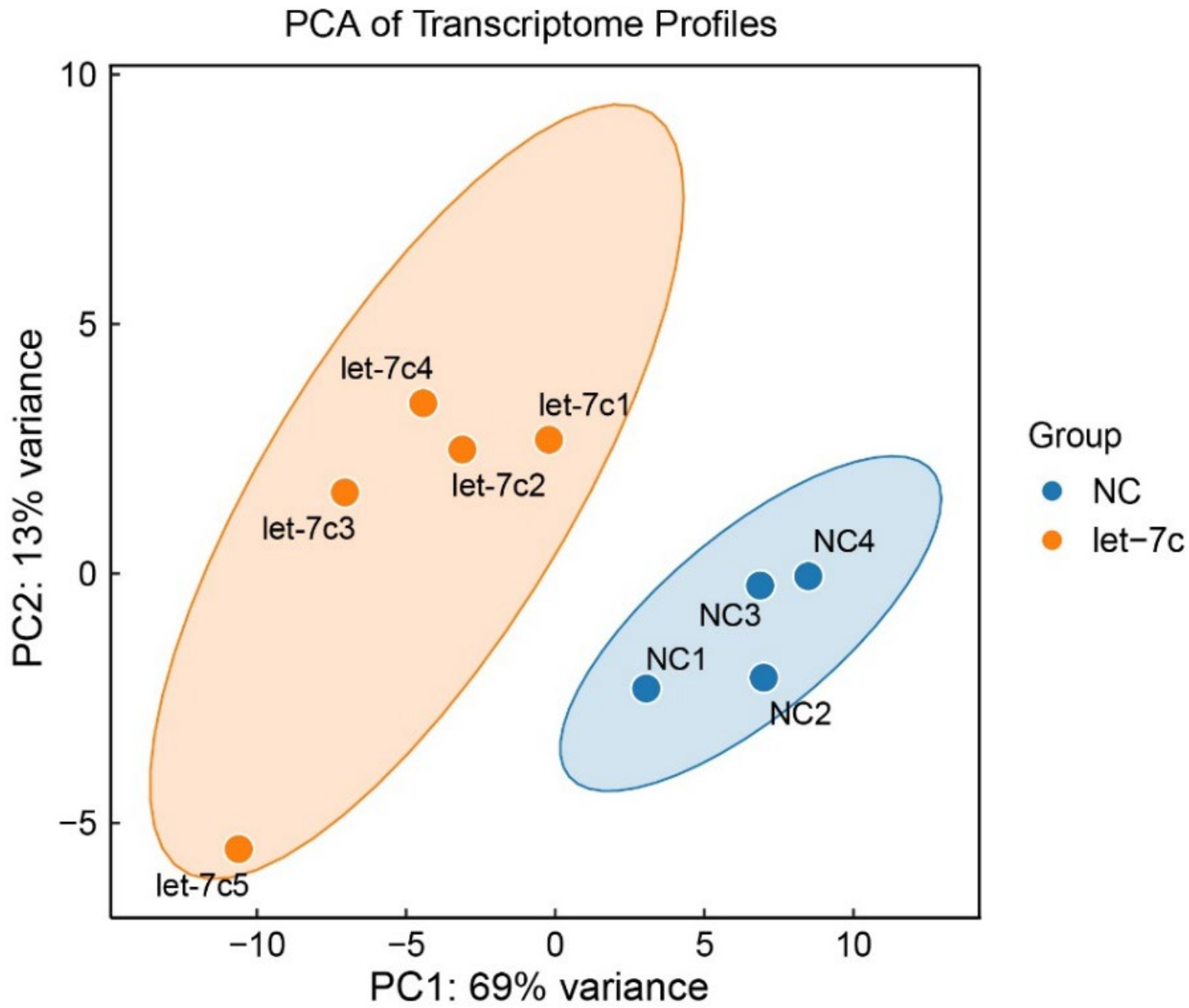

Supplementary Fig. 25 Principal Component Analysis (PCA) of RNA-seq samples.

The PCA plot visualizes the distribution of samples based on their gene expression profiles. The x-axis and y-axis represent the first (PC1) and second (PC2) principal components, accounting for 69% and 13% of the total variance, respectively. The clear separation between the let-7c-5p mimic-treated group (let7c, n=5) and the negative control group (NC, n=4) indicates distinct transcriptomic patterns between the two conditions and high consistency among biological replicates.

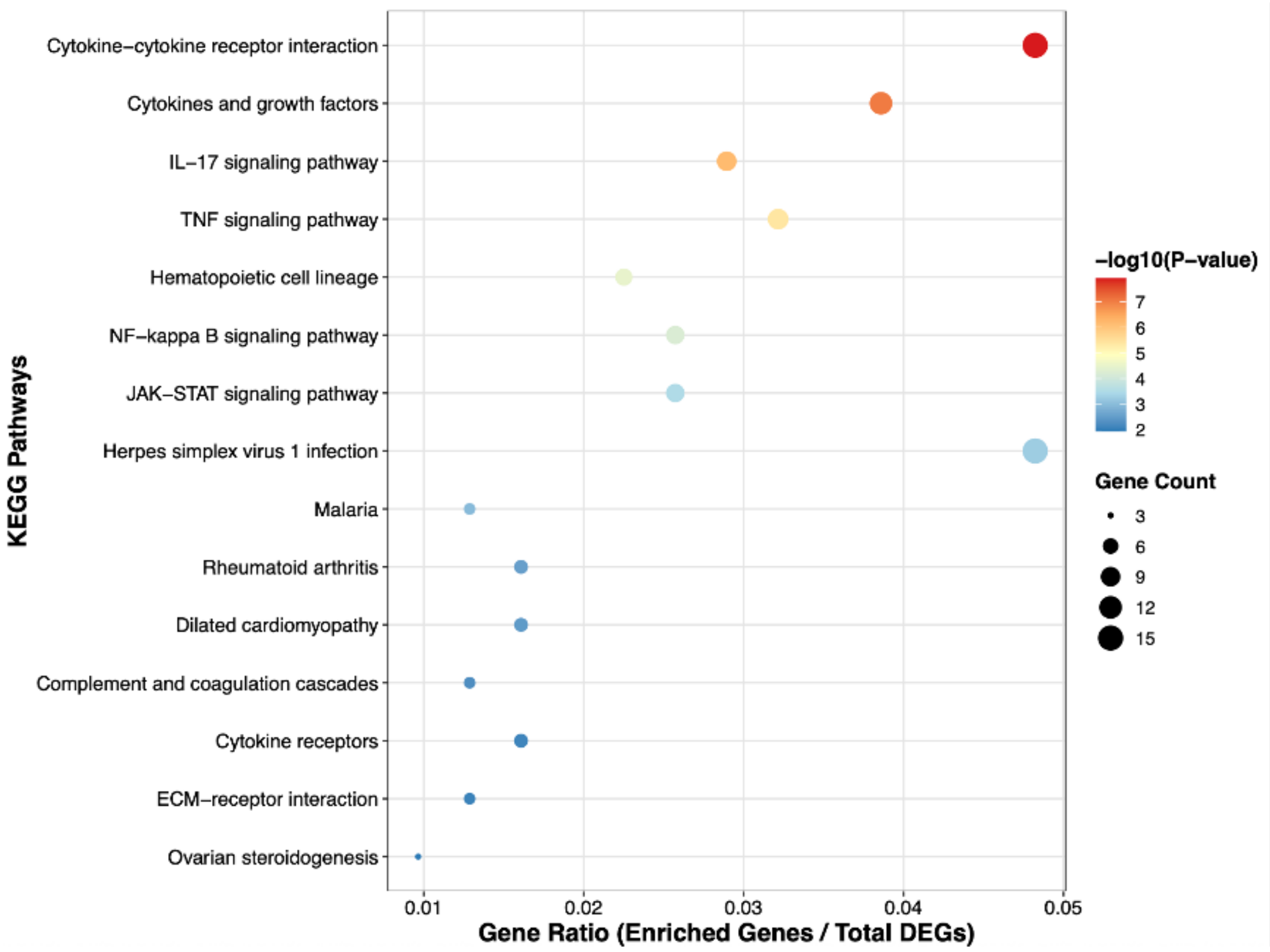

Supplementary Fig. 26 KEGG pathway enrichment analysis of differentially expressed genes (DEGs) in let-7c-5p overexpressing macrophages.

The bubble plot illustrated the top 15 enriched KEGG pathways for DEGs identified between let-7c-5p mimic-treated RAW 264.7 cells and the negative control (NC) group. The x-axis represented the Gene Ratio (the number of enriched genes divided by the total number of DEGs). The size of each dot corresponded to the number of genes enriched in the respective pathway (Gene Count), and the color gradient indicated the statistical significance (-log10 P-value). The analysis was performed using the hypergeometric test. Major immunomodulatory pathways, including Cytokine-cytokine receptor interaction, TNF signaling pathway, and NF-kappa B signaling pathway exhibited significant enrichment ($p < 0.05$).

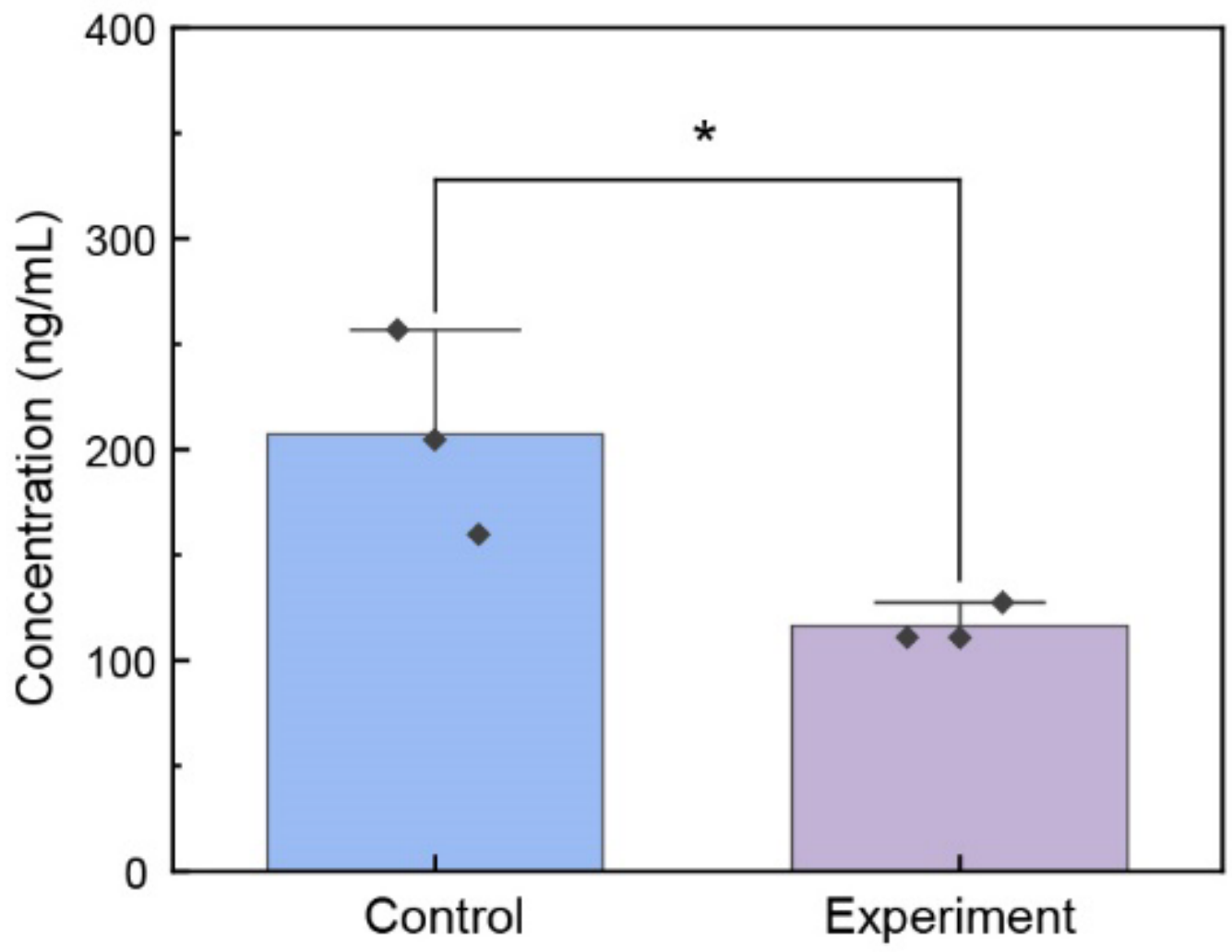

Supplementary Fig. 27 MMP-9 concentrations in macrophage culture samples after Pg-LPS stimulation and let-7c treatment measured by the WIRES. * $p < 0.05$.

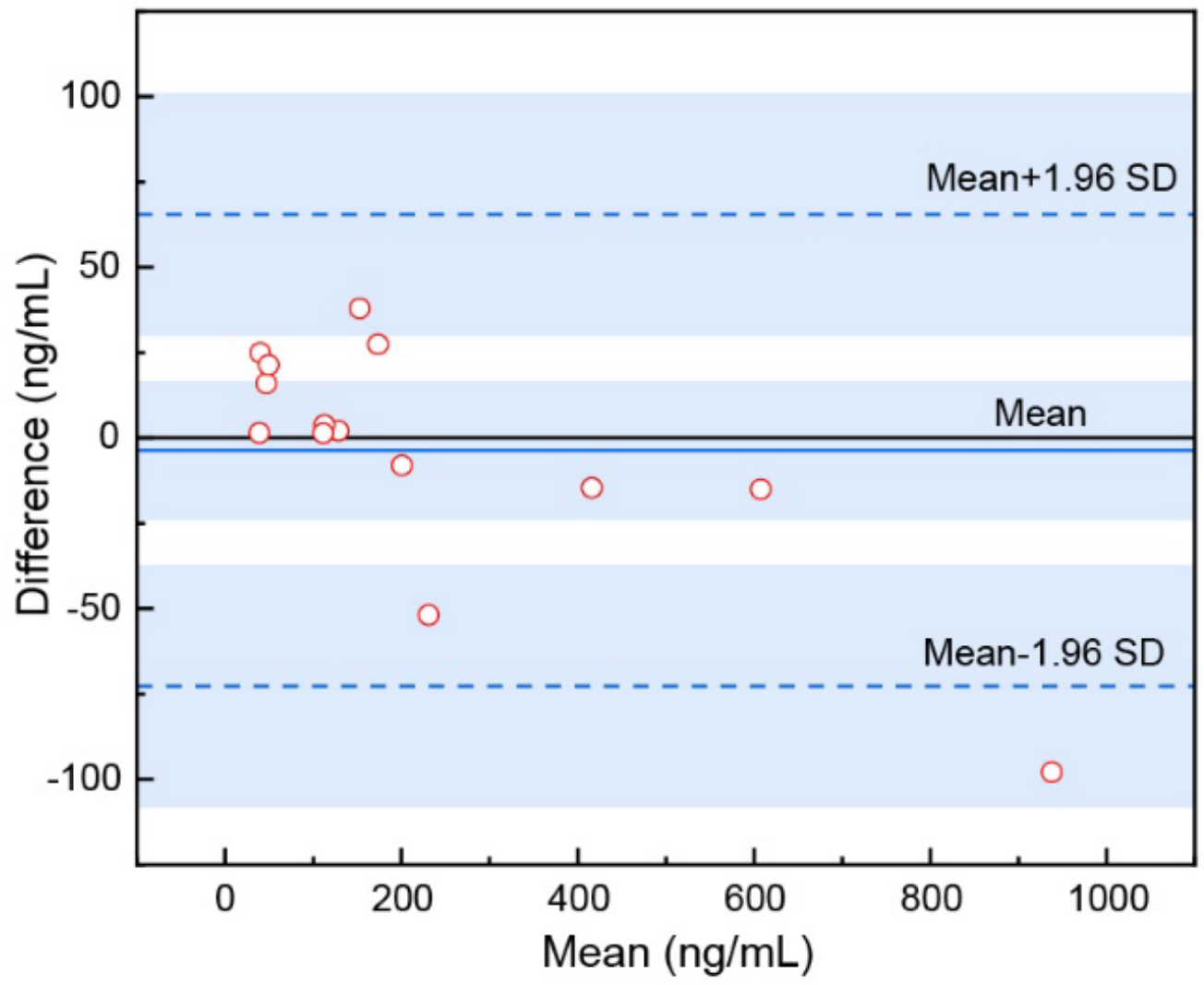

Supplementary Fig. 28 Bland-Altman plot showing the agreement between the MMP-9 biosensor and commercially available Active MMP-9 Fluorescent Assay.

Supplementary Table 1 Genes associated with macrophage maturation and differentiation.

Gene Symbol	Gene Type
IL6	Interleukin, key genes for M1 macrophages
IL18R1	Interleukin
IFNGR2	IFN-γ receptor
CCL7	Chemokine
TLR4	Toll-like Receptor
STAT1	Transcription Factor
SOCS1	Suppressor of Cytokine Signaling Family
SOCS4	Suppressor of Cytokine Signaling Family
IL10	Interleukin, key genes for M2 macrophages
TGFBR1	TGF-β Receptor
PPARA	Transcription Factor
PPARGC1B	Energy Metabolism Receptor, key genes for M2 macrophages
IL33	Interleukin
CSF2	Key Growth Factor for macrophages
SMAD2	Transcription Factor in TGF-β Signaling
SMAD3	Transcription Factor in TGF-β Signaling

Supplementary Table 2 Periodontal status of volunteers diagnosed by the dentist and corresponding salivary MMP-9 concentrations detected by the WIRES.

Volunteers	Sex	Age	Periodontal status of volunteers diagnosed by dentist	Degree	MMP-9 detected by the WIRES (ng/mL)
P. 1	Male	47	Periodontitis	Severe	986.6
P. 2	Male	43	Periodontitis	Moderate	615.0
P. 3	Male	35	Periodontitis	Moderate	423.2
P. 4	Female	32	Gingivitis	NA	133.5
H. 1	Male	23	Healthy	NA	38.8
H. 2	Female	26	Healthy	NA	38.8
H. 3	Female	30	Healthy	NA	27.4
H. 4	Male	27	Healthy	NA	38.0

Supplementary Table 3 Validation of in situ MMP-9 measurements using a commercial MMP-9 assay

Subject	MMP-9 detected by commercial assay (ng/mL)	MMP-9 detected by the WIRES (ng/mL)
Periodontitis before treatment	1173.8	1132.0
Periodontitis after treatment	434.1	456.4
Healthy volunteer	32.5	Below in situ quantification threshold

Supplementary Table 4 Comparison of MMP-9 sensing method for periodontal detection and this work.

Sensing materials	Methods	Detection range	LOD	Device	Sample	Activity test	References
Antibody	ELISA	0.625-20 ng/mL	0.16 ng/mL	Microtiter plate reader	Saliva	No	1
Antibody	Fluorescence	100-1000 ng/mL	100 ng/mL	Fluorescence microscopy	Saliva	No	2
Antibody	Fluorescence	5.12-500 ng/mL	8.62 ng/mL	Automated analyzer	Saliva	No	3
Antibody	Fluorescence	0.47-30 ng/mL	0.38 ng/mL	Microtiter plate reader	Saliva	No	4
Antibody	SPR	10-200 ng/mL	8 pg/mL	SPR system	Saliva	No	5
Antibody	Electrochemistry	0.03-2 ng/mL	13 pg/mL	Multipotentiostat	Plasma	No	6
Aptamer	Electrochemistry	50-1250 pg/mL	10.51 pg/mL	Electrochemical workstation	Saliva & Serum	No	7
Peptide	Fluorescence	3.5-25 ng/mL	1.7 ng/mL	Fluorescence Spectrometer	Saliva	Yes	8
Peptide	Electrochemistry	100-1200 ng/mL	75 ng/mL	Electrochemical workstation	Saliva	Yes	9
**Peptide-based hydrogel**	**Radio frequency**	**10-3000 ng/mL**	**7.9 ng/mL**	**Portable VNA**	**Saliva**	**Yes**	**This work**

Abbreviations: LOD, limit of detection. ELISA, enzyme linked immunosorbent assay. SPR, surface plasmon resonance. VNA, vector network aAnalyzer.

Supplementary Table 5 Comparison of currently available local drug delivery systems for periodontitis treatment to our work.

Cargo	Scaffold	Control mode	Trigger mechanism	Release behavior	Treatment modality	Sensor integration	References
Chlorhexidine	Xanthan gel	Passive	None	Sustained	Antibacterial	No	10
Ciprofloxacin	Dual corona polymer vesicles	Passive	None	Sustained	Antibacterial	No	11
Exosome	Chitosan hydrogel	Passive	None	Sustained	Immunomodulation	No	12
Aspirin, erythropoietin	Chitosan/β-glycerophosphate/gelatin hydrogel	Passive	Material degradation	Sustained	Anti-inflammatory	No	13
Tetracycline, cytokines (IL-4, TGF-β)	Microneedle patch	Programmed	Material degradation	Initial burst, sustained	Antibacterial, immunomodulation	No	14
Melanin nanoparticles	Porous microparticles	External-triggered	NIR irradiation	On-demand	Antibacterial, anti-inflammatory	No	15
Copper sulfide nanozyme	Microneedle patch	External-triggered	NIR irradiation	On-demand	Antibacterial	No	16
Minocycline	ZIF-8 nanoparticles	Stimuli-responsive	pH-responsive	Triggered release	Antibacterial, anti-inflammatory	No	17
Embelin	Chitosan-dextran hydrogel	Stimuli-responsive	pH-responsive	Triggered release	Antibacterial, immunomodulation	No	18
**MicroRNA (let-7c)**	**Hydrogel-integrated bioelectronic system**	**Stimuli-responsive with wireless readout**	**MMP-responsive**	**Triggered release**	**Immunomodulation**	**Yes**	**This work**

Abbreviations: IL-4, interleukin-4. TGF-β, transforming growth factor beta. NIR, near-infrared. ZIF-8, zeolite imidazolate frameworks-8. MMP, matrix metalloproteinase.

Supplementary Table 6 Sequences of miRNA mimics and agomirs.

Name	Sequences
Mir-145 mimics	Forward: 5′-GUCCAGUUUUCCCAGGAAUCCCU-3′,<br>Reverse: 5′-GGAUUCCUGGGAAAACUGGACUU-3′
Mir-223-3p mimics	Forward: 5′- UAAGGCACGCGGUGAAUGCC-3′,<br>Reverse: 5′- CAUUCACCGCGUGCCUUAUU-3′
Let-7c-5p mimics	Forward: 5′- UGAGGUAGUAGGUUGUAUGGUU-3′,<br>Reverse: 5′- CCAUACAACCUACUACCUCAUU-3′
Let-7c-5p-FAM	Forward: 5′-FAM-GUCCAGUUUUCCCAGGAAUCCCU-3′,<br>Reverse: 5′-GGAUUCCUGGGAAAACUGGACUU -3′
Let-7c-5p agomirs	Forward: 5′-GUCCAGUUUUCCCAGGAAUCCCU-3′,<br>Reverse: 5′-AsCsGGAUUCCUGGGAAAACUGGACUUAsAsUsUs-Chol-3′

Supplementary Table 7 Primers used for RT-PCR.

Name	Sequences
Let-7c-5p-stem	GTCGTATCCAGTGCAGGGTCCGAGGTATTC [illegible]
Let-7c-5p-F	GCCGTGAGGTAGTAGGTTGT
Universal reverse primer	GTGCAGGGTCCGAGGT
U6-F	CAGCACAAAAGGAAACTCACC
MMP9-F	GCAGAGGCATACTTGTACCG
MMP9-R	TGATGTTATGATGGTCCCACTTG
iNOS-F	GTTCTCAGCCCAACAATACAAGA
iNOS-R	GTGGACGGGTCGATGTCAC
IL-6-F	CTTGGGACTG ATGCTGGTGAC
IL-6-R	TTCTCATTTCCACGATTTCCCA
Arg-1-F	CTCCAAGCCAAAGTCCTTAGAG
Arg-1-R	AGGAGCTGTCATTAGGGACATC
IL-10-F	GCTCT TACTGACTGGCATGAG
IL-10-R	CGCAGCTCTAGGAGCA TGTG
β-Actin-F	GGCATCGTGATGGACTCC
β-Actin-R	GCTGGAAGGTGGACAGCG

Reference:

1. Johannsen B, Muller L, Baumgartner D *et al.* Automated pre-analytic processing of whole saliva using magnet-beating for point-of-care protein biomarker analysis. *Micromachines (Basel)* 2019; **10**: 833.

2. Yee EH, Lathwal S, Shah PP *et al.* Detection of biomarkers of periodontal disease in human saliva using stabilized, vertical flow immunoassays. *ACS Sens* 2017; **2**: 1589-93.

3. Nie S, Henley WH, Miller SE *et al.* An automated integrated platform for rapid and sensitive multiplexed protein profiling using human saliva samples. *Lab Chip* 2014; **14**: 1087-98.

4. Johannsen B, Karpisek M, Baumgartner D *et al.* One-step, wash-free, bead-based immunoassay employing bound-free phase detection. *Anal Chim Acta* 2021; **1153**: 338280.

5. Mohseni S, Moghadam TT, Dabirmanesh B *et al.* Development of a label-free SPR sensor for detection of matrixmetalloproteinase-9 by antibody immobilization on carboxymethyldextran chip. *Biosens Bioelectron* 2016; **81**: 510-16.

6. Ruiz-Vega G, Garcia-Robaina A, Ben Ismail M *et al.* Detection of plasma MMP-9 within minutes. Unveiling some of the clues to develop fast and simple electrochemical magneto-immunosensors. *Biosens Bioelectron* 2018; **115**: 45-52.

7. Ansari MA, Mohd-Naim NF, Ahmed MU. Electrochemical nanoaptasensor based on graphitic carbon nitride/zirconium dioxide/multiwalled carbon nanotubes for matrix metalloproteinase-9 in human serum and saliva. *ACS Appl Bio Mater* 2024; **7**: 1579-87.

8. Gou X, Nawaz MAH, Liu C *et al.* Polypeptide induced perylene probe excimer formation and its application in the noncovalent ratiometric detection of matrix metalloproteinase activity. *J Mater Chem B* 2022; **10**: 5774-83.

9. Valero-Calvo D, Toyos-Rodriguez C, Garcia-Alonso FJ *et al.* Electrochemical monitoring of enzymatic cleavage in nanochannels with nanoparticle-based enhancement: determination of MMP-9 biomarker. *Mikrochim Acta* 2023; **190**: 257.

10. Paolantonio M, D'Ercole S, Pilloni A *et al.* Clinical, microbiologic, and biochemical effects of subgingival administration of a Xanthan-based chlorhexidine gel in the treatment of periodontitis: a randomized multicenter trial. *J Periodontol* 2009; **80**: 1479-92.

11. Xi Y, Wang Y, Gao J *et al.* Dual corona vesicles with intrinsic antibacterial and enhanced antibiotic delivery capabilities for effective treatment of biofilm-induced periodontitis. *ACS Nano* 2019; **13**: 13645-57.

12. Shen Z, Kuang S, Zhang Y *et al.* Chitosan hydrogel incorporated with dental pulp stem cell-derived exosomes alleviates periodontitis in mice via a macrophage-dependent mechanism. *Bioact Mater* 2020; **5**: 1113-26.

13. Xu X, Gu Z, Chen X *et al.* An injectable and thermosensitive hydrogel: Promoting periodontal regeneration by controlled-release of aspirin and erythropoietin. *Acta Biomater* 2019; **86**: 235-46.

14. Zhang X, Hasani-Sadrabadi MM, Zarubova J *et al.* Immunomodulatory Microneedle Patch for Periodontal Tissue Regeneration. *Matter* 2022; **5**: 666-82.
15. Song C, Wu X, Wang Y *et al.* Cuttlefish-inspired photo-responsive antibacterial microparticles with natural melanin nanoparticles spray. *Small* 2024; **20**: e2310444.
16. Chen H, Yu N, Wang J *et al.* Construction of versatile fibroin/nanozyme hybrid microneedles with controllable phototherapeutic sterilization property against periodontitis. *Nano Today* 2024; **56**: 102297.
17. Li Y, Xu C, Mao J *et al.* ZIF-8-based nanoparticles for inflammation treatment and oxidative stress reduction in periodontitis. *ACS Appl Mater Interfaces* 2024; **16**: 36077-94.
18. Cai G, Ren L, Yu J *et al.* A microenvironment-responsive, controlled release hydrogel delivering embelin to promote bone repair of periodontitis via anti-infection and osteo-immune modulation. *Adv Sci (Weinh)* 2024; **11**: e2403786.
